# Author Correction: Harnessing the acceptor substrate promiscuity of *Clostridium botulinum* Maf glycosyltransferase to glyco-engineer mini-flagellin protein chimeras

**DOI:** 10.1038/s42003-025-07774-w

**Published:** 2025-02-28

**Authors:** Sonali Sunsunwal, Aasawari Khairnar, Srikrishna Subramanian, T.N.C. Ramya

**Affiliations:** CSIR- Institute of Microbial Technology, Sector 39-A, Chandigarh, 160036 India

**Keywords:** Glycobiology, Transferases

Correction to: *Communications Biology* 10.1038/s42003-024-06736-y, published online 21 August 2024

In the version of the article initially published, the panels v–x of the pLogo analysis in Supplementary Fig. 3 were incorrect and have now been replaced in the Supplementary Information of the article.

