# Supplementary material for: Harnessing the acceptor substrate promiscuity of Clostridium botulinum Maf glycosyltransferase to glyco-engineer mini-flagellin protein chimeras
Source: Commun Biol. 2024 Aug 21;7:1029. doi: 10.1038/s42003-024-06736-y (PMC11339370; doi:10.1038/s42003-024-06736-y)
Supplement: Supplementary file 2 — Supplementary Information [file 42003_2024_6736_MOESM2_ESM.pdf]

Supplementary Information

Harnessing the acceptor substrate promiscuity of *Clostridium botulinum* Maf glycosyltransferase to glyco-engineer mini-flagellin protein chimeras

Sonali Sunsunwal, Aasawari Khairnar, Srikrishna Subramanian, and T.N.C. Ramya*
CSIR- Institute of Microbial Technology, Sector 39-A, Chandigarh 160036, INDIA

*Correspondence to be addressed to
T.N.C. Ramya, CSIR- Institute of Microbial Technology, Sector 39-A, Chandigarh 160036, INDIA.
Tel: 91-172-2880243; E-mail: ramya@imtech.res.in

Running Title: Maf glycosylates mini-flagellin protein chimeras

Keywords: Maf glycosyltransferase, flagellin, glyco-engineering, sialylation, substrate promiscuity, acceptor substrate, protein chimeras

This Supplementary Information file includes eight supplementary figures. A separate Supplementary Data 1 file with forty-seven supplementary tables, a Supplementary Data 2 zipped folder of AlphaFold2-predicted structural models of the protein constructs in this paper, and a Supplementary Data 3 zipped folder of source data behind all the graphs in this paper also accompany this manuscript.

Supplementary Figure 1

a. Singly expressed *Cb*Fla

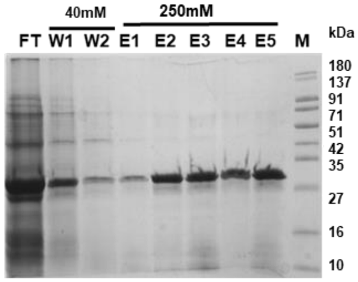

b. *Cb*Fla co-expressed with *Cb*Maf

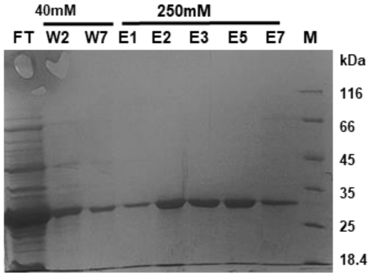

c. Singly expressed *Cb*FlaA1

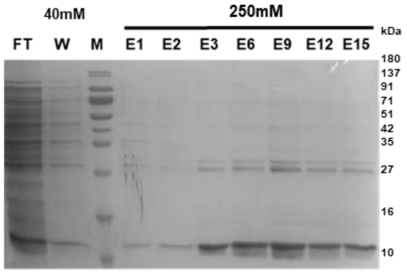

d. *Cb*FlaA1 co-expressed with *Cb*Maf

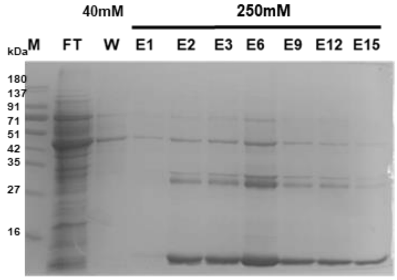

e. Singly expressed *Cb*FlaA3

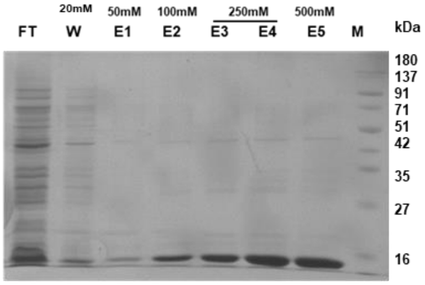

f. *Cb*FlaA3 co-expressed with *Cb*Maf

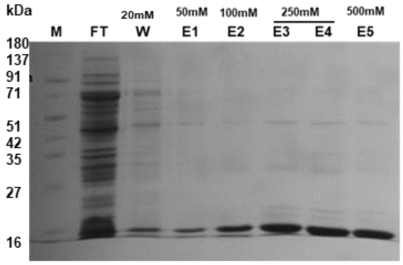

g. Singly expressed *Cb*FlaA4

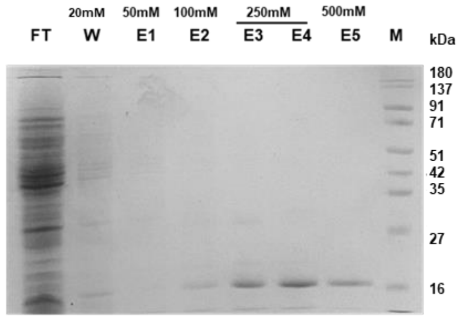

h. *Cb*FlaA4 co-expressed with *Cb*Maf

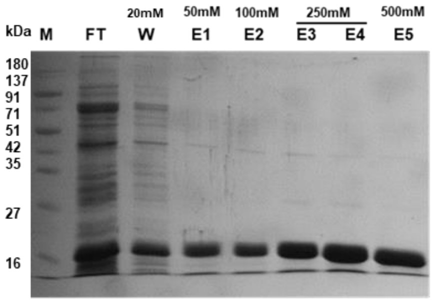

i. Singly expressed *Cb*FlaA5

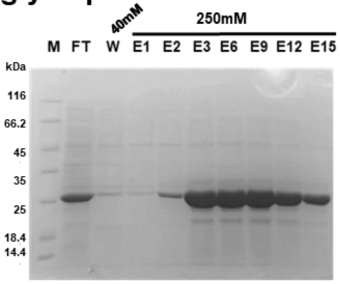

j. *Cb*FlaA5 co-expressed with *Cb*Maf

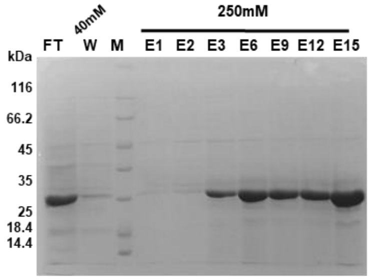

k. Singly expressed *Cb*FlaA7

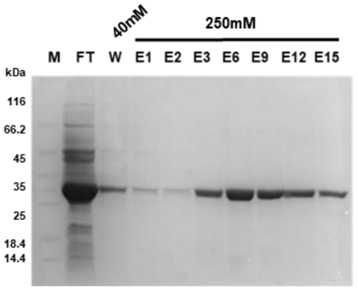

l. *Cb*FlaA7 co-expressed with *Cb*Maf

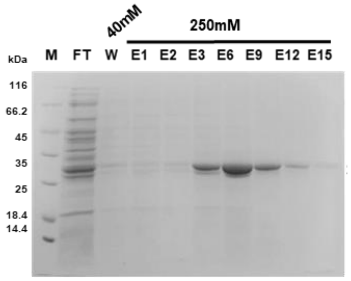

m. Singly expressed *Cb*FlaA11

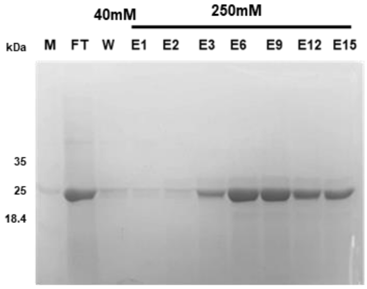

n. *Cb*FlaA11 co-expressed with *Cb*Maf

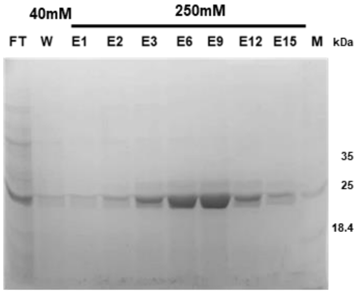

o. Singly expressed *Cb*FlaA4_A5

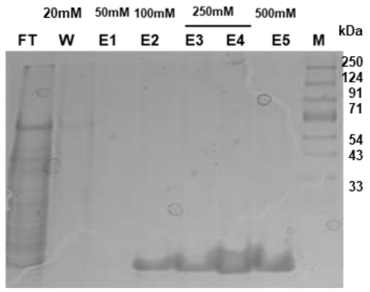

p. *Cb*FlaA4_A5 co-expressed with *Cb*Maf

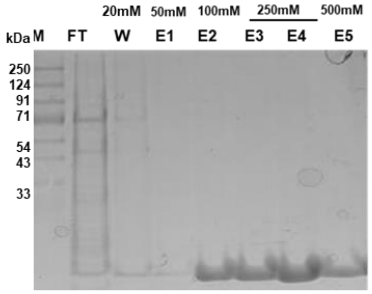

q. Singly expressed *Cb*FlaA3_A7

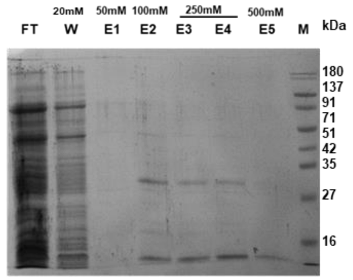

r. *Cb*FlaA3_A7 co-expressed with *Cb*Maf

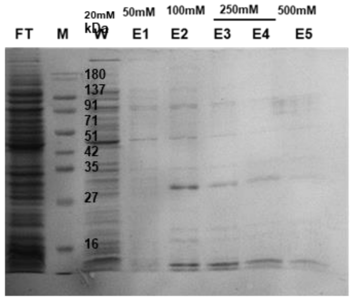

s. Singly expressed *Cb*FlaA4_A7

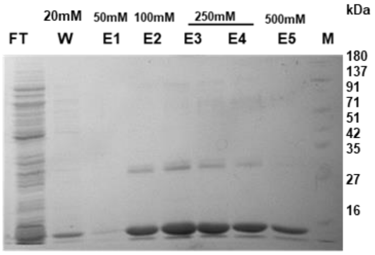

t. *Cb*FlaA4_A7 co-expressed with *Cb*Maf

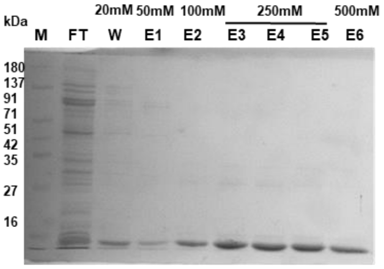

u. Singly expressed *Cb*FlaA7v

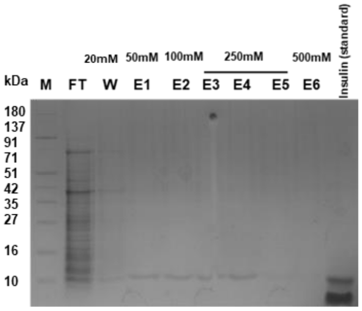

v. *Cb*FlaA7v co-expressed with *Cb*Maf

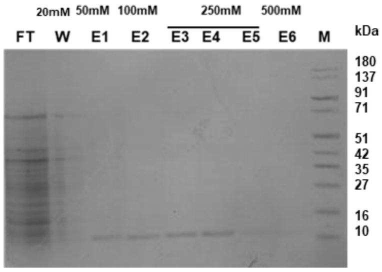

w. Singly expressed *Cb*FlaD0A7loop

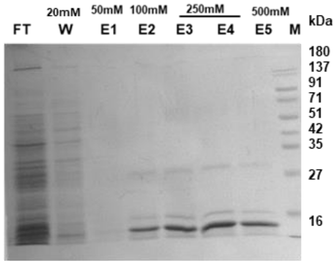

x. *Cb*FlaD0A7loop co-expressed with *Cb*Maf

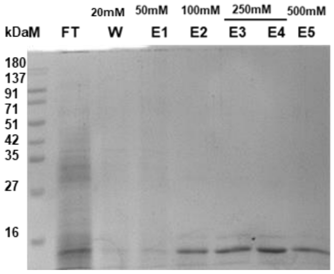

y. Singly expressed *Sr*NaFLD

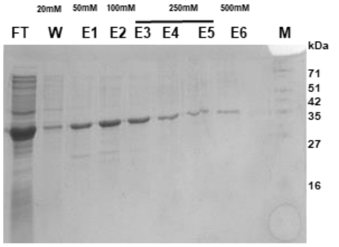

z. *Sr*NaFLD co-expressed with *Cb*Maf

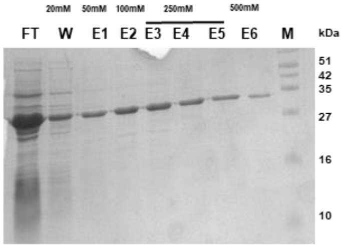

aa. Singly expressed *Cb*FlaA7v*Sr*NaFLD

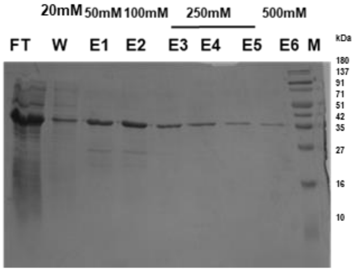

ab. *Cb*FlaA7v*Sr*NaFLD co-expressed with *Cb*Maf

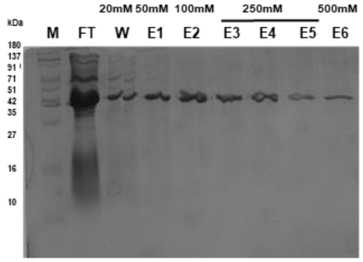

ac. Singly expressed *Sr*Na*Cb*FlaA1FLD

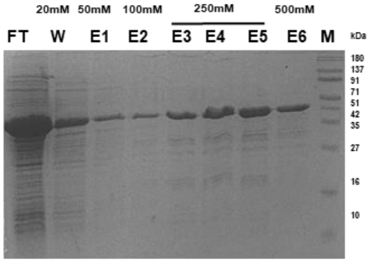

ad. *Sr*Na*Cb*FlaA1FLD co-expressed with *Cb*Maf

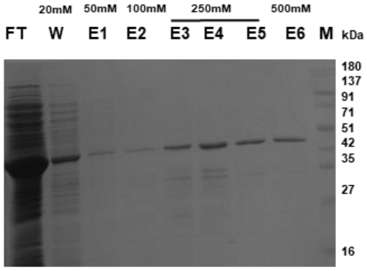

ae. Singly expressed *Cb*FlaA1*Sr*NaFLD

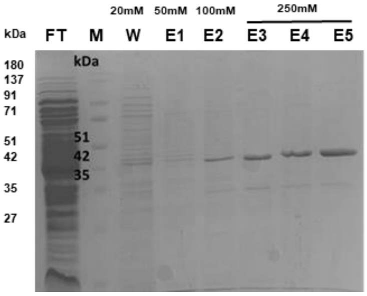

af. *Cb*FlaA1*Sr*NaFLD co-expressed with *Cb*Maf

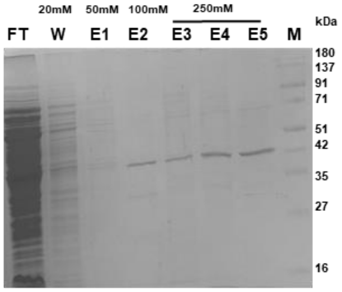

ag. **Singly expressed GKGAKS-*Sr*NaFLD**

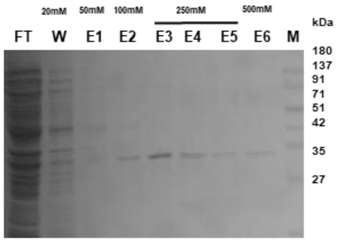

ah. **GKGAKS-*Sr*NaFLD co-expressed with *Cb*Maf**

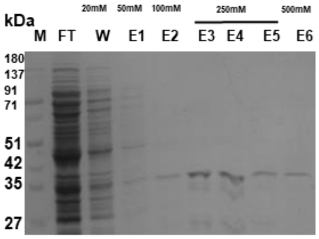

ai. **Singly expressed *Cb*Fla_A1helix_GKGAKS-*Sr*NaFLD**

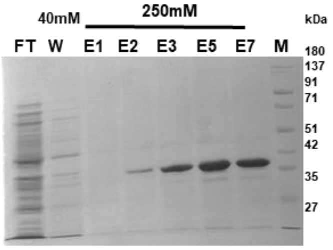

aj. ***Cb*Fla_A1helix_GKGAKS-*Sr*NaFLD co-expressed with *Cb*Maf**

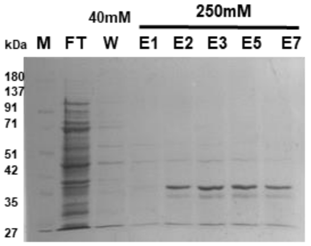

ak. ***Cb*Fla co-expressed with *Gk*Maf**

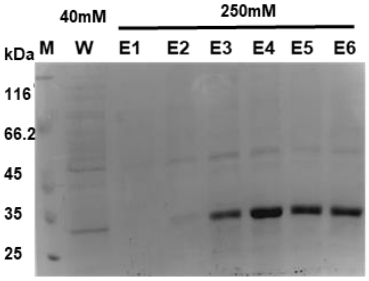

al. ***Gk*FlaA1 co-expressed with *Cb*Maf**

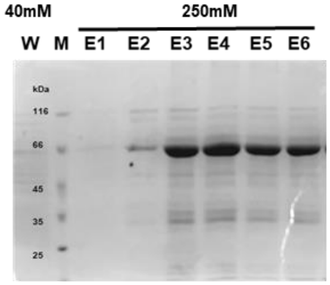

am. ***Gk*FlaA2 co-expressed with *Cb*Maf**

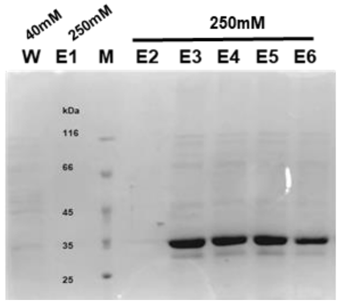

Supplementary Figure 1: Metal ion affinity chromatography based purification of flagellin, mini-flagellin constructs, and mini-flagellin chimeras, either singly expressed (**a, c, e, g, I, k, m,**

o, s, u, w, y, aa, ac, ae, ag, ai) or co-expressed with *Cb*Maf (**b, d, f, h, j, l, n, p, r, t, v, x, z, ab, ad, af, ah, aj, al, am**) or *Gk*Maf (**ak**). FT: column flow through; W: wash; E: eluate; M: molecular mass marker. The concentrations indicated above the gel images refer to the concentrations of imidazole used in column washes and eluates.

Supplementary Figure 2

a. *Cb*Fla-*Cb*Maf

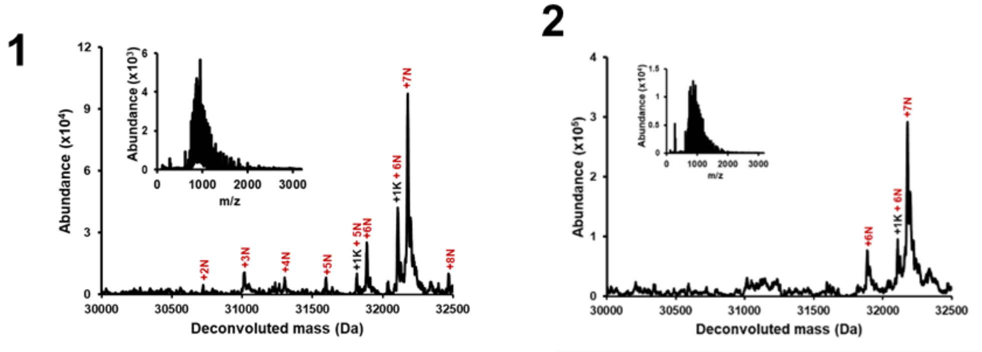

b. *Cb*FlaA1-*Cb*Maf

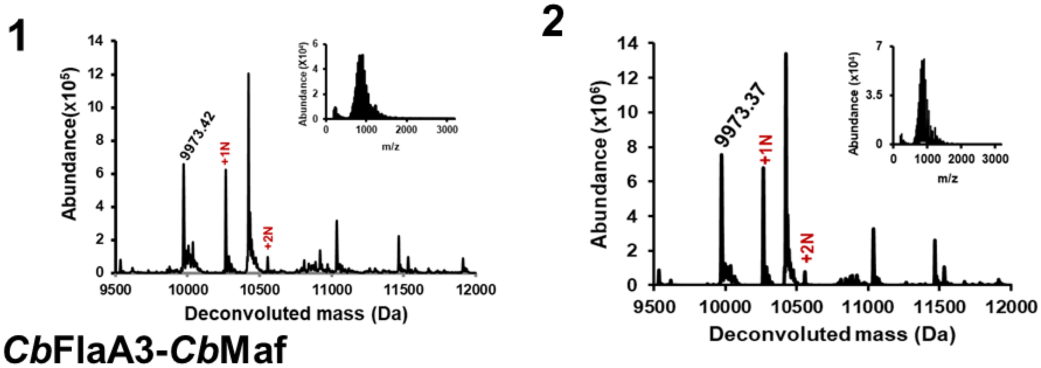

c. *Cb*FlaA3-*Cb*Maf

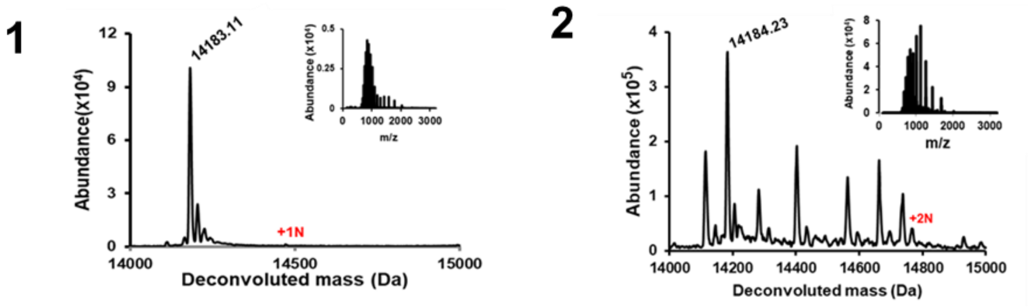

d. *Cb*FlaA4-*Cb*Maf

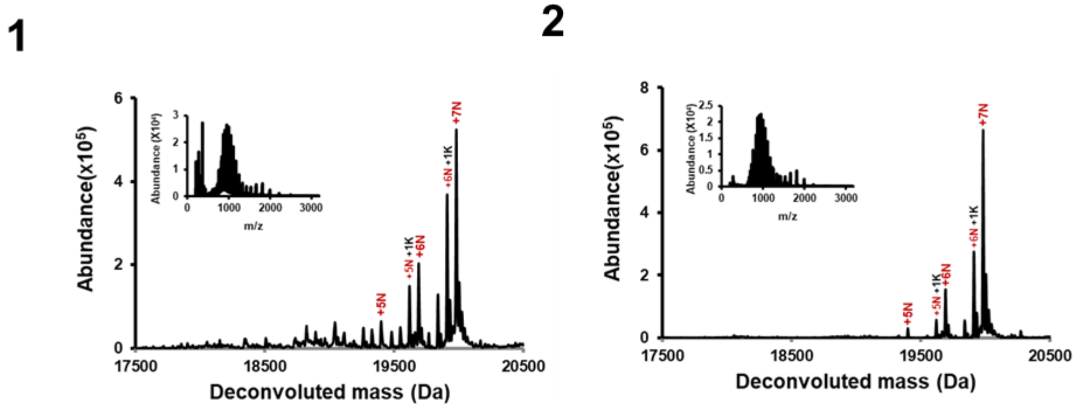

e. *Cb*FlaA5-*Cb*Maf

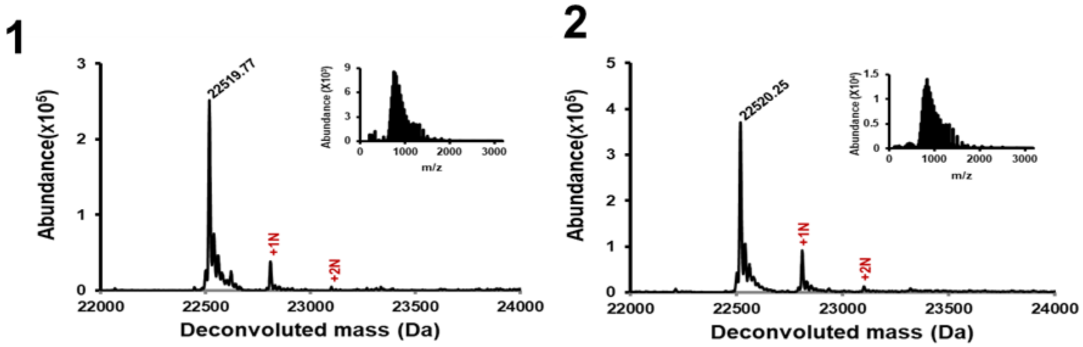

f. *Cb*FlaA7-*Cb*Maf

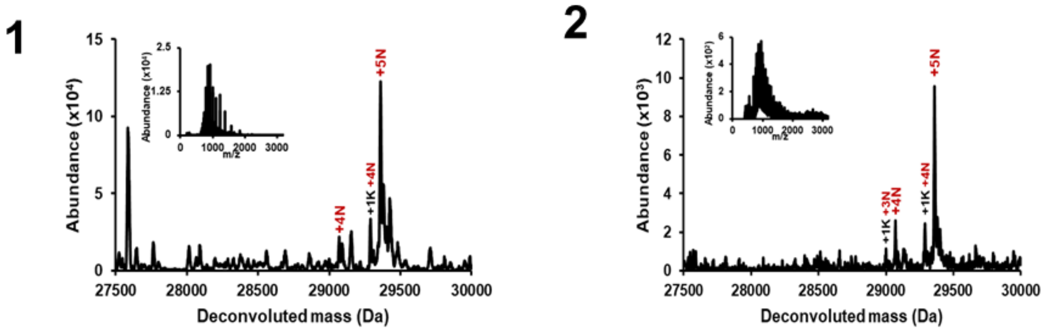

g. *Cb*FlaA11-*Cb*Maf

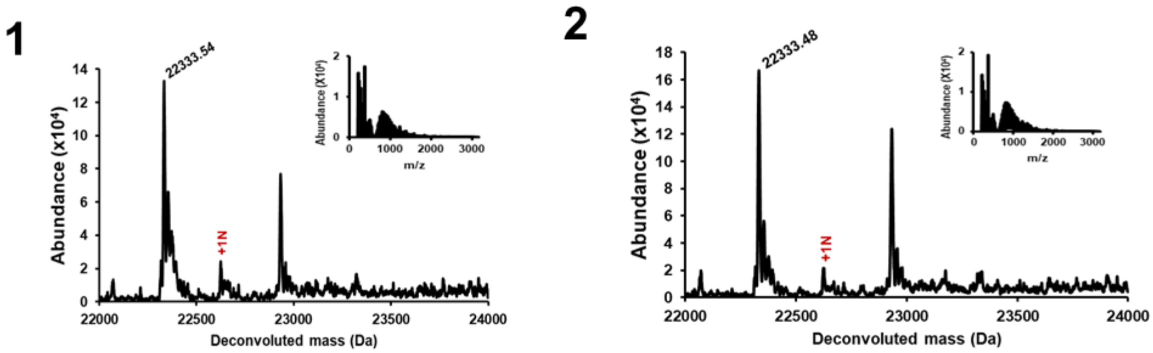

h. *Cb*FlaA4_A5-*Cb*Maf

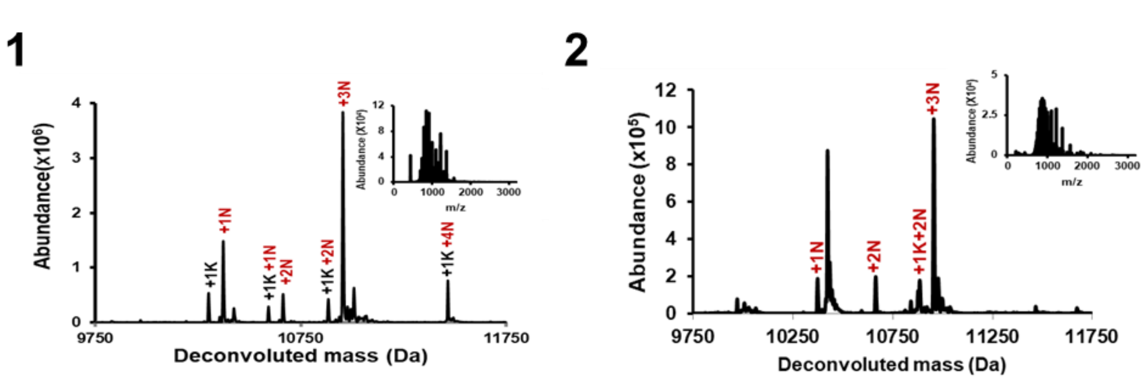

i. *Cb*FlaA3_A7-*Cb*Maf

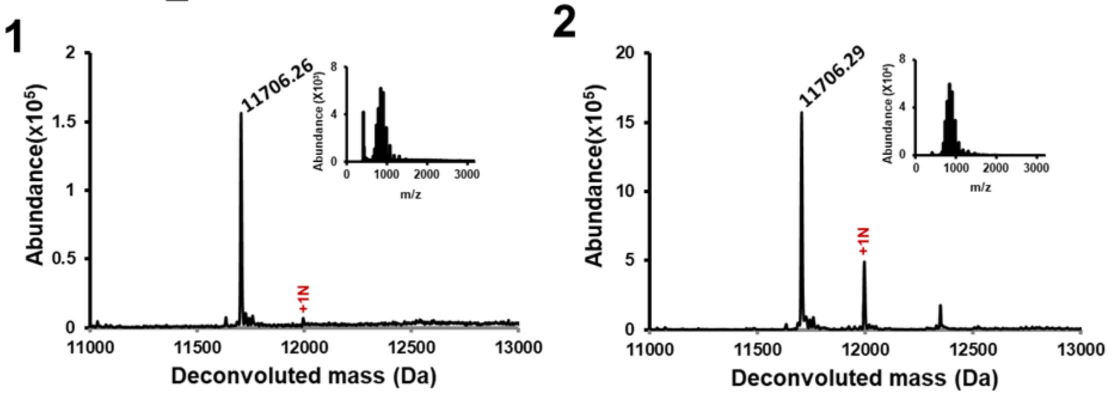

j. *Cb*FlaA4_A7-*Cb*Maf

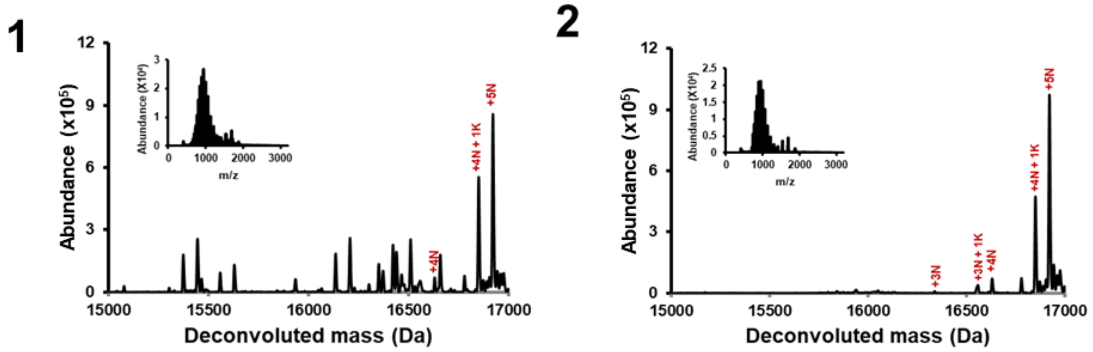

k. *Cb*FlaA7v-*Cb*Maf

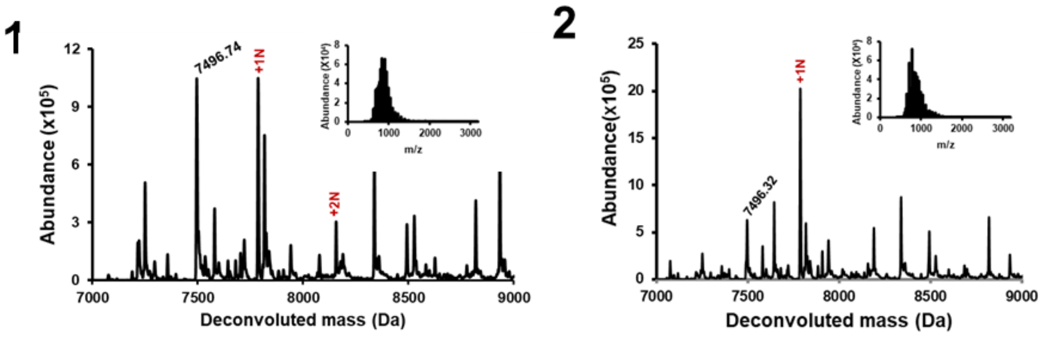

l. *Cb*FlaA7v*Sr*NaFLD-*Cb*Maf

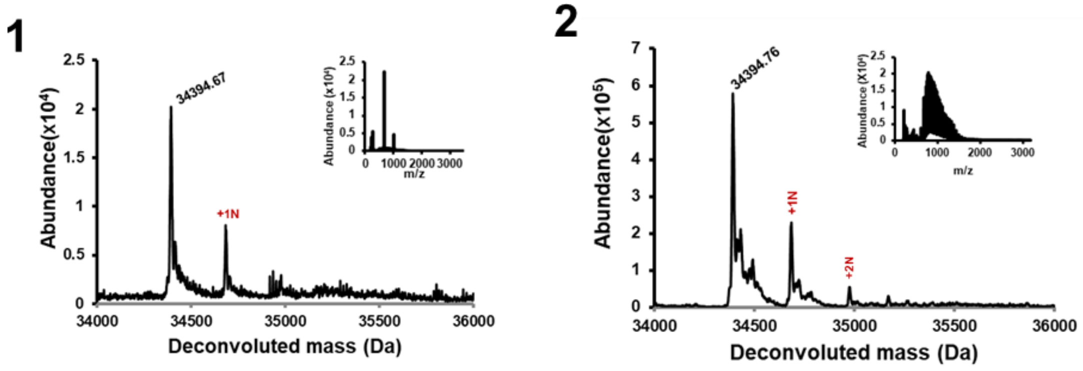

m. *Sr*Na_*Cb*FlaA1_*Sr*FLD-CbMaf

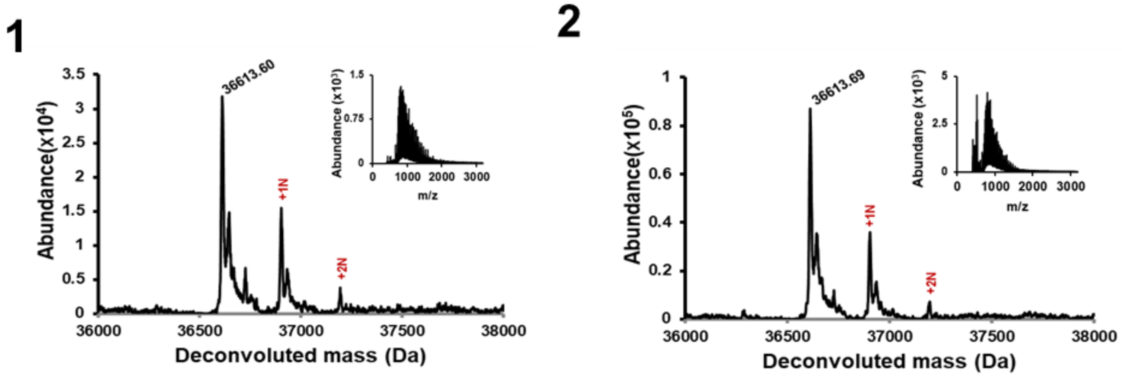

n. *Cb*FlaA1*Sr*NaFLD-*Cb*Maf

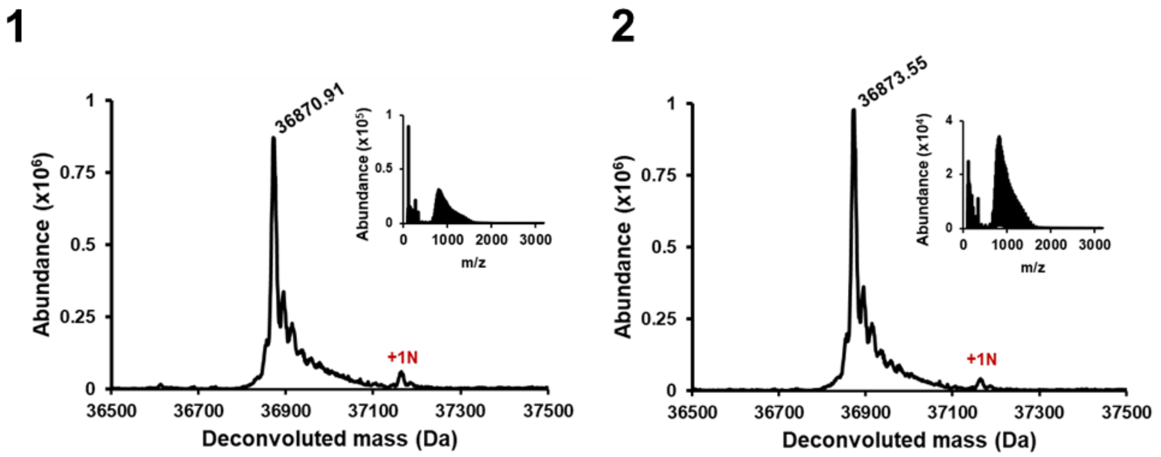

Supplementary figure 2: Intact mass measurements of two additional biological replicates of *Cb*Fla (**a**), *Cb*FlaA1 (**b**), *Cb*FlaA3 (**c**), *Cb*FlaA4 (**d**), *Cb*FlaA5 (**e**), *Cb*FlaA7 (**f**), *Cb*FlaA11 (**g**), *Cb*FlaA4_A5 (**h**), *Cb*FlaA3_A7 (**i**), *Cb*FlaA4_A7 (**j**), *Cb*FlaA7v (**k**), *Cb*FlaA7v*Sr*NaFLD (**l**), *Sr*Na*Cb*FlaA1*Sr*FLD (**m**), and *Cb*FlaA1*Sr*NaFLD (**n**), each co-expressed with *Cb*Maf in EV136 cells. Insets in each show the ionization spectra of the flagellin *constructs.* *Cb*FlaA constructs were expressed on three different occasions with *Cb*Maf in *E*. *coli* EV136, purified by Ni-NTA metal ion affinity chromatography, and subjected to intact mass analysis.

Supplementary figure 3

a1. Sequence coverage (shown by underlining) of trypsin digested *Cb*Fla.

```
1    MAIINHNLNA MNAHRQMAIN TGSNGKAIEK LSSGLRINRA GDDAAGLAIS
51   EKMRGQIRGL NQASRNSQDS ISLIQTAEGA LNETHSILQR MRELAVQSAN
101  DTNVKVDRDN LQKEVAELQN EINRIASQTQ FNTKNLLNGS AKSMVFQIGA
151  NKDQVMELTI AGMGTSALKV GSINIGSGKG AKSISGMIET IDNAINTVSG
201  ERAKLGANQN RLEHTIANLD NSAENLQAAE SRIRDVDMAK EMMNFTKTNI
251  LTQAAQAMLA QANQAPQGVL QLLRHHHHHH
```

a2. **Sequence coverage (shown by underlining) of trypsin digested *Cb*Fla co-expressed with *Cb*Maf. Modifications (predicted by Modscore>19) with Neu5Ac and KDO are indicated by @ and #, respectively.**

```
1    MAIINHNLNA MNAHRQMAIN TGSNGKAIEK LSSGLRINRA GDDAAGLAIS
51   EKMRGQIRGL NQASRNSQDS ISLIQTAEGA LNETHSILQR MRELAVQSAN
101  DTNVKVDRDN LQKEVAELQN EINRIASQTQ FNTKNLLNGS AKSMVFQIGA
151  NKDQVMELTI AGMGTSALKV GSINIGSGKG AKSISGMIET IDNAINTVSG
201  ERAKLGANQN RLEHTIANLD NSAENLQAAE SRIRDVDMAK EMMNFTKTNI
251  LTQAAQAMLA QANQAPQGVL QLLRHHHHHH
```

a3. **Sequence coverage (shown by underlining) of trypsin digested *Cb*Fla co-expressed with *Cb*Maf with confidently assigned glycosites (validated by at least one MS/MS spectrum containing at least one glycan-modified fragment ion). Modifications with Neu5Ac and KDO are indicated by @ and #, respectively.**

```
1    MAIINHNLNA MNAHRQMAIN TGSNGKAIEK LSSGLRINRA GDDAAGLAIS
51   EKMRGQIRGL NQASRNSQDS ISLIQTAEGA LNETHSILQR MRELAVQSAN
101  DTNVKVDRDN LQKEVAELQN EINRIASQTQ FNTKNLLNGS AKSMVFQIGA
151  NKDQVMELTI AGMGTSALKV GSINIGSGKG AKSISGMIET IDNAINTVSG
201  ERAKLGANQN RLEHTIANLD NSAENLQAAE SRIRDVDMAK EMMNFTKTNI
251  LTQAAQAMLA QANQAPQGVL QLLRHHHHHH
```

a4. **Domain architecture of full length *Cb*Fla. ND1a and CD1 have been partitioned into ND1a1, ND1a2, and ND1a3, and CD1_1, CD1_2 and CD1_3, respectively and colored differently. Glycosites with Neu5Ac and KDO are indicated by symbols (♦) and (⬡), respectively.**

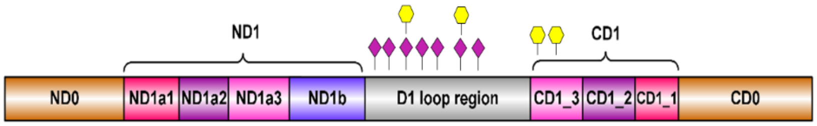

a5. MS/MS spectrum for a trypsin digested peptide from *Cb*Fla co-expressed with *Cb*Maf

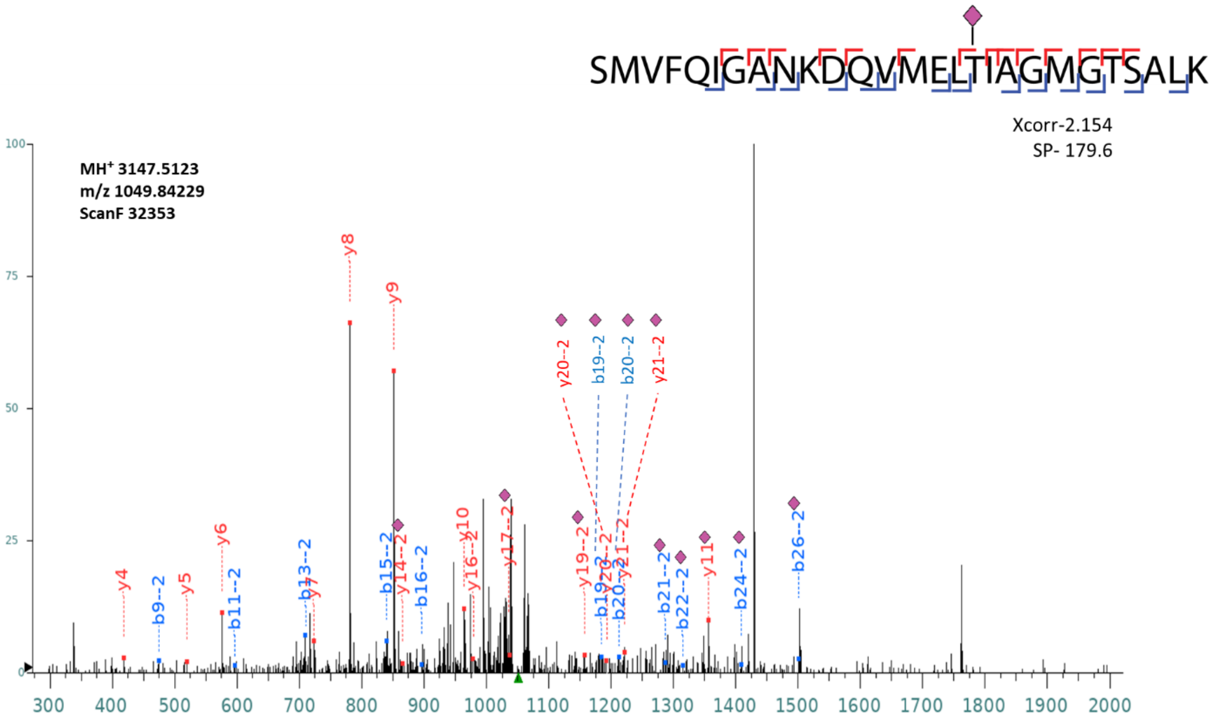

a6. MS/MS spectrum for a trypsin digested peptide from *Cb*Fla co-expressed with *Cb*Maf

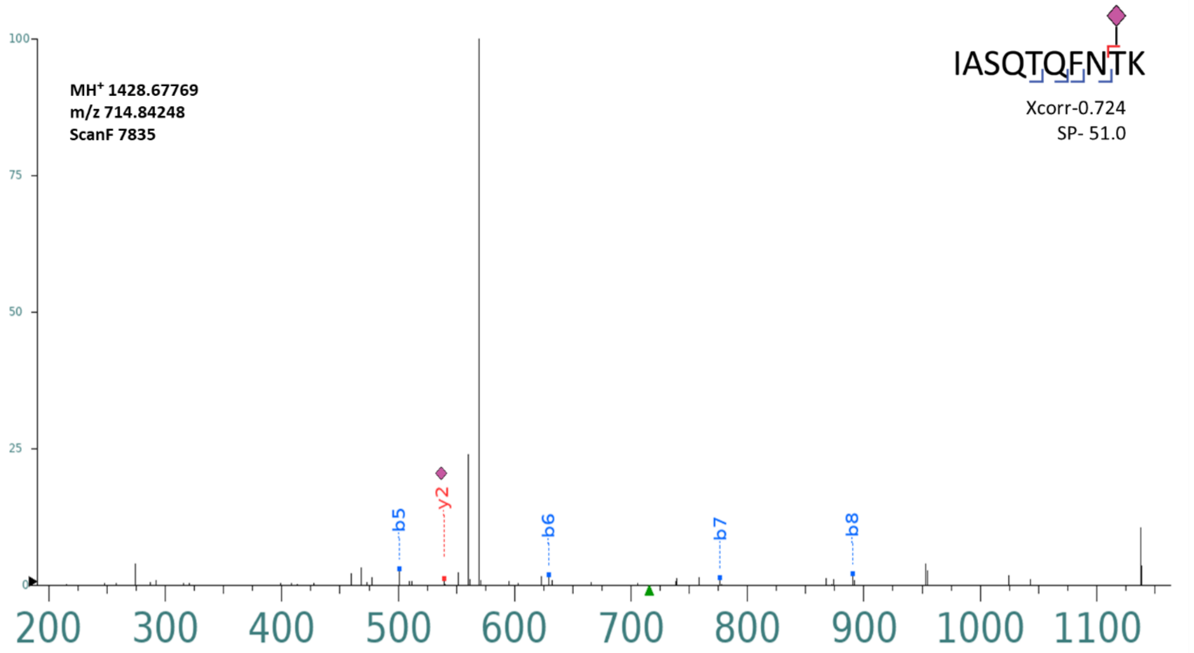

a7. MS/MS spectrum for a trypsin digested peptide from *Cb*Fla co-expressed with *Cb*Maf

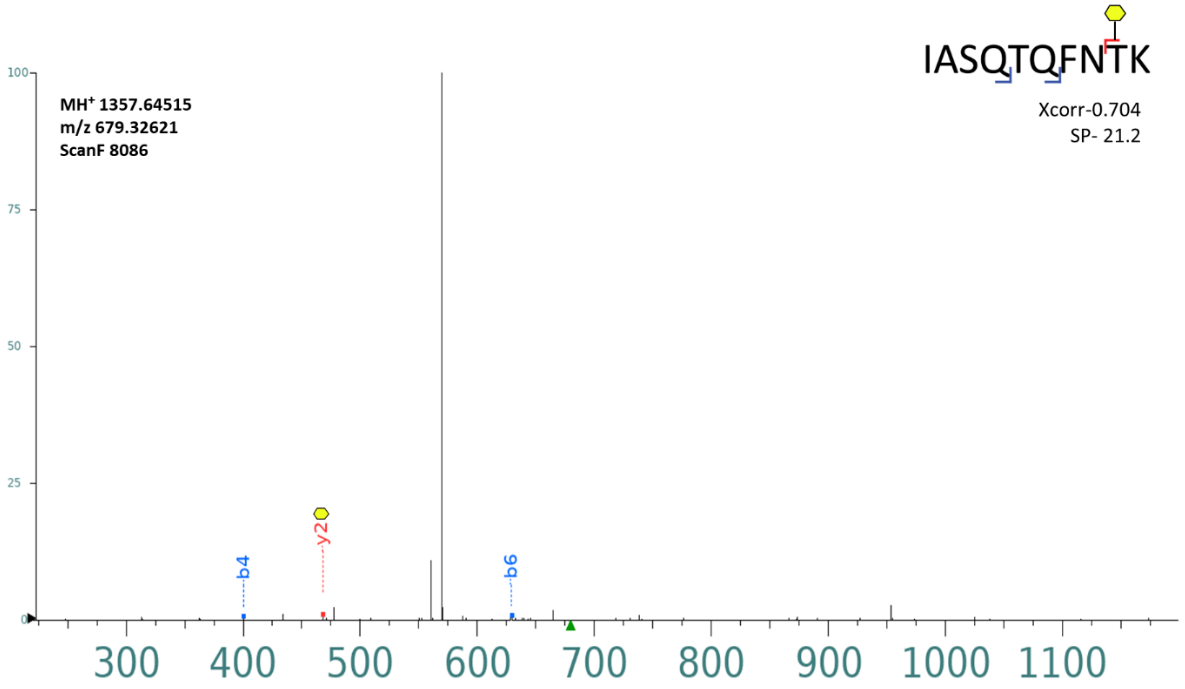

a8. MS/MS spectrum for a trypsin digested peptide from *Cb*Fla co-expressed with *Cb*Maf

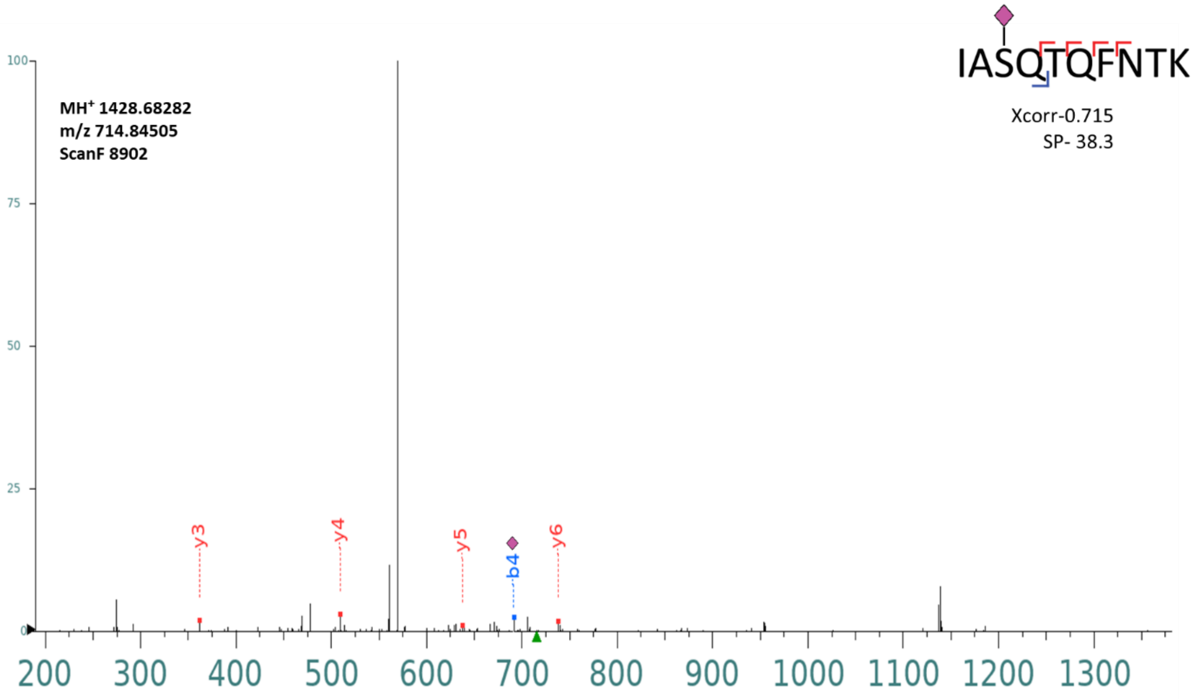

a9. MS/MS spectrum for a trypsin digested peptide from *Cb*Fla co-expressed with *Cb*Maf

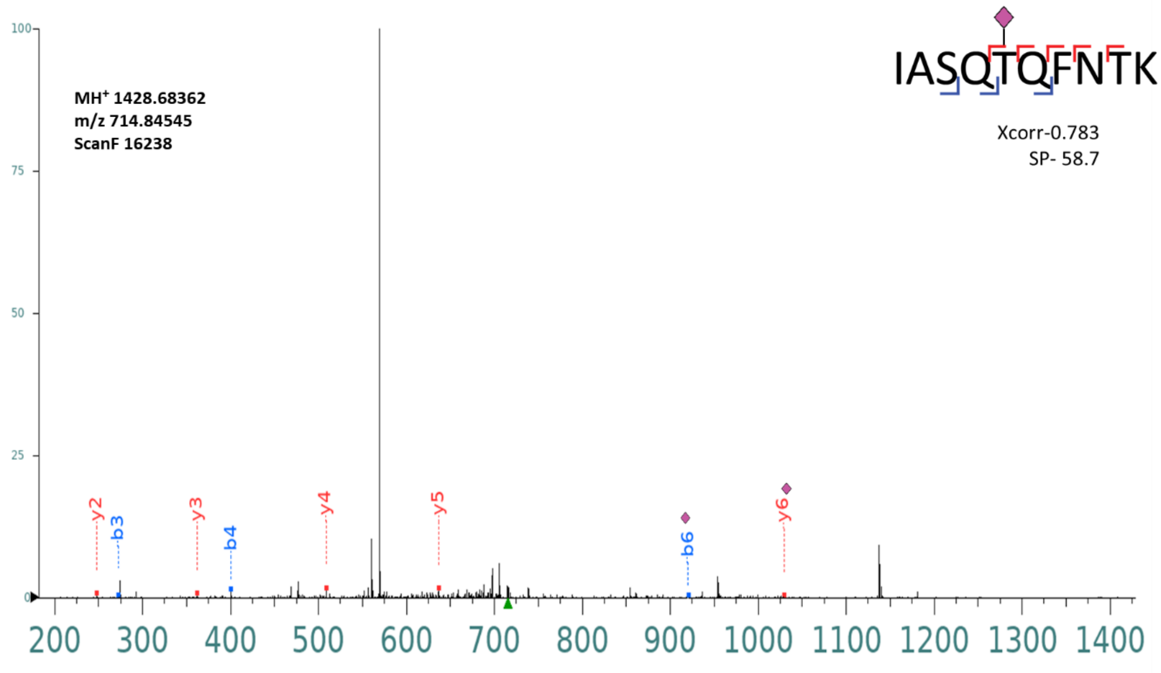

a10. MS/MS spectrum for a GluC digested peptide from *Cb*Fla co-expressed with *Cb*Maf

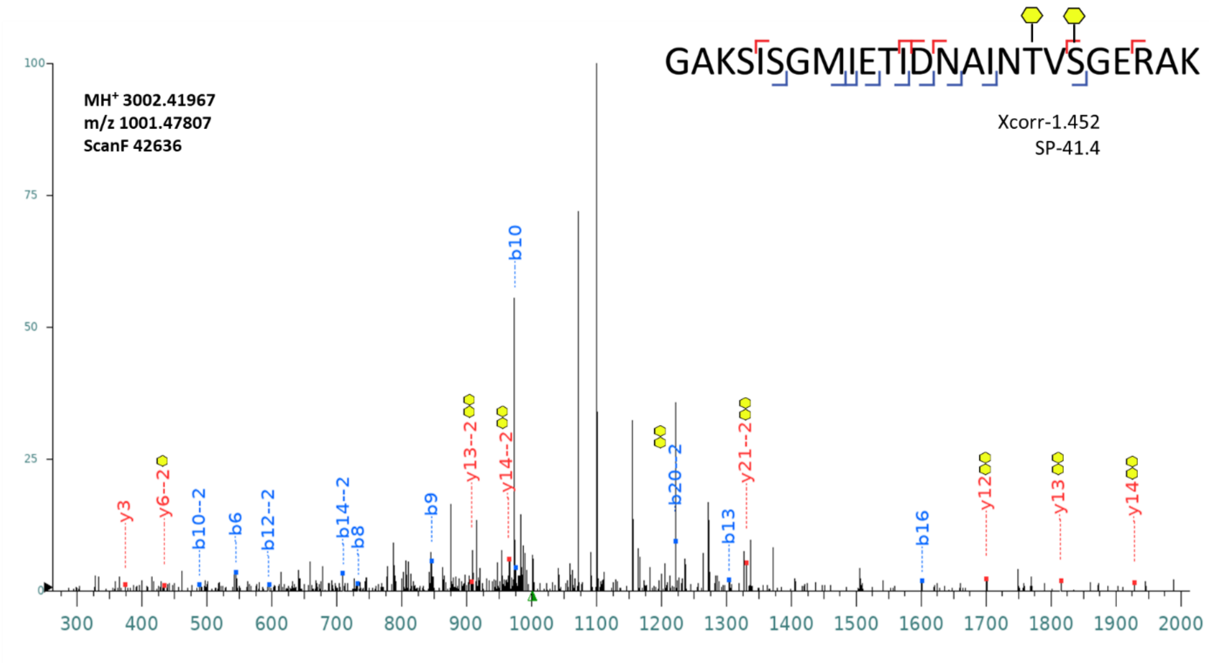

a11. MS/MS spectrum for a trypsin digested peptide from *Cb*Fla co-expressed with *Cb*Maf

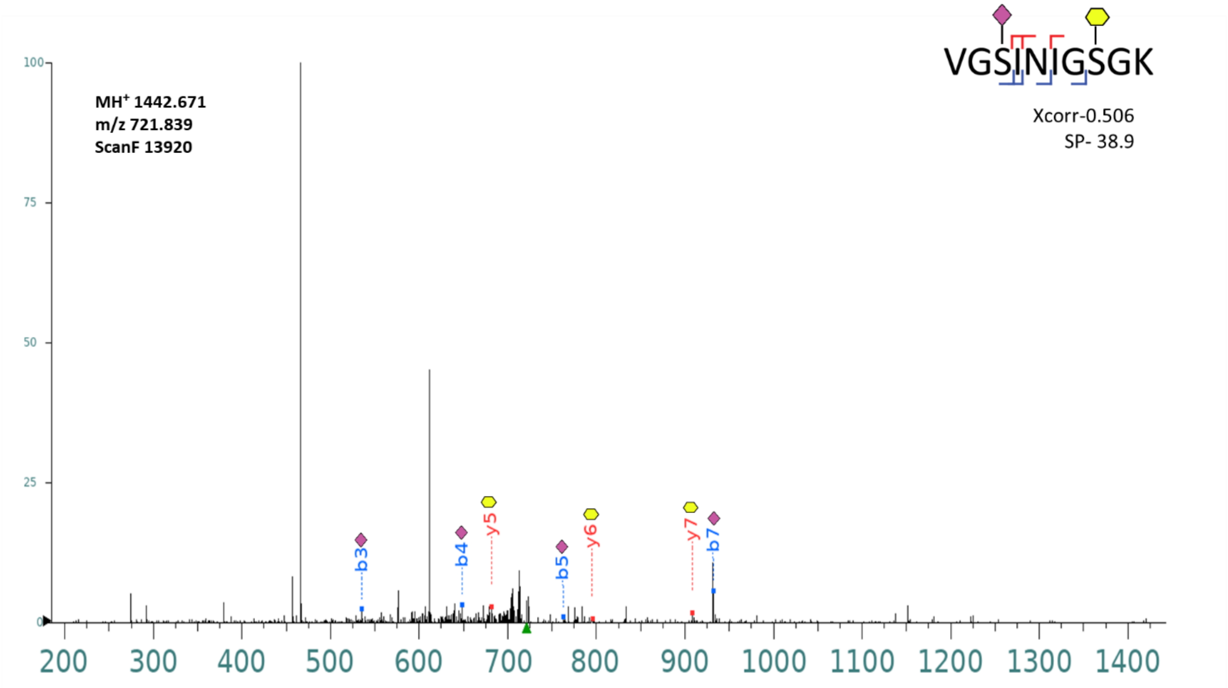

a12. MS/MS spectrum for a trypsin digested peptide from *Cb*Fla co-expressed with *Cb*Maf

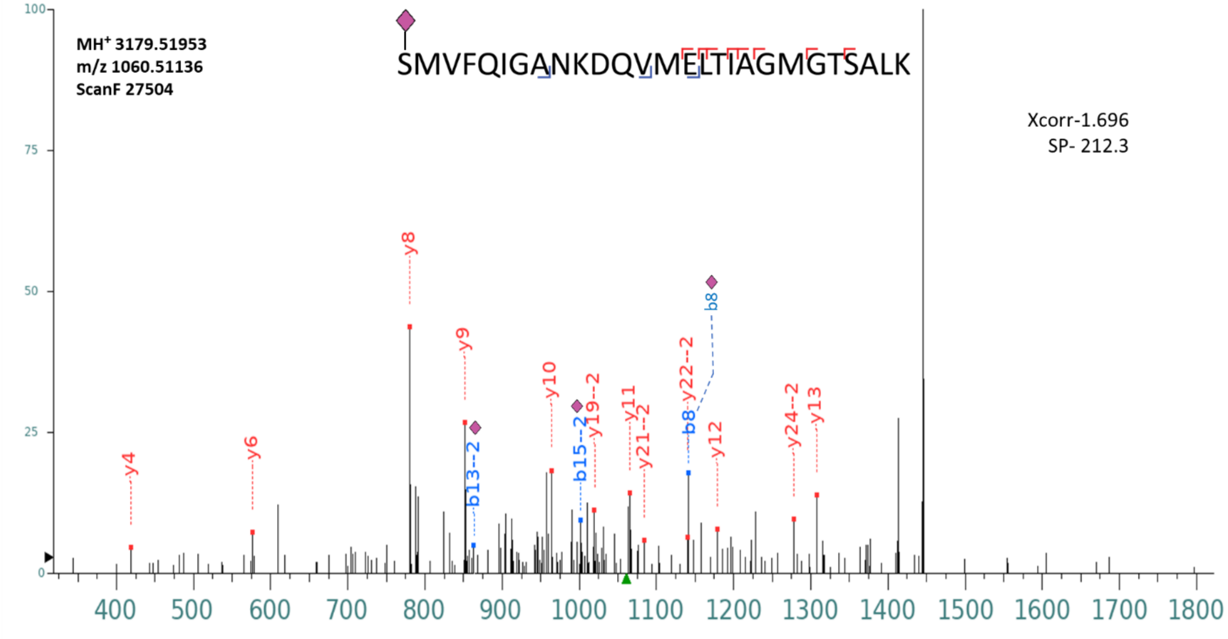

a13. MS/MS spectrum for a trypsin digested peptide from *Cb*Fla co-expressed with *Cb*Maf

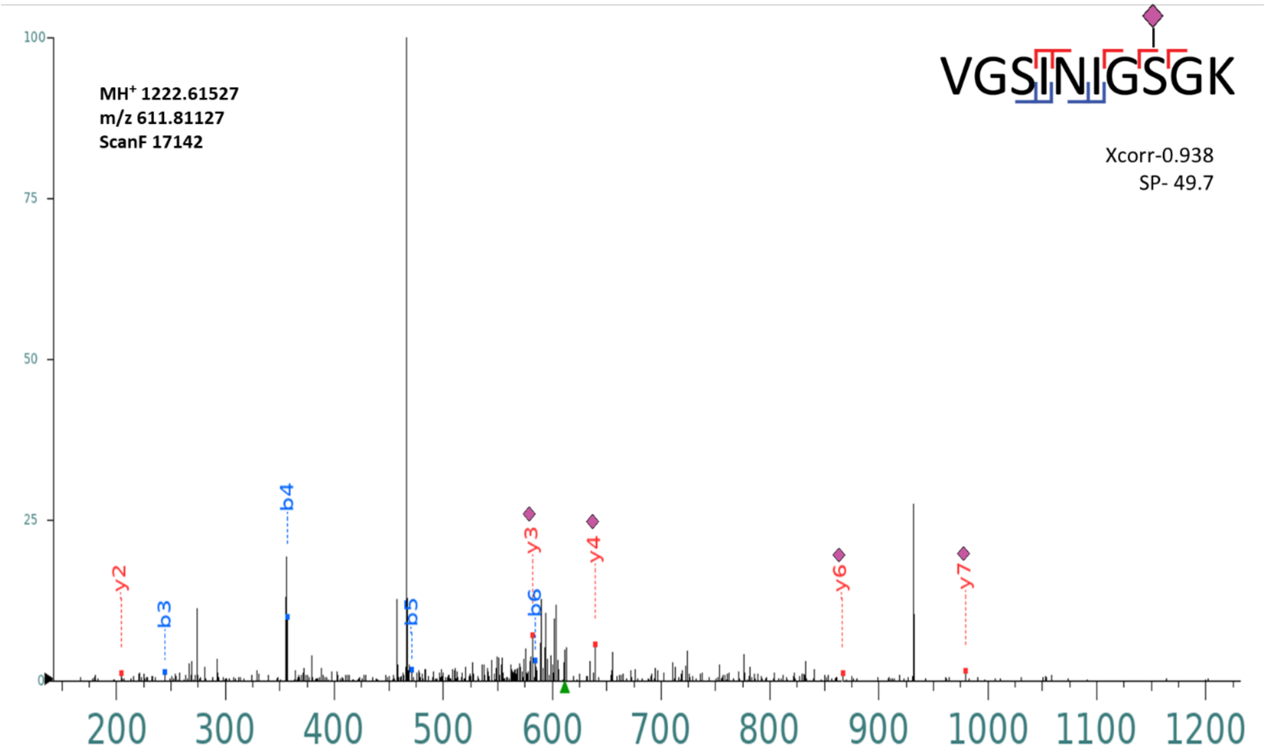

b1. Sequence coverage (shown by underlining) of trypsin digested *Cb*FlaA1.

```
1   MADTNVKVDR DNLQKEVAEL QNEINRIASQ TQFNTKNLLN GSAKSMVFQI
51  GANKDQVMEL TIAGMGTSAL KVGSINIGSG KGAKSLEHHH HHH
```

b2. Sequence coverage (shown by underlining) of trypsin digested *Cb*FlaA1 co-expressed with *Cb*Maf.

```
1   MADTNVKVDR DNLQKEVAEL QNEINRIASQ TQFNTKNLLN GSAKSMVFQI
51  GANKDQVMEL TIAGMGTSAL KVGSINIGSG KGAKSLEHHH HHH
```

c1. Sequence coverage (shown by underlining) of trypsin digested *Cb*FlaA3.

```
1   MAALNETHSI LQRMRELAVQ SANDTNVKVD RDNLQKEVAE LQNEINRIAS
51  QTQFNTKNLL NGSAKSMVFQ IGANKDQVME LTIAGMGTSA LKVGSINIGS
101 GKGAKSISGM IETIDNAINT VSGERAKLGA NQNRLEHTIA NLLEHHHHHH
```

c2. Sequence coverage (shown by underlining) of trypsin digested *Cb*FlaA3 co-expressed with *Cb*Maf. Modifications (predicted by Modscore>19) with Neu5Ac and KDO are indicated by @ and #, respectively. All glycosites are confidently assigned (validated by at least one MS/MS spectrum containing at least one glycan-modified fragment ion).

```
                                                      @      @
1   MAALNETHSI LQRMRELAVQ SANDTNVKVD RDNLQKEVAE LQNEINRIAS
51  QTQFNTKNLL NGSAKSMVFQ IGANKDQVME LTIAGMGTSA LKVGSINIGS
101 GKGAKSISGM IETIDNAINT VSGERAKLGA NQNRLEHTIA NLLEHHHHHH
```

c3. Domain architecture of full length *Cb*FlaA3. Glycosites with Neu5Ac is indicated by symbol (♦).

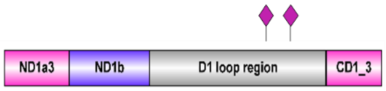

c4. MS/MS spectrum for a trypsin digested peptide from *Cb*FlaA3 co-expressed with *Cb*Maf

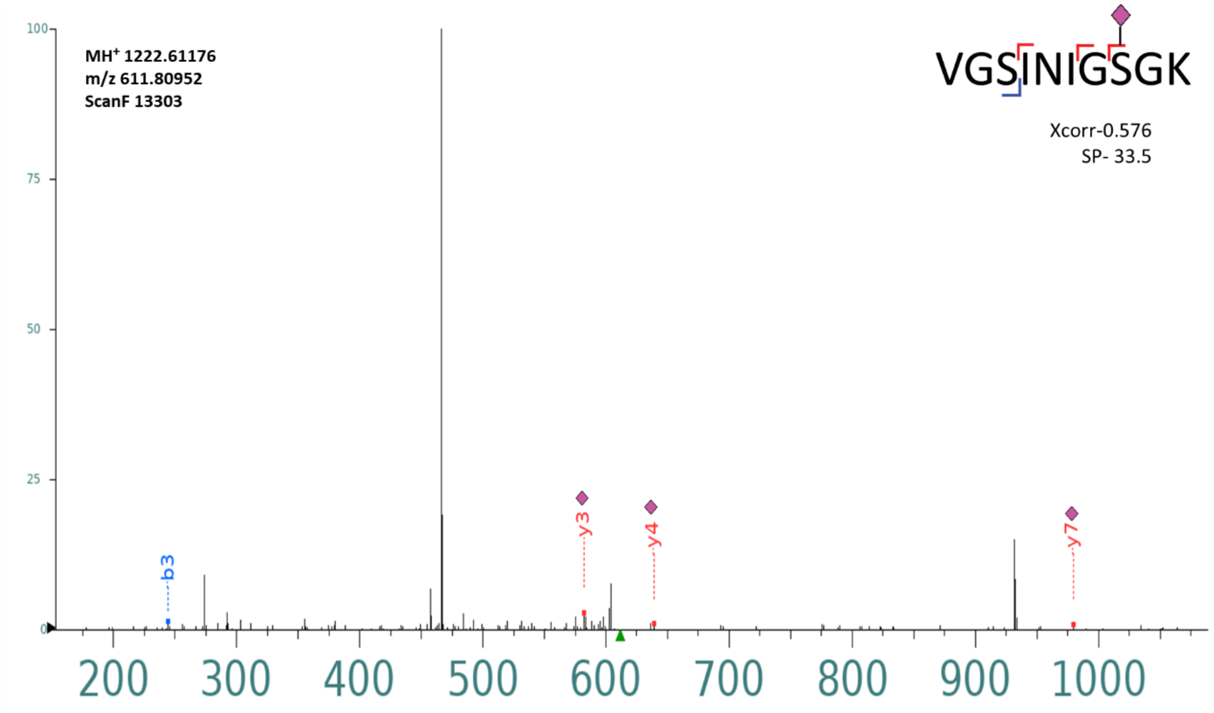

c5. MS/MS spectrum for a trypsin digested peptide from *Cb*FlaA3 co-expressed with *Cb*Maf

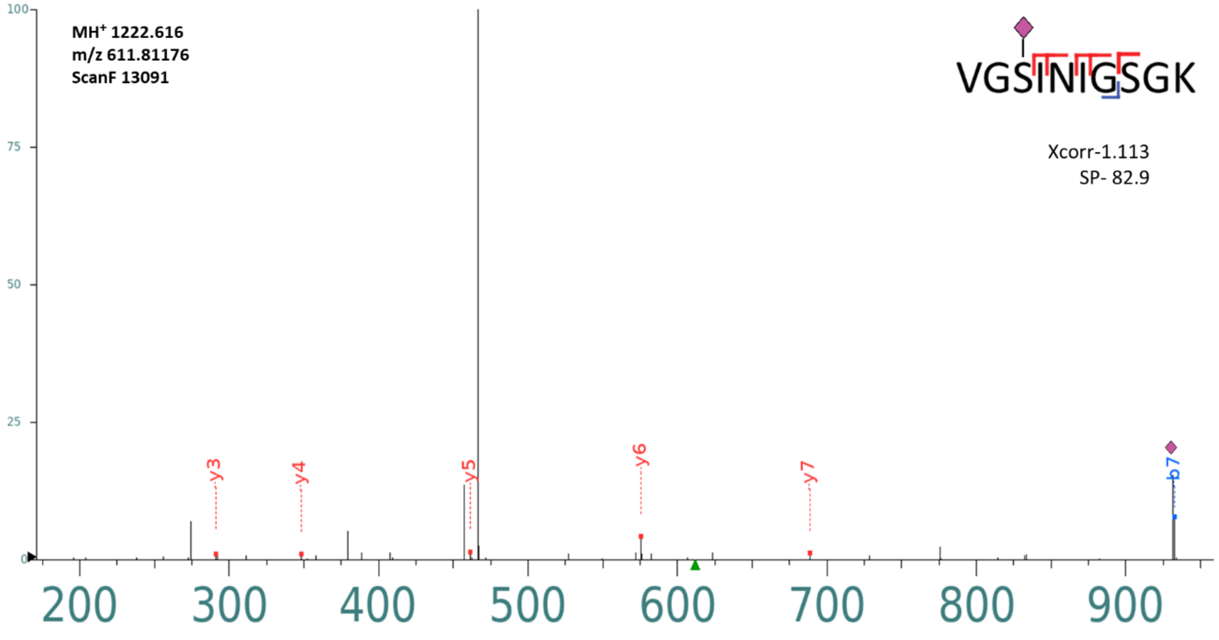

d1. Sequence coverage (shown by underlining) of trypsin digested *Cb*FlaA4.

```
1    MAASRNSQDS ISLIQTAEGA LNETHSILQR MRELAVQSAN DTNVKVDRDN
51   LQKEVAELQN EINRIASQTQ FNTKNLLNGS AKSMVFQIGA NKDQVMELTI
101  AGMGTSALKV GSINIGSGKG AKSISGMIET IDNAINTVSG ERAKLGANQN
151  RLEHTIANLL EHHHHHH
```

d2. Sequence coverage (shown by underlining) of trypsin digested *Cb*FlaA4 co-expressed with *Cb*Maf. Modifications (predicted by Modscore>19) with Neu5Ac and KDO are indicated by @ and #, respectively.

```
1    MAASRNSQDS ISLIQTAEGA LNETHSILQR MRELAVQSAN DTNVKVDRDN
51   LQKEVAELQN EINRIASQTQ FNTKNLLNGS AKSMVFQIGA NKDQVMELTI
101  AGMGTSALKV GSINIGSGKG AKSISGMIET IDNAINTVSG ERAKLGANQN
151  RLEHTIANLL EHHHHHH
```

d3. Sequence coverage (shown by underlining) of trypsin digested *Cb*FlaA4 co-expressed with *Cb*Maf with confidently assigned glycosites (validated by at least one MS/MS spectrum containing at least one glycan-modified fragment ion). Modifications with Neu5Ac and KDO are indicated by @ and #, respectively.

```
1    MAASRNSQDS ISLIQTAEGA LNETHSILQR MRELAVQSAN DTNVKVDRDN
51   LQKEVAELQN EINRIASQTQ FNTKNLLNGS AKSMVFQIGA NKDQVMELTI
101  AGMGTSALKV GSINIGSGKG AKSISGMIET IDNAINTVSG ERAKLGANQN
151  RLEHTIANLL EHHHHHH
```

d4. Domain architecture of full length *Cb*FlaA4. Glycosites with Neu5Ac and KDO are indicated by symbols ( ) and ( ), respectively.

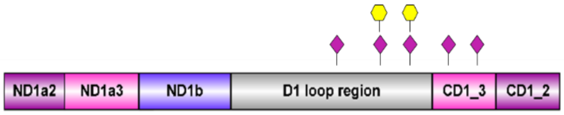

d5. MS/MS spectrum for a trypsin digested peptide from *Cb*FlaA4 co-expressed with *Cb*Maf

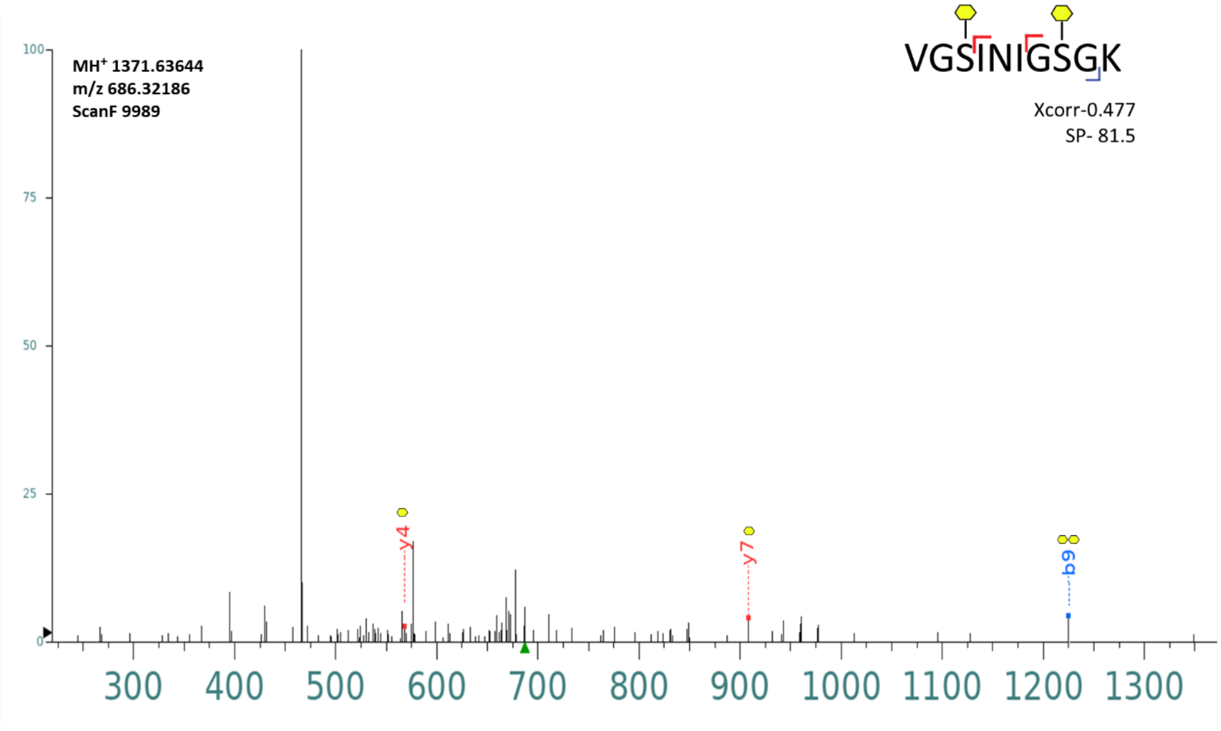

d6. MS/MS spectrum for a trypsin digested peptide from *Cb*FlaA4 co-expressed with *Cb*Maf

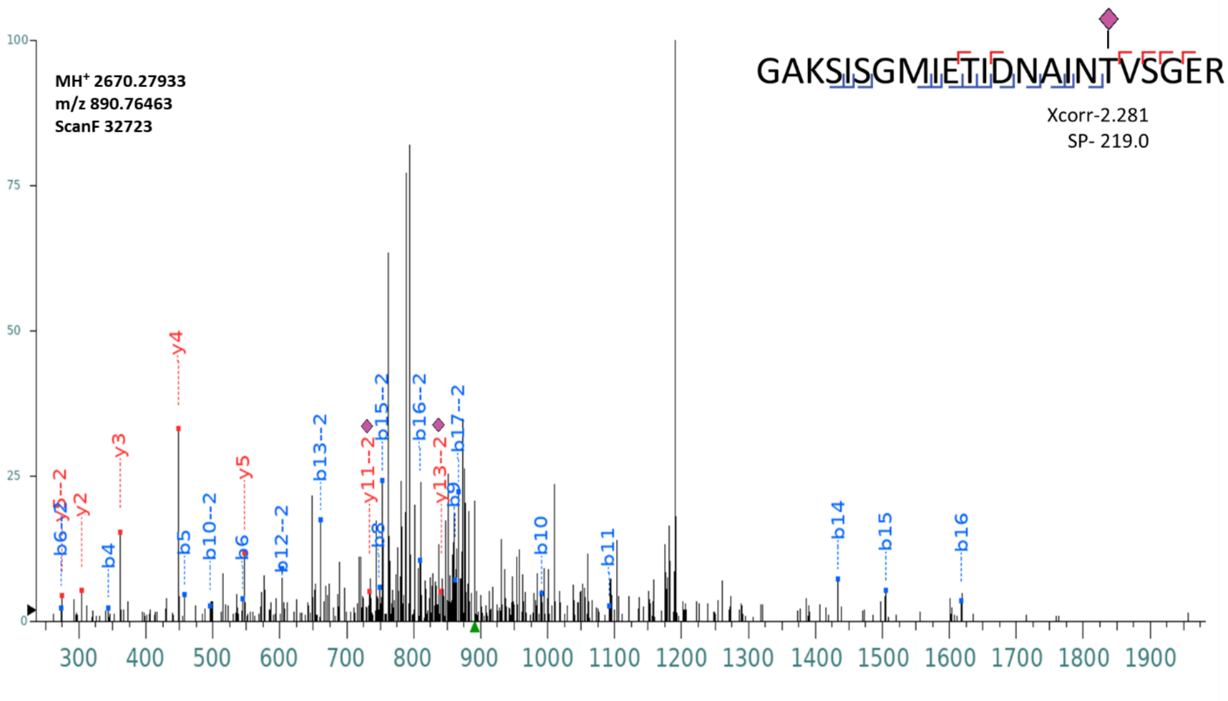

d7. MS/MS spectrum for a trypsin digested peptide from *Cb*FlaA4 co-expressed with *Cb*Maf

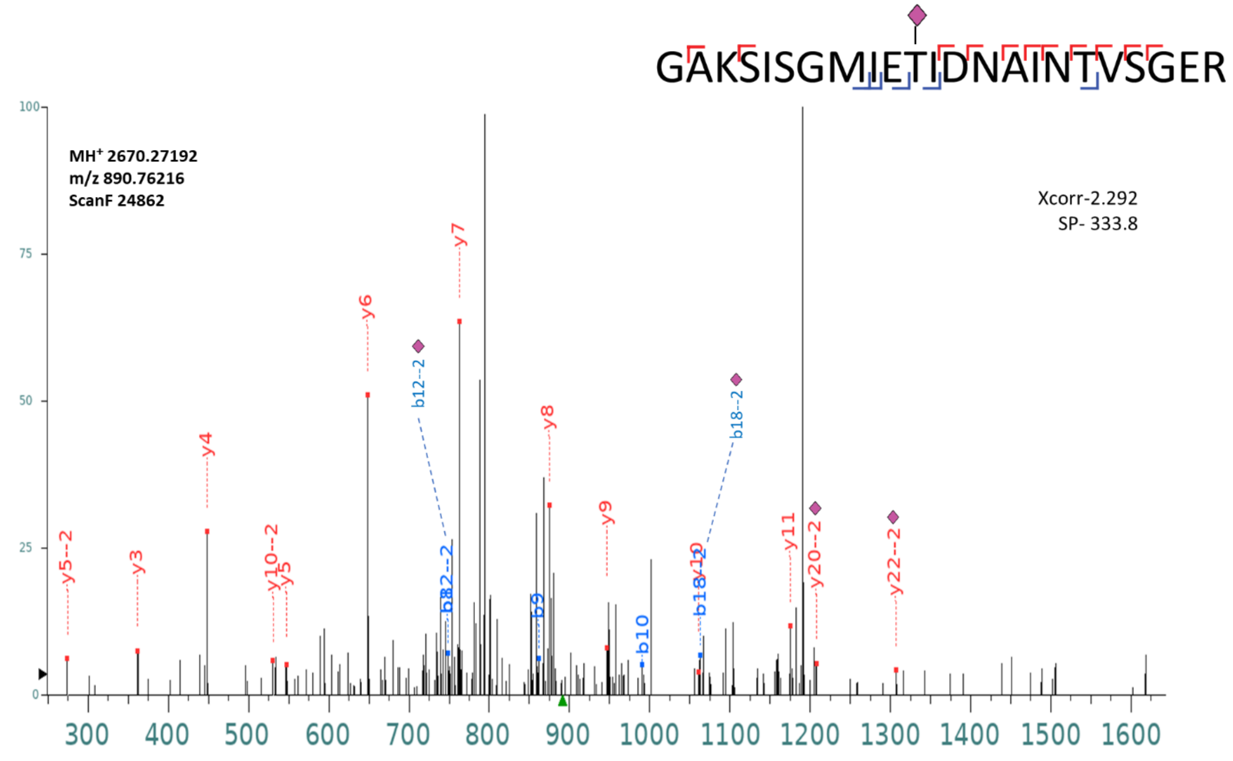

d8. MS/MS spectrum for a trypsin digested peptide from *Cb*FlaA4 co-expressed with *Cb*Maf

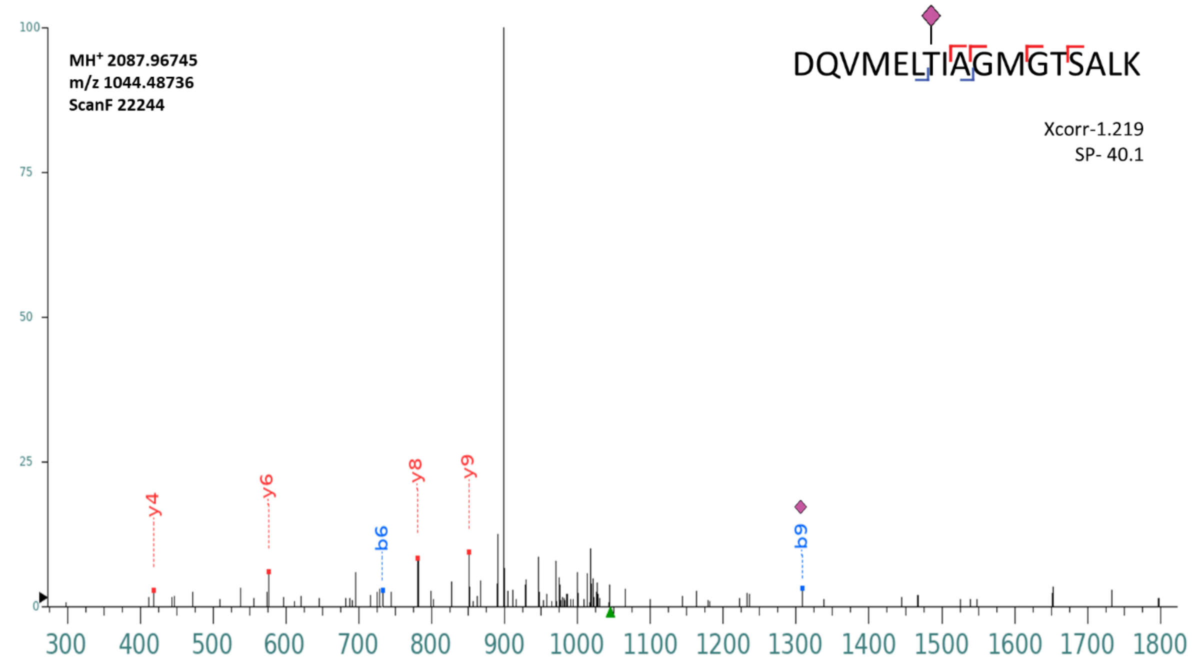

d9. MS/MS spectrum for a trypsin digested peptide from *Cb*FlaA4 co-expressed with *Cb*Maf

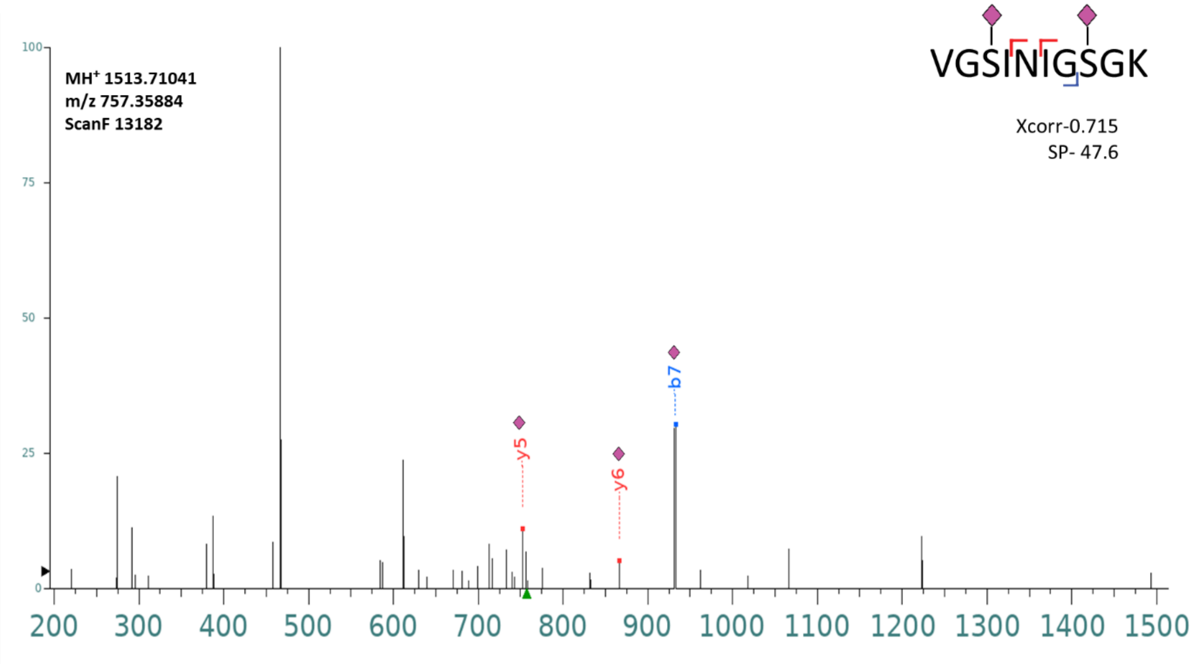

e1. Sequence coverage (shown by underlining) of trypsin digested *Cb*FlaA5.

```
1    MAIINHNLNA MNAHRQMAIN TGSNGKAIEK LSSGLRINRA GDDAAGLAIS
51   EKMRGQIRGL NQASRNSQDS ISLIQTAEGA LNETHSILQR MRELAVQSAN
101  DTNVGKGAKS ISGMIETIDN AINTVSGERA KLGANQNRLE HTIANLDNSA
151  ENLQAAESRI RDVDMAKEMM NFTKTNILTQ AAQAMLAQAN QAPQGVLQLL
201  RHHHHHH
```

e2. Sequence coverage (shown by underlining) of trypsin digested *Cb*FlaA5 co-expressed with *Cb*Maf. Modifications (predicted by Modscore>19) with Neu5Ac and KDO are indicated by @ and #, respectively.

```
1    MAIINHNLNA MNAHRQMAIN TGSNGKAIEK LSSGLRINRA GDDAAGLAIS
51   EKMRGQIRGL NQASRNSQDS ISLIQTAEGA LNETHSILQR MRELAVQSAN
101  DTNVGKGAKS ISGMIETIDN AINTVSGERA KLGANQNRLE HTIANLDNSA
151  ENLQAAESRI RDVDMAKEMM NFTKTNILTQ AAQAMLAQAN QAPQGVLQLL
201  RHHHHHH
```

e3. Sequence coverage (shown by underlining) of trypsin digested *Cb*FlaA5 co-expressed with *Cb*Maf with confidently assigned glycosites (validated by at least one MS/MS spectrum containing at least one glycan-modified fragment ion). Modifications with Neu5Ac and KDO are indicated by @ and #, respectively.

```
1    MAIINHNLNA MNAHRQMAIN TGSNGKAIEK LSSGLRINRA GDDAAGLAIS
51   EKMRGQIRGL NQASRNSQDS ISLIQTAEGA LNETHSILQR MRELAVQSAN
101  DTNVGKGAKS ISGMIETIDN AINTVSGERA KLGANQNRLE HTIANLDNSA
151  ENLQAAESRI RDVDMAKEMM NFTKTNILTQ AAQAMLAQAN QAPQGVLQLL
201  RHHHHHH
```

e4. Sequence coverage (shown by underlining) of Glu-C digested *Cb*FlaA5.

```
1    MAIINHNLNA MNAHRQMAIN TGSNGKAIEK LSSGLRINRA GDDAAGLAIS
51   EKMRGQIRGL NQASRNSQDS ISLIQTAEGA LNETHSILQR MRELAVQSAN
101  DTNVGKGAKS ISGMIETIDN AINTVSGERA KLGANQNRLE HTIANLDNSA
151  ENLQAAESRI RDVDMAKEMM NFTKTNILTQ AAQAMLAQAN QAPQGVLQLL
201  RHHHHHH
```

e5. Sequence coverage (shown by underlining) of Glu-C digested *Cb*FlaA5 co-expressed with *Cb*Maf. Modifications (predicted by Modscore>19) with Neu5Ac are indicated by @. All glycosites are confidently assigned (validated by at least one MS/MS spectrum containing at least one glycan-modified fragment ion).

```
1    MAIINHNLNA MNAHRQMAIN TGSNGKAIEK LSSGLRINRA GDDAAGLAIS
51   EKMRGQIRGL NQASRNSQDS ISLIQTAEGA LNETHSILQR MRELAVQSAN
101  DTNVGKGAKS ISGMIETIDN AINTVSGERA KLGANQNRLE HTIANLDNSA
151  ENLQAAESRI RDVDMAKEMM NFTKTNILTQ AAQAMLAQAN QAPQGVLQLL
201  RHHHHHH
```

e6. Domain architecture of full length *Cb*FlaA5. Glycosites with Neu5Ac and KDO are indicated by symbols (♦) and (⬡), respectively.

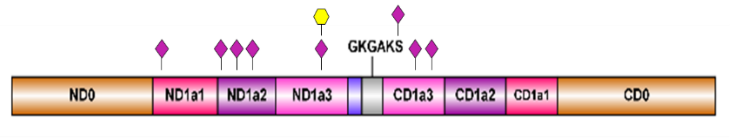

e7. MS/MS spectrum for a trypsin digested peptide from *Cb*FlaA5 co-expressed with *Cb*Maf

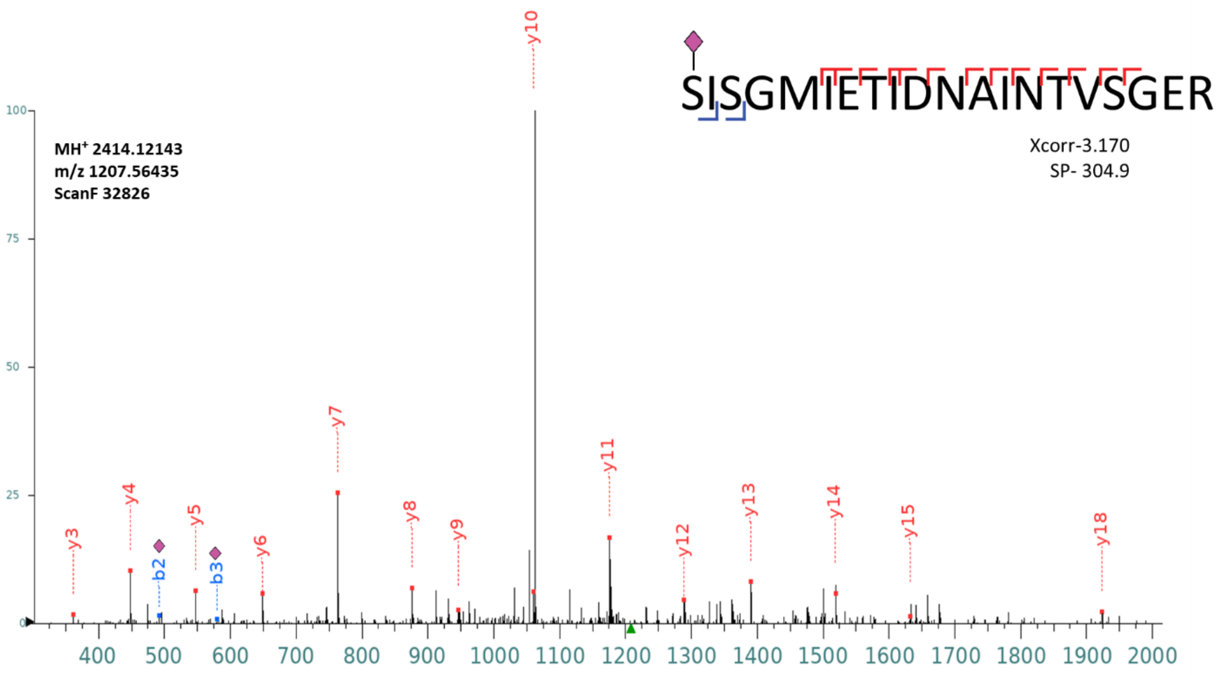

e8. MS/MS spectrum for a trypsin digested peptide from *Cb*FlaA5 co-expressed with *Cb*Maf

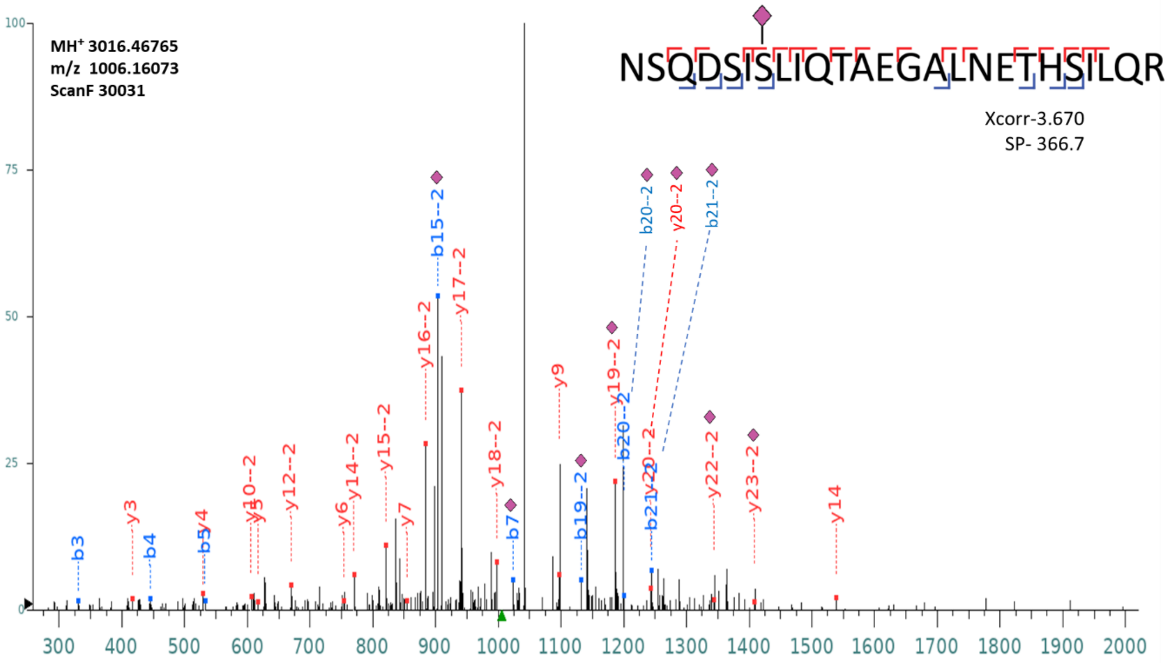

e9. MS/MS spectrum for a trypsin digested peptide from *Cb*FlaA5 co-expressed with *Cb*Maf

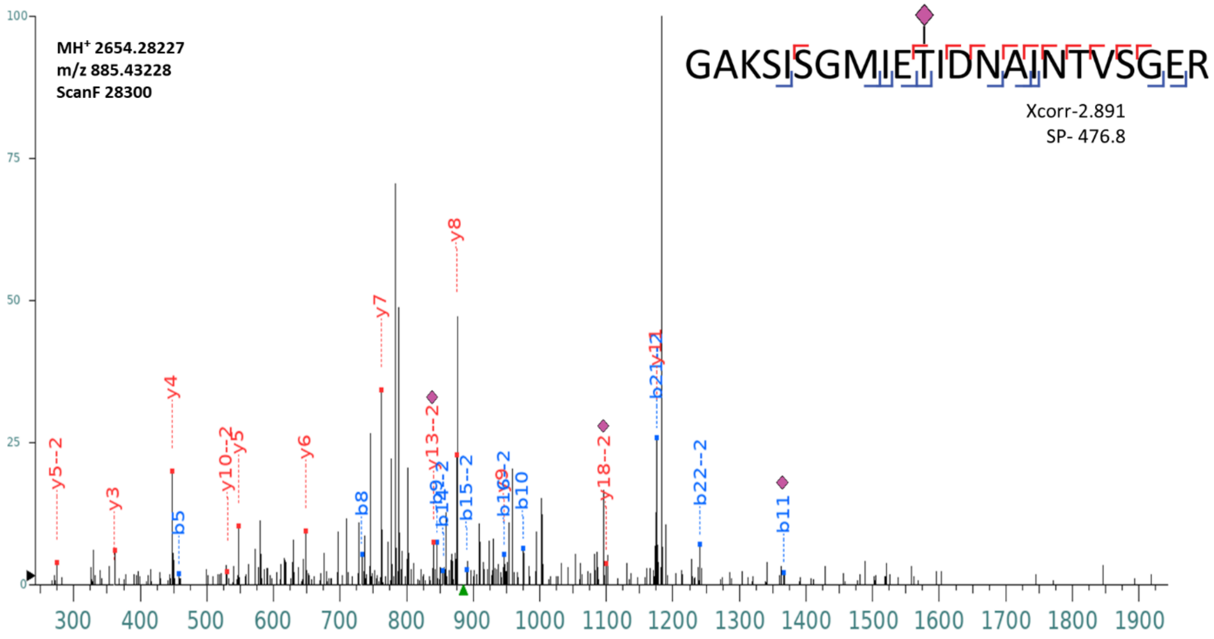

e10. MS/MS spectrum for a trypsin digested peptide from *Cb*FlaA5 co-expressed with *Cb*Maf

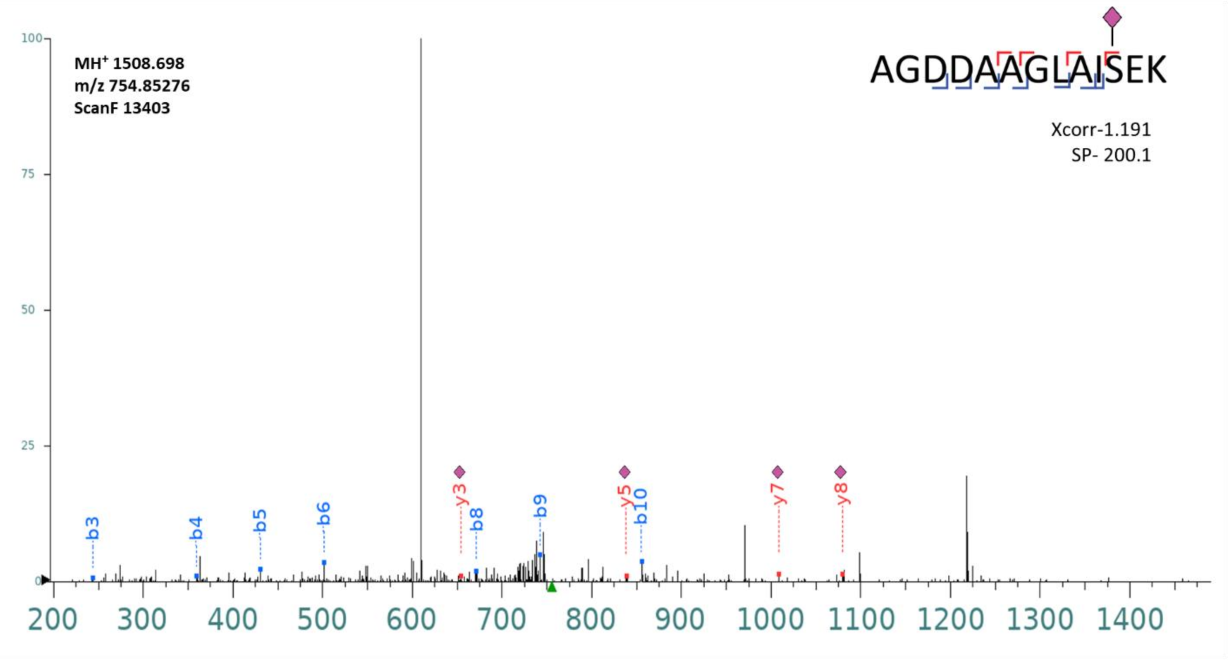

e11. MS/MS spectrum for a GluC digested peptide from *Cb*FlaA5 co-expressed with *Cb*Maf

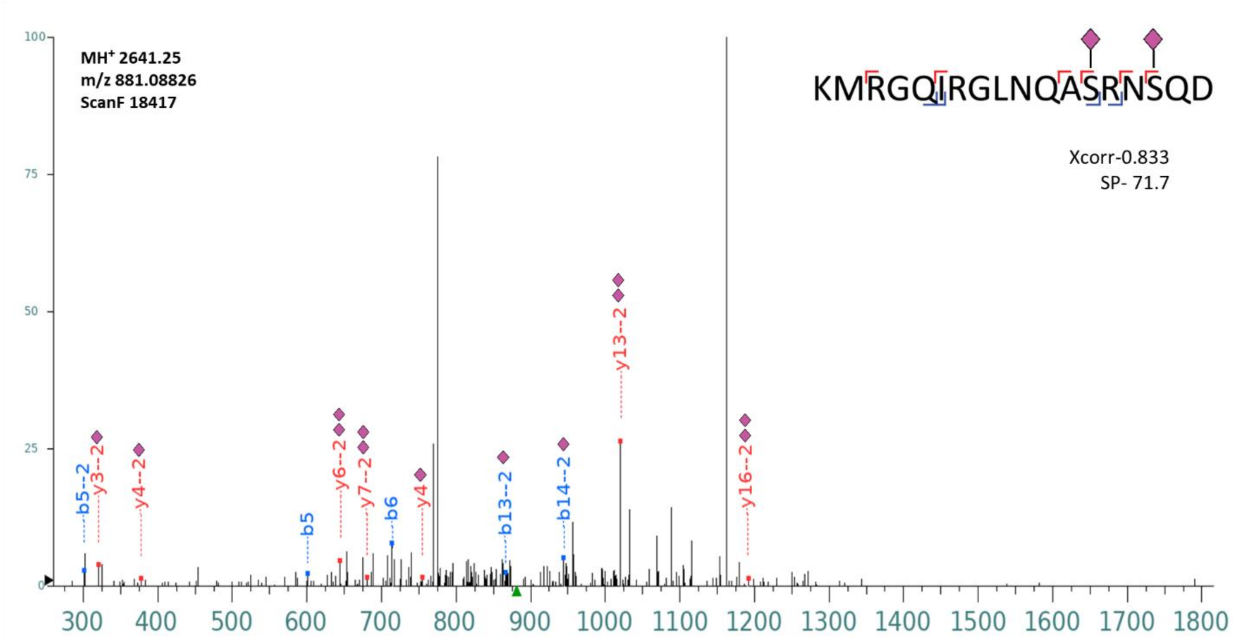

e12. MS/MS spectrum for a trypsin digested peptide from *Cb*FlaA5 co-expressed with *Cb*Maf

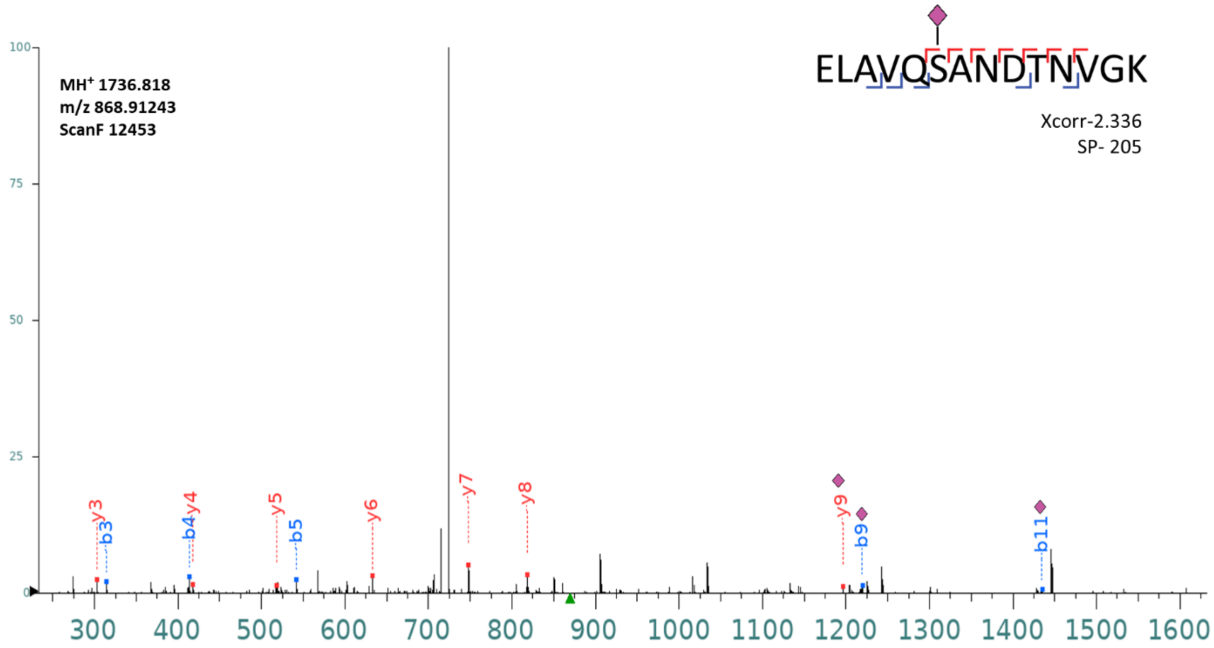

e13. MS/MS spectrum for a trypsin digested peptide from *Cb*FlaA5 co-expressed with *Cb*Maf

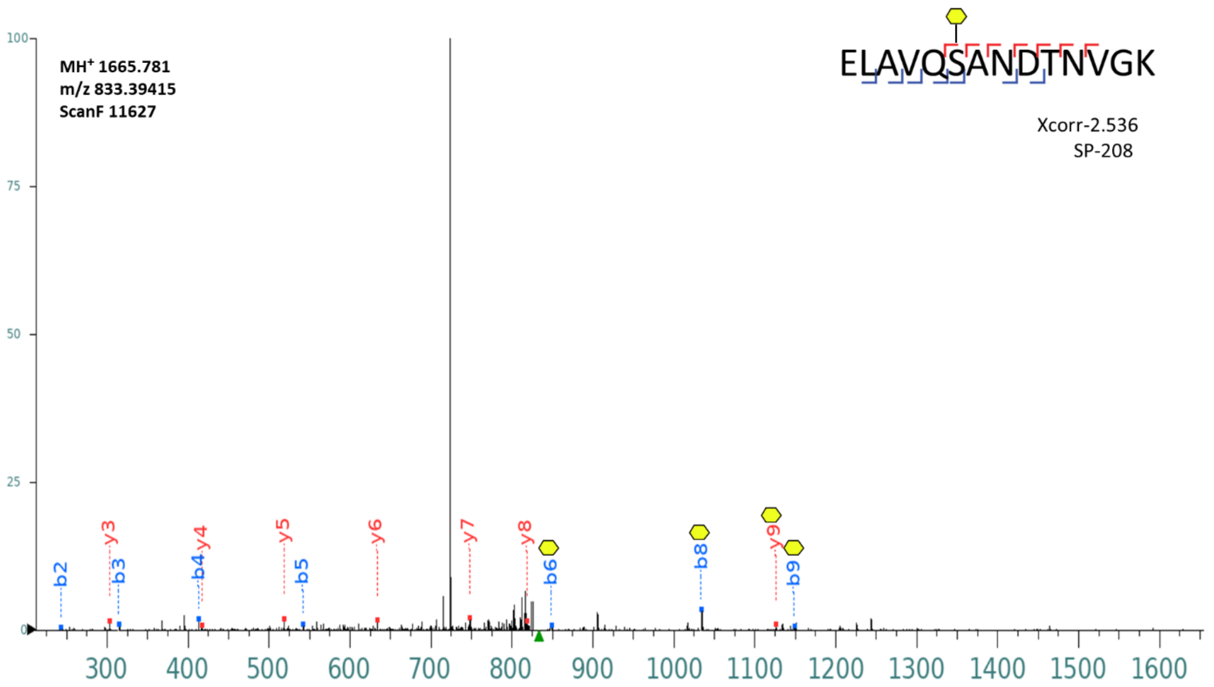

e14. MS/MS spectrum for a trypsin digested peptide from *Cb*FlaA5 co-expressed with *Cb*Maf

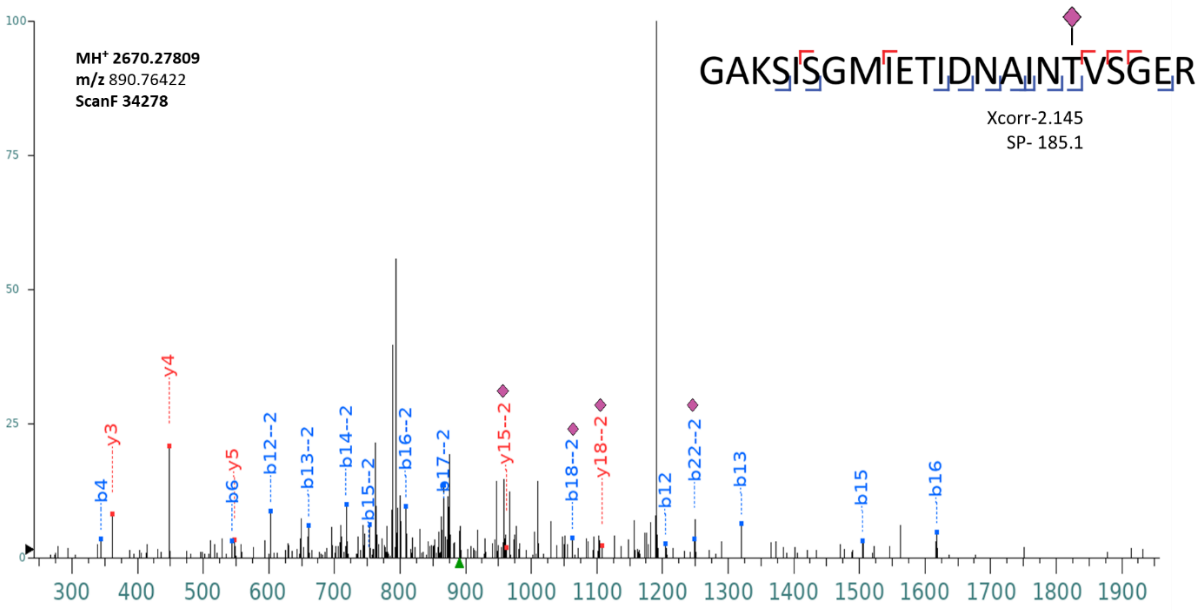

f1. Sequence coverage (shown by underlining) of trypsin digested *Cb*FlaA7.

```
1   MAIINHNLNA MNAHRQMAIN TGSNGKAIEK LSSGLRINRA GDDAAGLAIS
51  EKMRGQIRGL NQASRNSQDS ISLIQTAEGA LNETHSILQR MRELAVQSAN
101 DTNVKVDRDN LQKEVAELQN EINRIASQTQ FNTKNLLNMG TSALKVGSIN
151 IGSGKGAKSI SGMIETIDNA INTVSGERAK LGANQNRLEH TIANLDNSAE
201 NLQAAESRIR DVDMAKEMMN FTKTNILTQA AQAMLAQANQ APQGVLQLLR
251 HHHHHH
```

f2. Sequence coverage (shown by underlining) of trypsin digested *Cb*FlaA7 co-expressed with *Cb*Maf. Modifications (predicted by Modscore>19) with Neu5Ac and KDO are indicated by @ and #, respectively.

```
                                                         @
1   MAIINHNLNA MNAHRQMAIN TGSNGKAIEK LSSGLRINRA GDDAAGLAIS
51  EKMRGQIRGL NQASRNSQDS ISLIQTAEGA LNETHSILQR MRELAVQSAN
                                @ @             @@     @#
101 DTNVKVDRDN LQKEVAELQN EINRIASQTQ FNTKNLLNMG TSALKVGSIN
      @     @      @        @ @
151 IGSGKGAKSI SGMIETIDNA INTVSGERAK LGANQNRLEH TIANLDNSAE
201 NLQAAESRIR DVDMAKEMMN FTKTNILTQA AQAMLAQANQ APQGVLQLLR
251 HHHHHH
```

f3. Sequence coverage (shown by underlining) of trypsin digested *Cb*FlaA7 co-expressed with *Cb*Maf with confidently assigned glycosites (validated by at least one MS/MS spectrum containing at least one glycan-modified fragment ion). Modifications with Neu5Ac and KDO are indicated by @ and #, respectively.

```
                                                         @
1   MAIINHNLNA MNAHRQMAIN TGSNGKAIEK LSSGLRINRA GDDAAGLAIS
51  EKMRGQIRGL NQASRNSQDS ISLIQTAEGA LNETHSILQR MRELAVQSAN
                                @               @@     @#
101 DTNVKVDRDN LQKEVAELQN EINRIASQTQ FNTKNLLNMG TSALKVGSIN
      @     @      @        @
151 IGSGKGAKSI SGMIETIDNA INTVSGERAK LGANQNRLEH TIANLDNSAE
201 NLQAAESRIR DVDMAKEMMN FTKTNILTQA AQAMLAQANQ APQGVLQLLR
251 HHHHHH
```

f4. Sequence coverage (shown by underlining) of Glu-C digested *Cb*FlaA7.

```
1   MAIINHNLNA MNAHRQMAIN TGSNGKAIEK LSSGLRINRA GDDAAGLAIS
51  EKMRGQIRGL NQASRNSQDS ISLIQTAEGA LNETHSILQR MRELAVQSAN
101 DTNVKVDRDN LQKEVAELQN EINRIASQTQ FNTKNLLNMG TSALKVGSIN
151 IGSGKGAKSI SGMIETIDNA INTVSGERAK LGANQNRLEH TIANLDNSAE
201 NLQAAESRIR DVDMAKEMMN FTKTNILTQA AQAMLAQANQ APQGVLQLLR
251 HHHHHH
```

f5. Sequence coverage (shown by underlining) of Glu-C digested *Cb*FlaA7 co-expressed with *Cb*Maf.

```
1   MAIINHNLNA MNAHRQMAIN TGSNGKAIEK LSSGLRINRA GDDAAGLAIS
51  EKMRGQIRGL NQASRNSQDS ISLIQTAEGA LNETHSILQR MRELAVQSAN
101 DTNVKVDRDN LQKEVAELQN EINRIASQTQ FNTKNLLNMG TSALKVGSIN
151 IGSGKGAKSI SGMIETIDNA INTVSGERAK LGANQNRLEH TIANLDNSAE
201 NLQAAESRIR DVDMAKEMMN FTKTNILTQA AQAMLAQANQ APQGVLQLLR
251 HHHHHH
```

f6. Domain architecture of full length *Cb*FlaA7. Glycosites with Neu5Ac and KDO are indicated by symbols (♦) and (⬡), respectively.

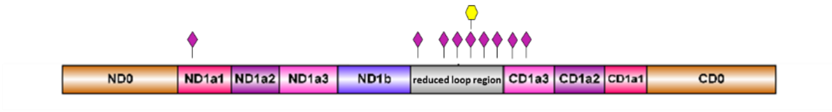

f7. MS/MS spectrum for a trypsin digested peptide from *Cb*FlaA7 co-expressed with *Cb*Maf

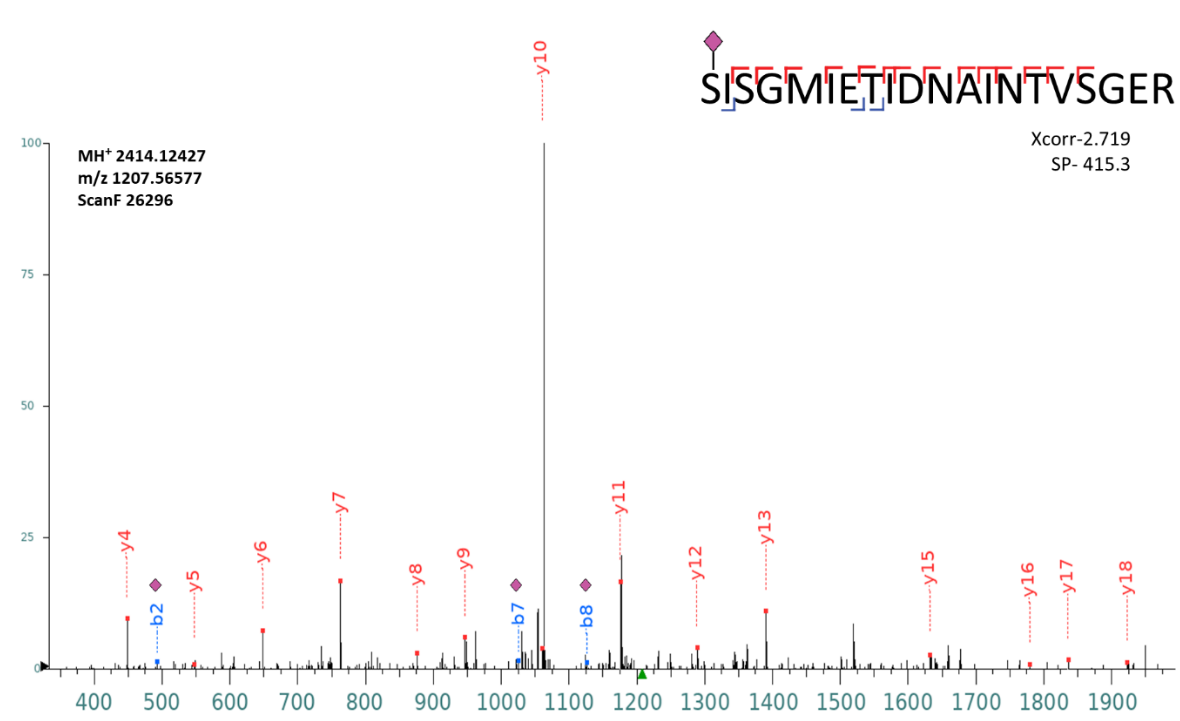

f8. MS/MS spectrum for a trypsin digested peptide from *Cb*FlaA7 co-expressed with *Cb*Maf

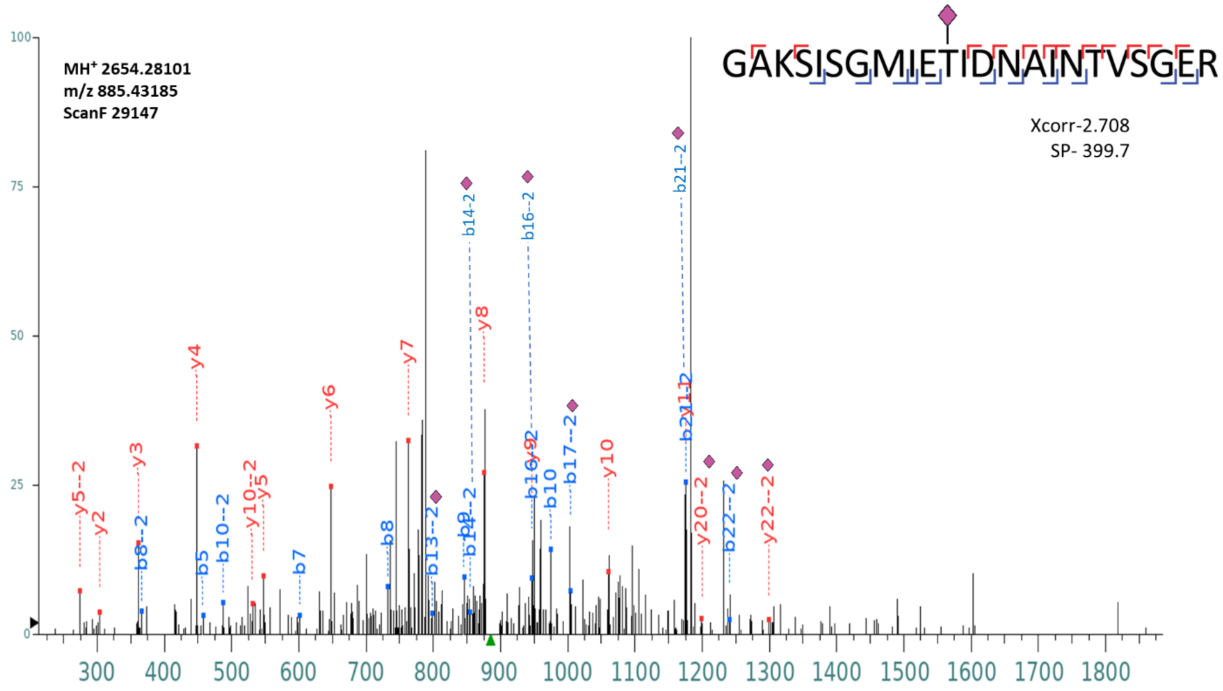

f9. MS/MS spectrum for a trypsin digested peptide from *Cb*FlaA7 co-expressed with *Cb*Maf

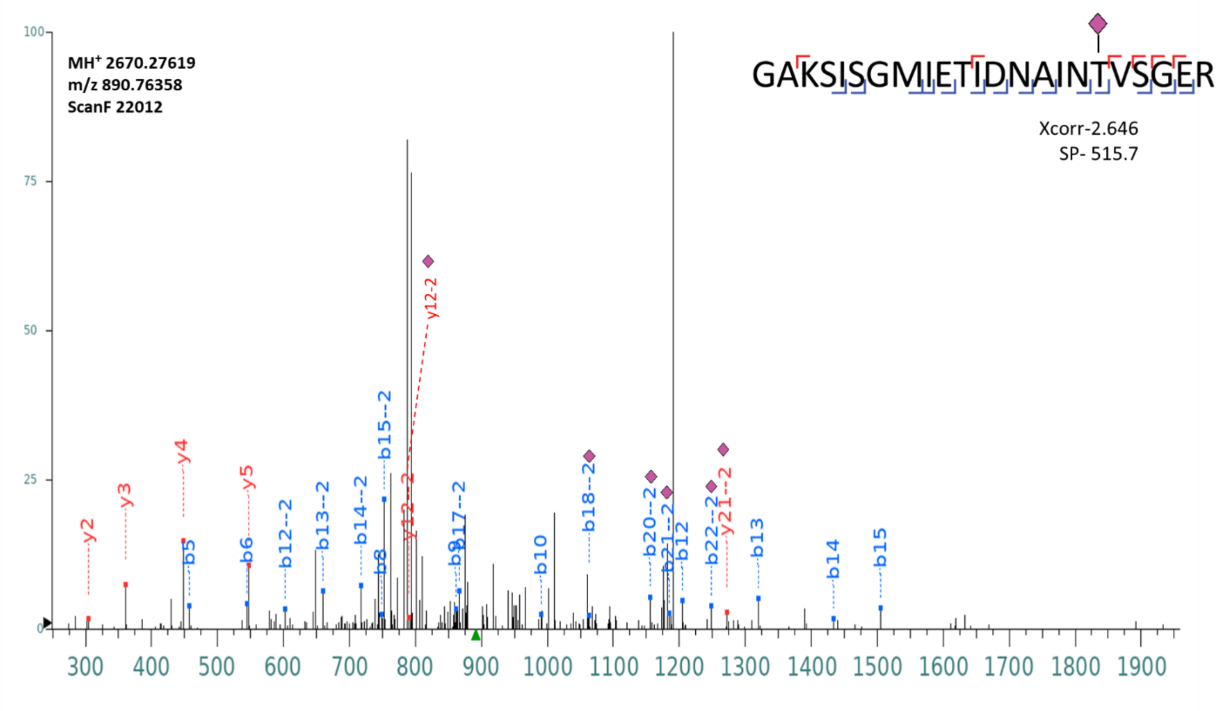

f10. MS/MS spectrum for a trypsin digested peptide from *Cb*FlaA7 co-expressed with *Cb*Maf

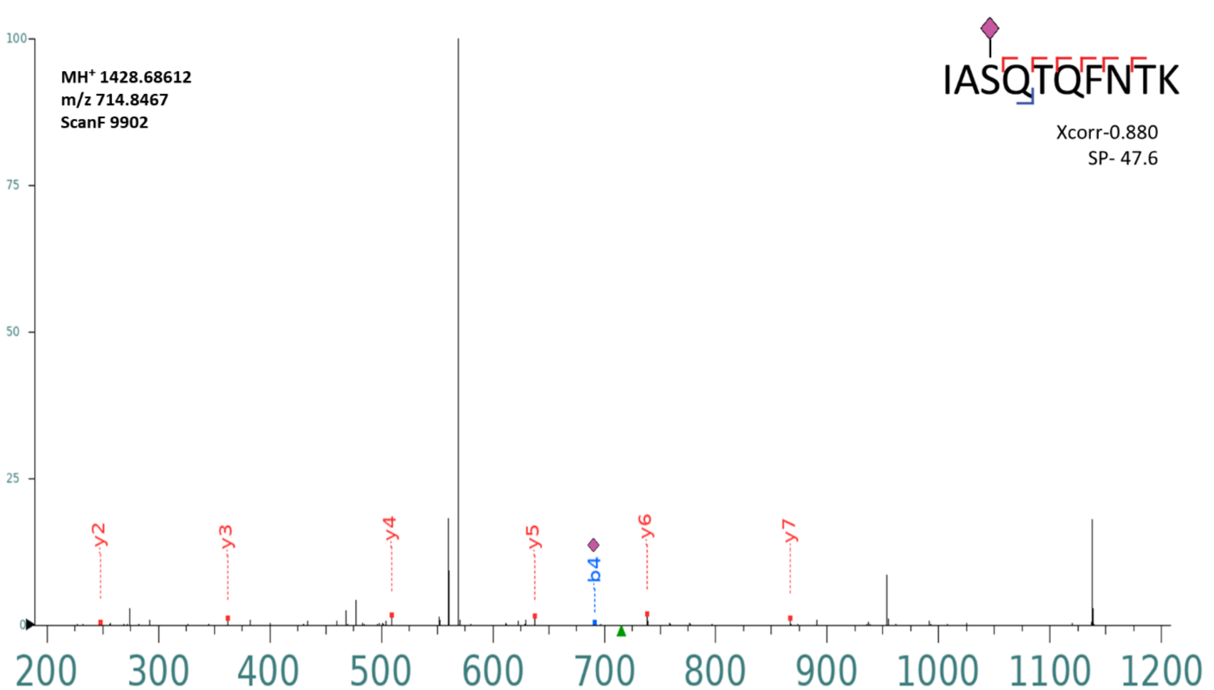

f11. MS/MS spectrum for a trypsin digested peptide from *Cb*FlaA7 co-expressed with *Cb*Maf

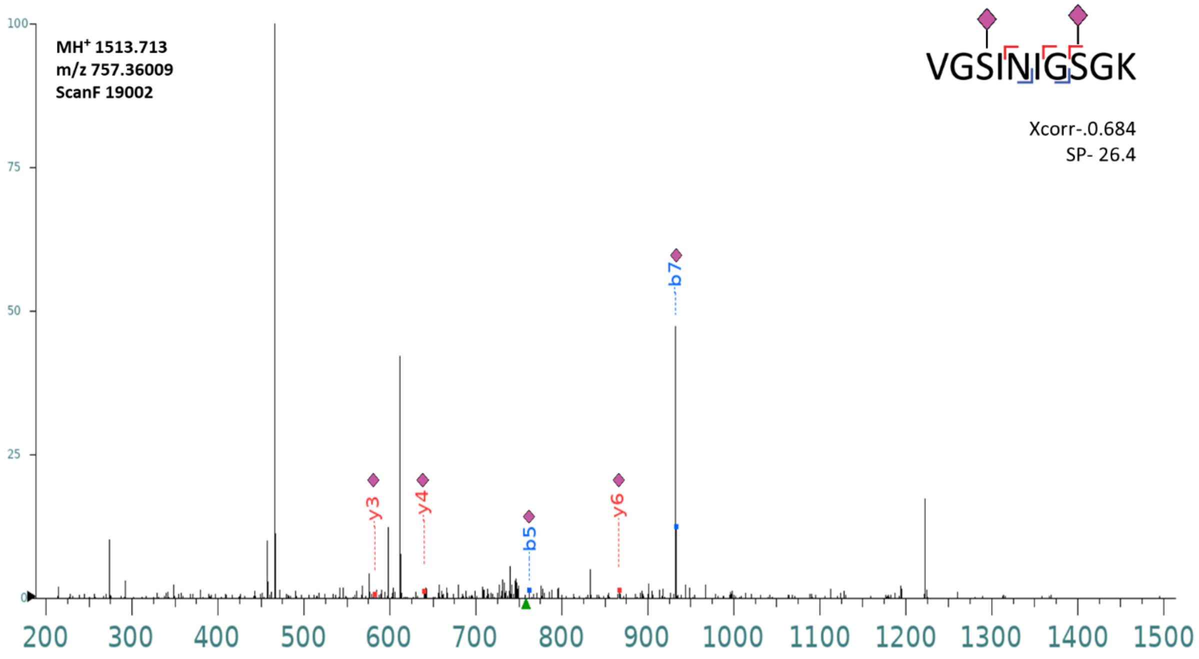

f12. MS/MS spectrum for a trypsin digested peptide from *Cb*FlaA7 co-expressed with *Cb*Maf

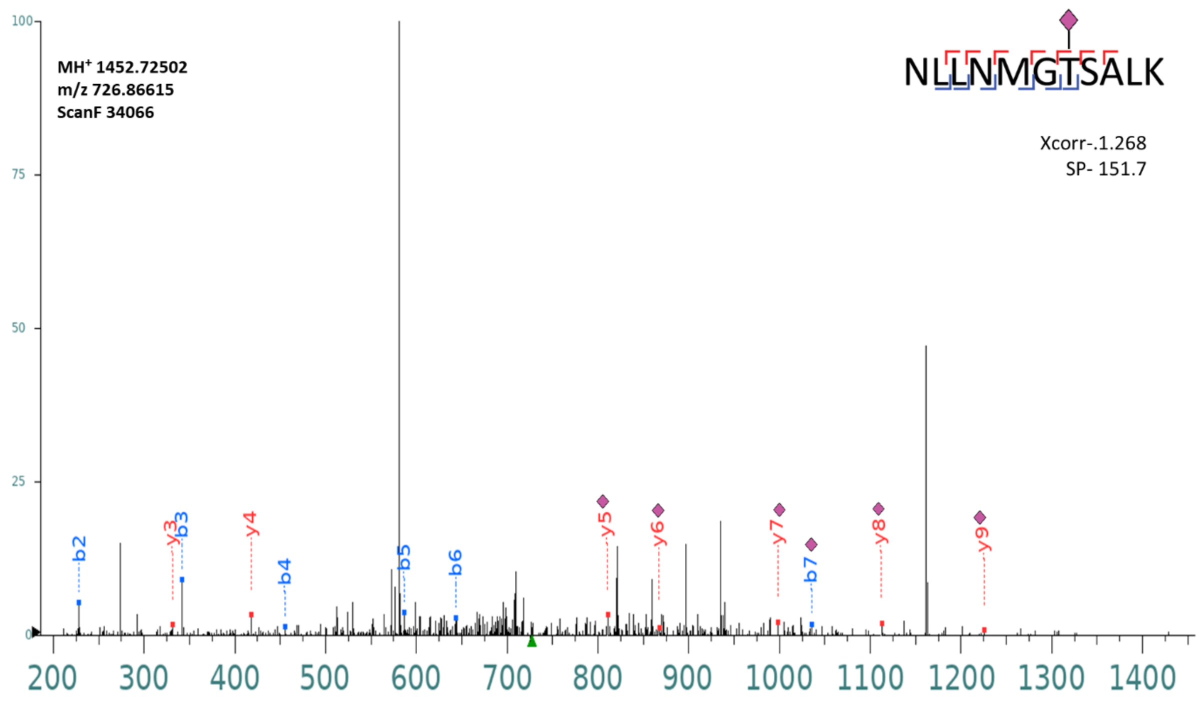

f13. MS/MS spectrum for a trypsin digested peptide from *Cb*FlaA7 co-expressed with *Cb*Maf

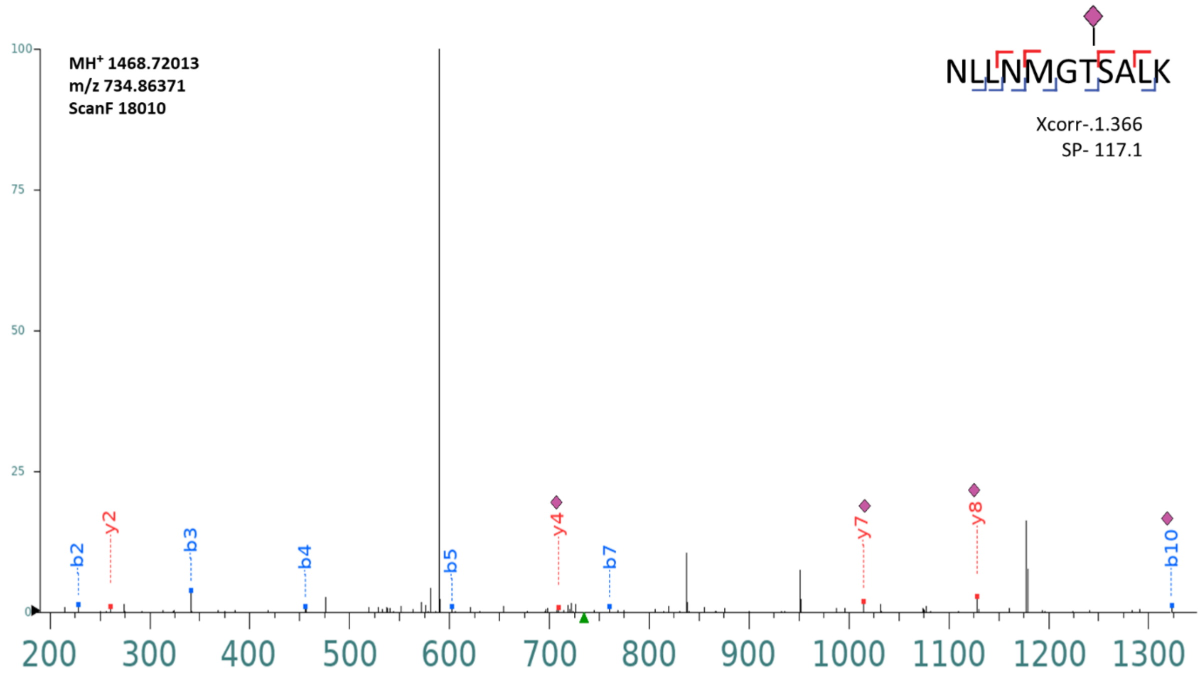

f14. MS/MS spectrum for a trypsin digested peptide from *Cb*FlaA7 co-expressed with *Cb*Maf

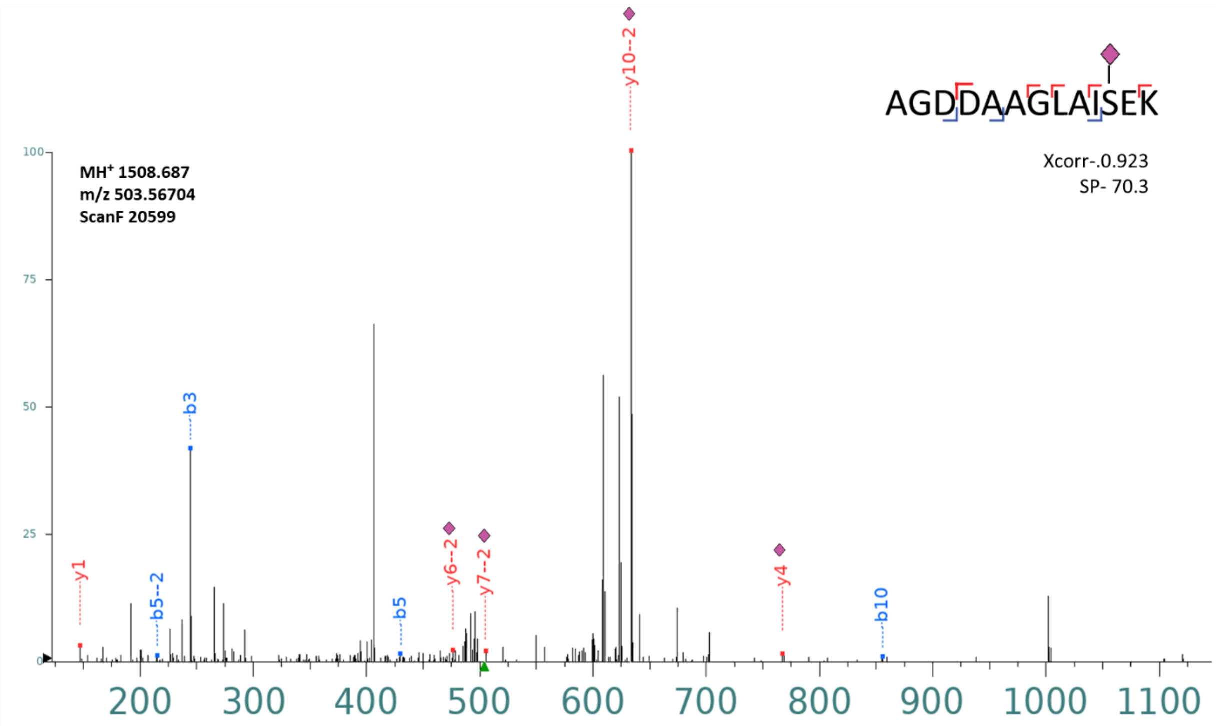

f15. MS/MS spectrum for a trypsin digested peptide from *Cb*FlaA7 co-expressed with *Cb*Maf

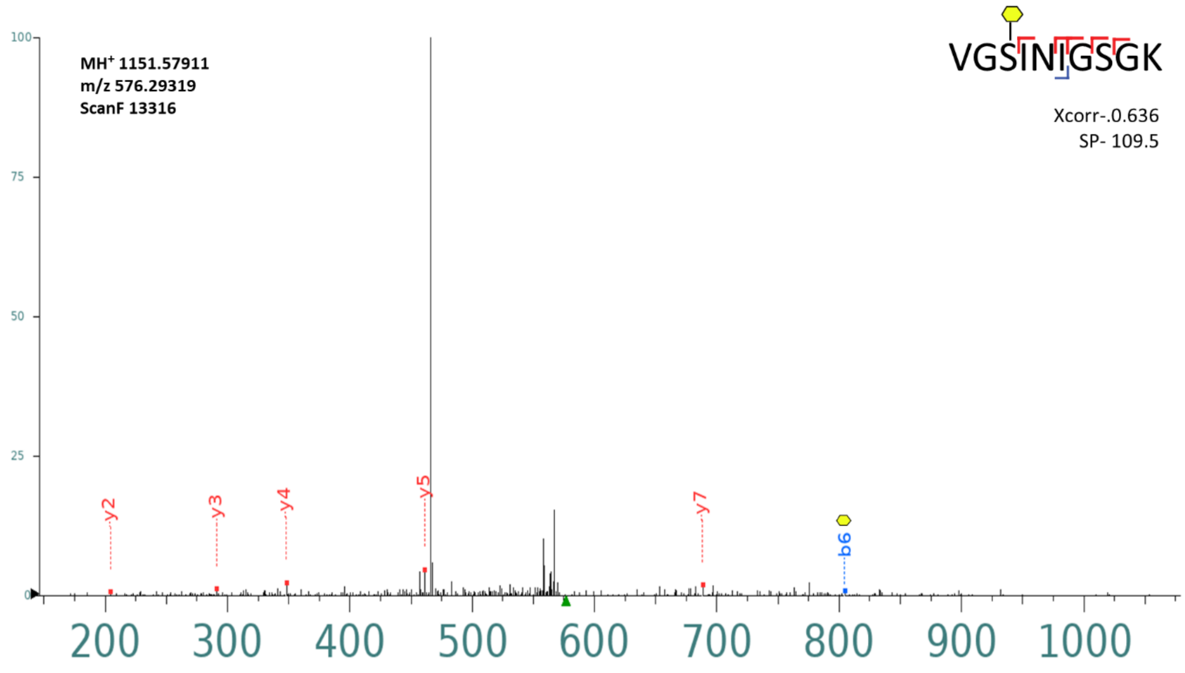

g1. Sequence coverage (shown by underlining) of trypsin digested *Cb*FlaA4_A5.

```
1    MAASRNSQDS ISLIQTAEGA LNETHSILQR MRELAVQSAN DTNVGKGAKS
51   ISGMIETIDN AINTVSGERA KLGANQNRLE HTIANLLEHH HHHH
```

g2. Sequence coverage (shown by underlining) of trypsin digested *Cb*FlaA4_A5 co-expressed with *Cb*Maf.

```
1    MAASRNSQDS ISLIQTAEGA LNETHSILQR MRELAVQSAN DTNVGKGAKS
51   ISGMIETIDN AINTVSGERA KLGANQNRLE HTIANLLEHH HHHH
```

h1. Sequence coverage (shown by underlining) of trypsin digested *Cb*FlaD0A7loop.

```
1    MAIINHNLNA MNAHRQMAIN TGSNGKAIEK LSSGLRINRA GDDSQTQFNT
51   KNLLNMGTSA LKVGSINIGS GKGAKSDVDM AKEMMNFTKT NILTQAAQAM
101  LAQANQAPQG VLQLLRLEHH HHHH
```

h2. Sequence coverage (shown by underlining) of trypsin digested *Cb*FlaD0A7loop co-expressed with *Cb*Maf.

```
1    MAIINHNLNA MNAHRQMAIN TGSNGKAIEK LSSGLRINRA GDDSQTQFNT
51   KNLLNMGTSA LKVGSINIGS GKGAKSDVDM AKEMMNFTKT NILTQAAQAM
101  LAQANQAPQG VLQLLRLEHH HHHH
```

i1. Sequence coverage (shown by underlining) of trypsin digested *Sr*NaFLD.

```
1    MAVRSLLRTS SSLAKEIAPG RTISGELTVT NPGKRHTPAT KLSLTVPQGW
51   TATLGAPRVR PLGPGESVKV PISVSAPEGV APAPYTLGLY QRTGRMGTTT
101  ALPLTVNRPN LSLGKPATQK STGYDAPASR AVDGNTGGDW SAGSTTHTAE
151  PEKQAWWQVD LGASARLDSV DVWNRLDCCA DRLKDFWVMA SDQPFTTDDL
201  DQARTAPGVT AVHVGEQAGS PSKVKLPEGT RGRYVRIQLA SPSNPLSLAE
251  VQVRGLEHHH HHH
```

i2. Sequence coverage (shown by underlining) of trypsin digested *Sr*NaFLD co-expressed with *Cb*Maf.

```
1    MAVRSLLRTS SSLAKEIAPG RTISGELTVT NPGKRHTPAT KLSLTVPQGW
51   TATLGAPRVR PLGPGESVKV PISVSAPEGV APAPYTLGLY QRTGRMGTTT
101  ALPLTVNRPN LSLGKPATQK STGYDAPASR AVDGNTGGDW SAGSTTHTAE
151  PEKQAWWQVD LGASARLDSV DVWNRLDCCA DRLKDFWVMA SDQPFTTDDL
201  DQARTAPGVT AVHVGEQAGS PSKVKLPEGT RGRYVRIQLA SPSNPLSLAE
251  VQVRGLEHHH HHH
```

j1. Sequence coverage (shown by underlining) of trypsin digested *Sr*Na_*Cb*FlaA1_*Sr*FLD .

```
1   MAVRSLLRTS SSLAKEIAPG RTISGELTVT NPGKRHTPAT KLSLTVPQGW
51  TATLGAPRVR PLGPGESVKV PISVSAPEGV APAPYTLGLY QRTGRMGTTT
101 ALPLTVNDTN VKVDRDNLQK EVAELQNEIN RIASQTQFNT KNLLNGSAKS
151 MVFQIGANKD QVMELTIAGM GTSALKVGSI NIGSGKGAKS RPNLSLGKPA
201 TQKSTGYDAP ASRAVDGNTG GDWSAGSTTH TAEPEKQAWW QVDLGASARL
251 DSVDVWNRLD CCADRLKDFW VMASDQPFTT DDLDQARTAP GVTAVHVGEQ
301 AGSPSKVKLP EGTRGRYVRI QLASPSNPLS LAEVQVRGLE HHHHHH
```

j2. Sequence coverage (shown by underlining) of trypsin digested *Sr*Na_*Cb*FlaA1_*Sr*FLD co-expressed with *Cb*Maf. Modifications (predicted by Modscore>19) with Neu5Ac are indicated by @.

```
1   MAVRSLLRTS SSLAKEIAPG RTISGELTVT NPGKRHTPAT KLSLTVPQGW
51  TATLGAPRVR PLGPGESVKV PISVSAPEGV APAPYTLGLY QRTGRMGTTT
101 ALPLTVNDTN VKVDRDNLQK EVAELQNEIN RIASQTQFNT KNLLNGSAKS
151 MVFQIGANKD QVMELTIAGM GTSALKVGSI NIGSGKGAKS RPNLSLGKPA
201 TQKSTGYDAP ASRAVDGNTG GDWSAGSTTH TAEPEKQAWW QVDLGASARL
251 DSVDVWNRLD CCADRLKDFW VMASDQPFTT DDLDQARTAP GVTAVHVGEQ
301 AGSPSKVKLP EGTRGRYVRI QLASPSNPLS LAEVQVRGLE HHHHHH
```

j3. Sequence coverage (shown by underlining) of trypsin digested *Sr*Na_*Cb*FlaA1_*Sr*FLD co-expressed with *Cb*Maf with confidently assigned glycosites (validated by at least one MS/MS spectrum containing at least one glycan-modified fragment ion). Modifications with Neu5Ac are indicated by @.

```
1   MAVRSLLRTS SSLAKEIAPG RTISGELTVT NPGKRHTPAT KLSLTVPQGW
51  TATLGAPRVR PLGPGESVKV PISVSAPEGV APAPYTLGLY QRTGRMGTTT
101 ALPLTVNDTN VKVDRDNLQK EVAELQNEIN RIASQTQFNT KNLLNGSAKS
151 MVFQIGANKD QVMELTIAGM GTSALKVGSI NIGSGKGAKS RPNLSLGKPA
201 TQKSTGYDAP ASRAVDGNTG GDWSAGSTTH TAEPEKQAWW QVDLGASARL
251 DSVDVWNRLD CCADRLKDFW VMASDQPFTT DDLDQARTAP GVTAVHVGEQ
301 AGSPSKVKLP EGTRGRYVRI QLASPSNPLS LAEVQVRGLE HHHHHH
```

j4. Domain architecture of *Sr*Na_*Cb*FlaA1_*Sr*FLD. Glycosites with Neu5Ac is indicated by symbol (♦).

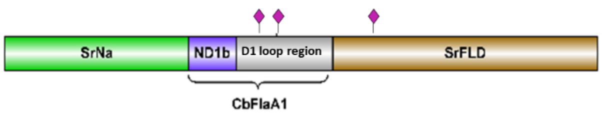

j5. MS/MS spectrum for a trypsin digested peptide from *Sr*Na*Cb*FlaA1*Sr*FLD co-expressed with *Cb*Maf

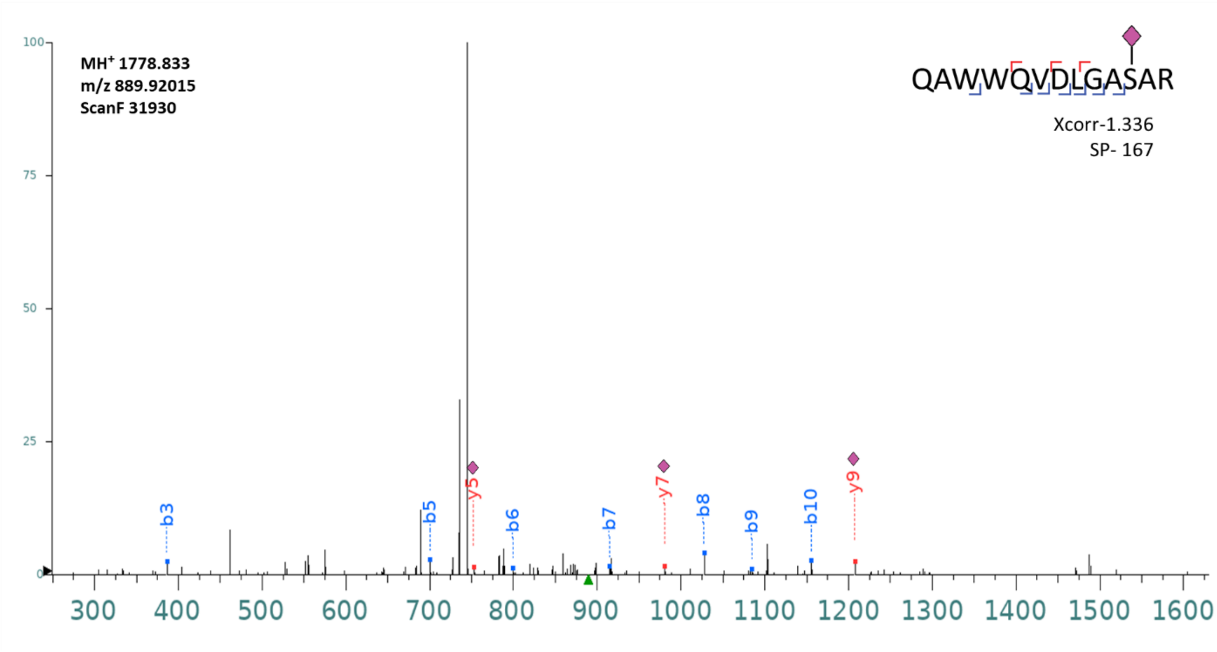

j6. MS/MS spectrum for a trypsin digested peptide from *Sr*Na*Cb*FlaA1*Sr*FLD co-expressed with *Cb*Maf

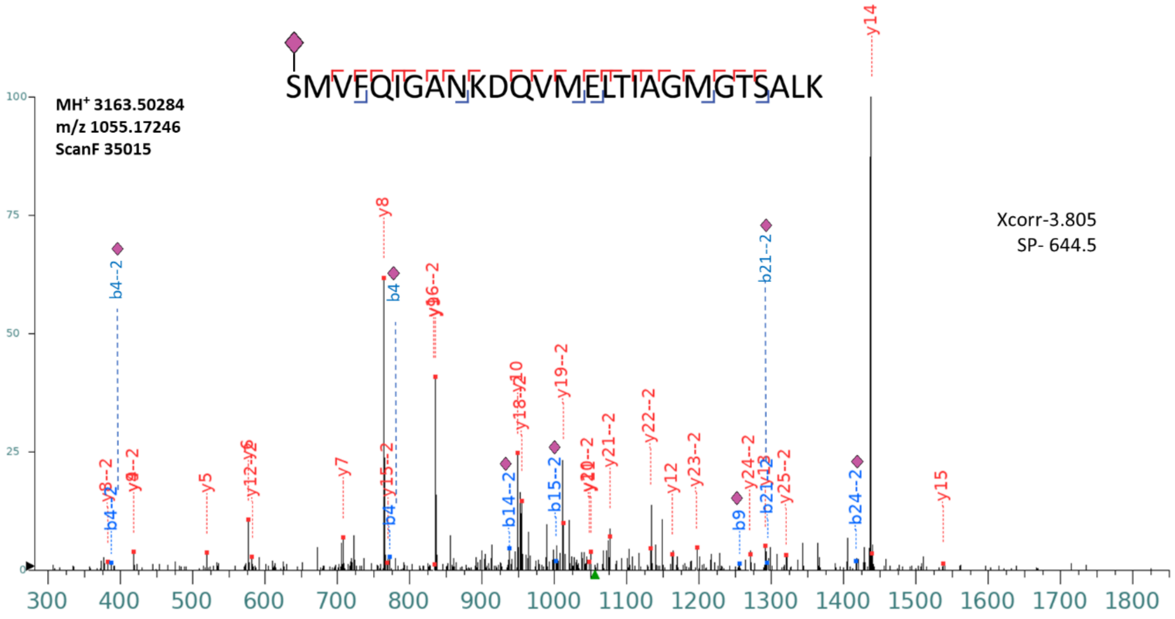

j7. MS/MS spectrum for a trypsin digested peptide from *Sr*Na*Cb*FlaA1*Sr*FLD co-expressed with *Cb*Maf

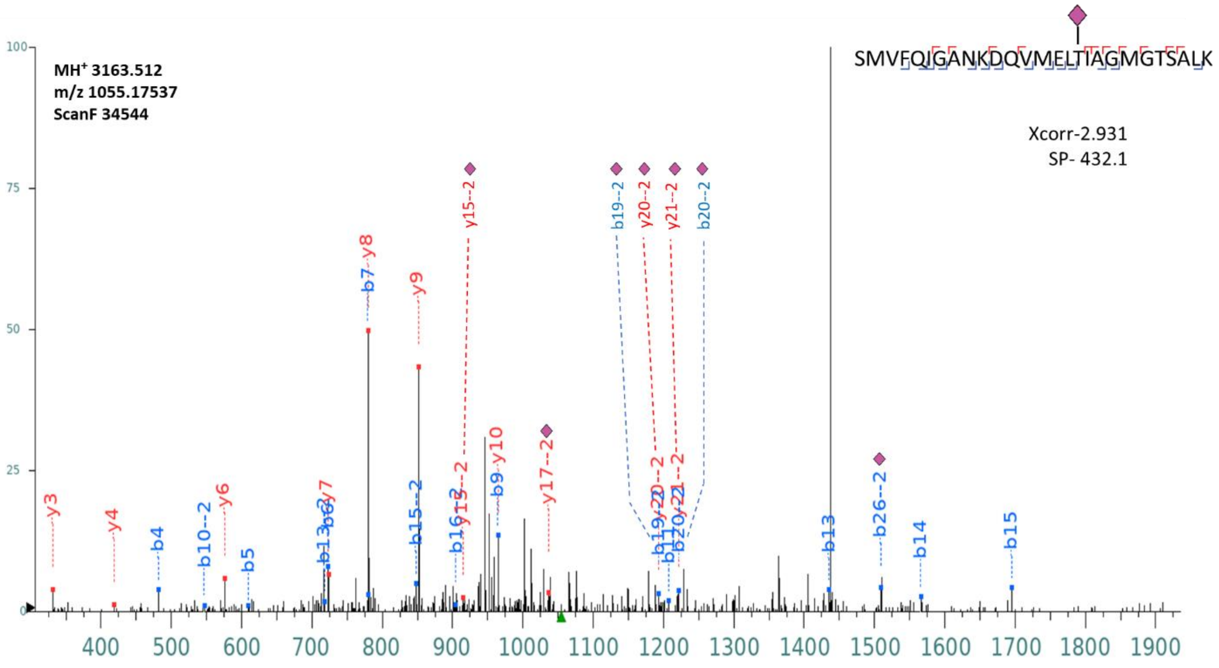

k1. Sequence coverage (shown by underlining) of trypsin digested GKGAKS-*Sr*NaFLD.

```
1    MAGKGAKSVR SLLRTSSSLA KEIAPGRTIS GELTVTNPGK RHTPATKLSL
51   TVPQGWTATL GAPRVRPLGP GESVKVPISV SAPEGVAPAP YTLGLYQRTG
101  RMGTTTALPL TVNRPNLSLG KPATQKSTGY DAPASRAVDG NTGGDWSAGS
151  TTHTAEPEKQ AWWQVDLGAS ARLDSVDVWN RLDCCADRLK DFWVMASDQP
201  FTTDDLDQAR TAPGVTAVHV GEQAGSPSKV KLPEGTRGRY VRIQLASPSN
251  PLSLAEVQVR GLEHHHHHH
```

k2. Sequence coverage (shown by underlining) of trypsin digested GKGAKS-*Sr*NaFLD co-expressed with *Cb*Maf.

```
1    MAGKGAKSVR SLLRTSSSLA KEIAPGRTIS GELTVTNPGK RHTPATKLSL
51   TVPQGWTATL GAPRVRPLGP GESVKVPISV SAPEGVAPAP YTLGLYQRTG
101  RMGTTTALPL TVNRPNLSLG KPATQKSTGY DAPASRAVDG NTGGDWSAGS
151  TTHTAEPEKQ AWWQVDLGAS ARLDSVDVWN RLDCCADRLK DFWVMASDQP
201  FTTDDLDQAR TAPGVTAVHV GEQAGSPSKV KLPEGTRGRY VRIQLASPSN
251  PLSLAEVQVR GLEHHHHHH
```

l1. **Sequence coverage (shown by underlining) of trypsin digested *Cb*Fla_A1helix_GKGAKS_*Sr*NaFLD.**

```
1   MADTNVKVDR DNLQKEVAEL QNEINRIAGK GAKSVRSLLR TSSSLAKEIA
51  PGRTISGELT VTNPGKRHTP ATKLSLTVPQ GWTATLGAPR VRPLGPGESV
101 KVPISVSAPE GVAPAPYTLG LYQRTGRMGT TTALPLTVNR PNLSLGKPAT
151 QKSTGYDAPA SRAVDGNTGG DWSAGSTTHT AEPEKQAWWQ VDLGASARLD
201 SVDVWNRLDC CADRLKDFWV MASDQPFTTD DLDQARTAPG VTAVHVGEQA
251 GSPSKVKLPE GTRGRYVRIQ LASPSNPLSL AEVQVRGLEH HHHHH
```

l2. **Sequence coverage (shown by underlining) of trypsin digested *Cb*Fla_A1helix_GKGAKS_*Sr*NaFLD co-expressed with *Cb*Maf.**

```
1   MADTNVKVDR DNLQKEVAEL QNEINRIAGK GAKSVRSLLR TSSSLAKEIA
51  PGRTISGELT VTNPGKRHTP ATKLSLTVPQ GWTATLGAPR VRPLGPGESV
101 KVPISVSAPE GVAPAPYTLG LYQRTGRMGT TTALPLTVNR PNLSLGKPAT
151 QKSTGYDAPA SRAVDGNTGG DWSAGSTTHT AEPEKQAWWQ VDLGASARLD
201 SVDVWNRLDC CADRLKDFWV MASDQPFTTD DLDQARTAPG VTAVHVGEQA
251 GSPSKVKLPE GTRGRYVRIQ LASPSNPLSL AEVQVRGLEH HHHHH
```

m1. **Sequence coverage (shown by underlining) of trypsin digested *Cb*Fla co-expressed with *Gk*Maf. Modifications (predicted by Modscore>19) with Neu5Ac and KDO are indicated by @ and #, respectively.**

```
                                             @
1   MAIINHNLNA MNAHRQMAIN TGSNGKAIEK LSSGLRINRA GDDAAGLAIS
51  EKMRGQIRGL NQASRNSQDS ISLIQTAEGA LNETHSILQR MRELAVQSAN
101 DTNVKVDRDN LQKEVAELQN EINRIASQTQ FNTKNLLNGS AKSMVFQIGA
                         @#          @                @
151 NKDQVMELTI AGMGTSALKV GSINIGSGKG AKSISGMIET IDNAINTVSG
201 ERAKLGANQN RLEHTIANLD NSAENLQAAE SRIRDVDMAK EMMNFTKTNI
251 LTQAAQAMLA QANQAPQGVL QLLRHHHHHH
```

m2. **Sequence coverage (shown by underlining) of trypsin digested *Cb*Fla co-expressed with *Gk*Maf with confidently assigned glycosites (validated by at least one MS/MS spectrum containing at least one glycan-modified fragment ion). Modifications with Neu5Ac and KDO are indicated by @ and #, respectively.**

```
                                             @
1   MAIINHNLNA MNAHRQMAIN TGSNGKAIEK LSSGLRINRA GDDAAGLAIS
51  EKMRGQIRGL NQASRNSQDS ISLIQTAEGA LNETHSILQR MRELAVQSAN
101 DTNVKVDRDN LQKEVAELQN EINRIASQTQ FNTKNLLNGS AKSMVFQIGA
                         @#          @
151 NKDQVMELTI AGMGTSALKV GSINIGSGKG AKSISGMIET IDNAINTVSG
201 ERAKLGANQN RLEHTIANLD NSAENLQAAE SRIRDVDMAK EMMNFTKTNI
251 LTQAAQAMLA QANQAPQGVL QLLRHHHHHH
```

m3. **Domain architecture of full length *Cb*Fla. ND1a and CD1 have been partitioned into ND1a1, ND1a2, and ND1a3, and CD1_1, CD1_2 and CD1_3, respectively and colored differently. Glycosites with Neu5Ac and KDO are indicated by symbols (♦) and (⬡), respectively.**

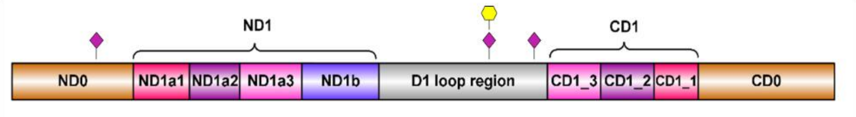

m4. MS/MS spectrum for a trypsin digested peptide from *Cb*Fla co-expressed with *Gk*Maf

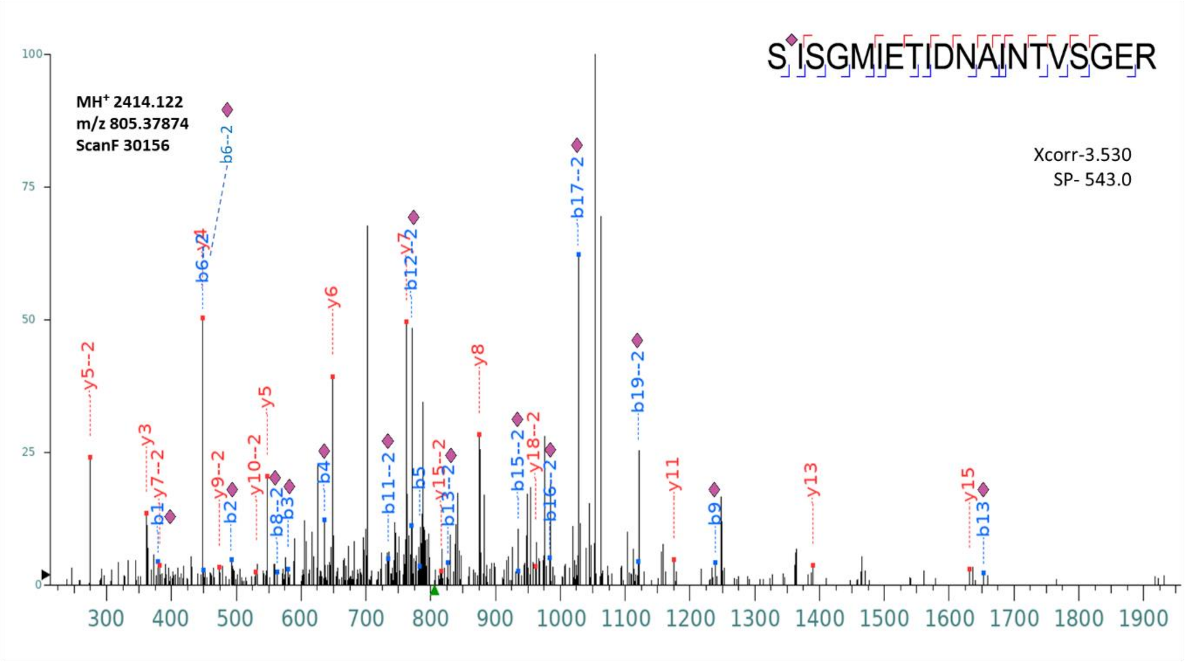

m5. MS/MS spectrum for a trypsin digested peptide from *Cb*Fla co-expressed with *Gk*Maf

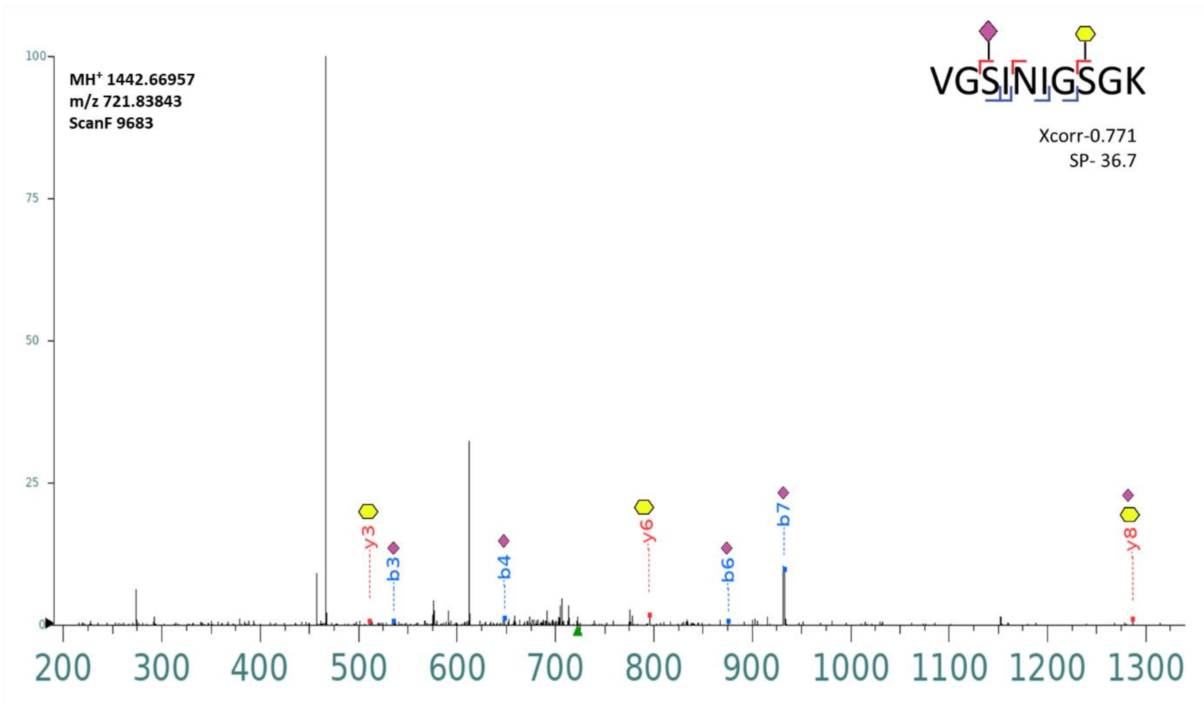

m6. MS/MS spectrum for a trypsin digested peptide from *Cb*Fla co-expressed with *Gk*Maf

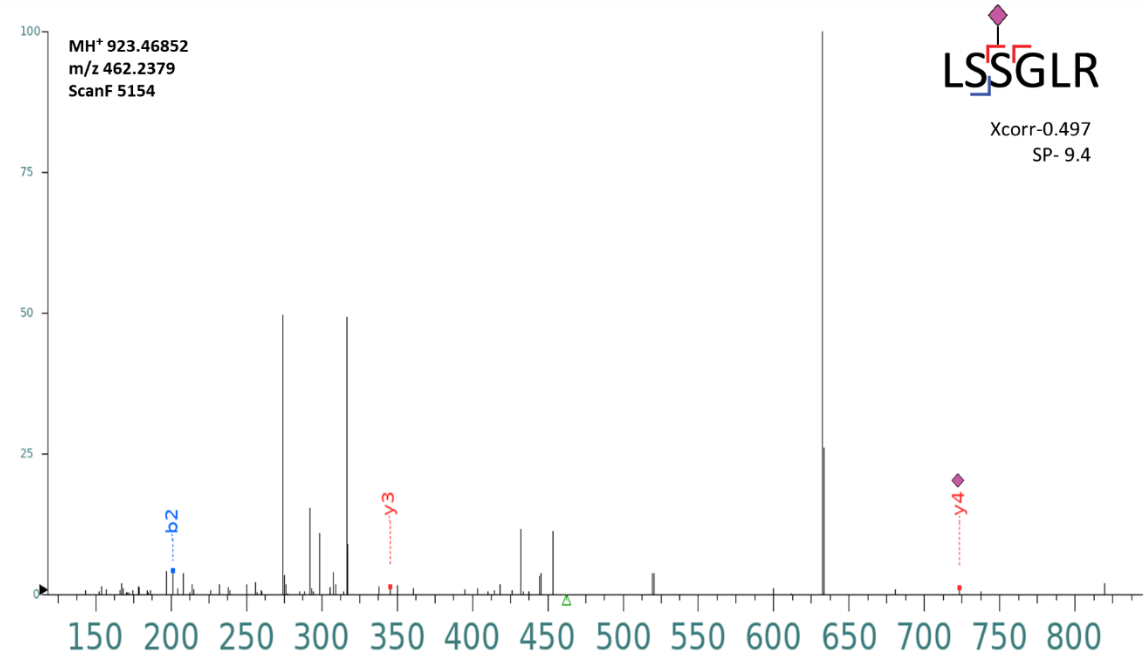

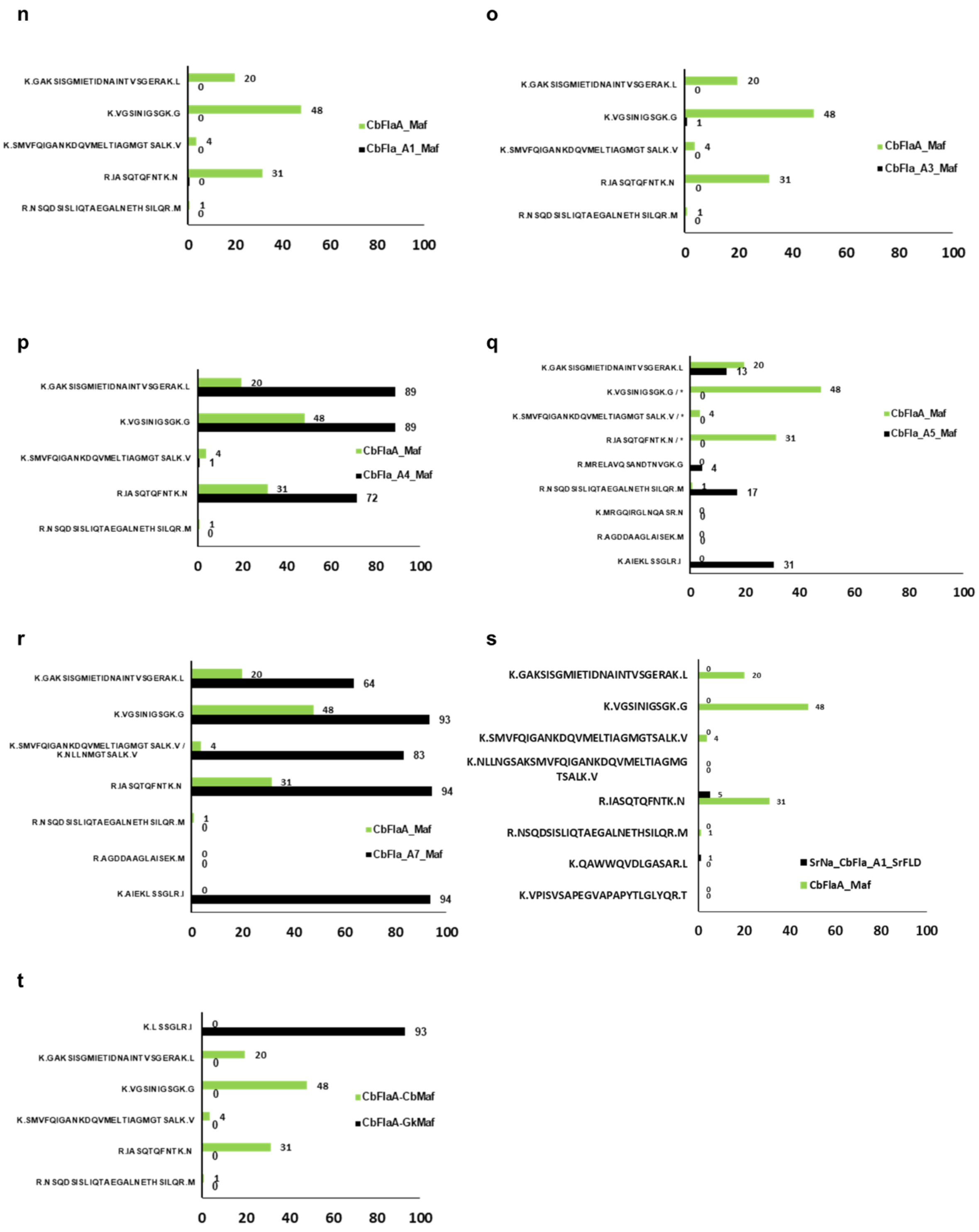
n
K.GAKSISGMIETIDNAINTVSGERAK.L
K.VGSINIGSGK.G
K.SMVFQIGANKDQVMELTIAGMGTSALK.V
R.IASQTQFNTK.N
R.NSQDSISLIQTAEGALNETHSILQR.M
CbFlaA_Maf
CbFla_A1_Maf
o
CbFlaA_Maf
CbFla_A3_Maf
p
CbFlaA_Maf
CbFla_A4_Maf
q
K.VGSINIGSGK.G / *
K.SMVFQIGANKDQVMELTIAGMGTSALK.V / *
R.IASQTQFNTK.N / *
R.MRELAVQSANDTNVGK.G
K.MRGQIRGLNQASR.N
R.AGDDAAGLAISEK.M
K.AIEKLSSGLR.I
CbFlaA_Maf
CbFla_A5_Maf
r
K.SMVFQIGANKDQVMELTIAGMGTSALK.V / K.NLLNMGTSALK.V
CbFlaA_Maf
CbFla_A7_Maf
s
K.NLLNGSAKSMVFQIGANKDQVMELTIAGMGTSALK.V
K.QAWWQVDLGASAR.L
K.VPISVSAPEGVAPAPYTLGLYQR.T
SrNa_CbFla_A1_SrFLD
CbFlaA_Maf
t
K.LSSGLR.I
CbFlaA-CbMaf
CbFlaA-GkMaf
0 20 40 60 80 100

u. Glycosites identified in *Cb*Fla. Domain architecture of full length *Cb*Fla. ND1a and CD1 have been partitioned into ND1a1, ND1a2, and ND1a3, and CD1_1, CD1_2 and CD1_3, respectively and colored differently. Glycosites with Neu5Ac, KDO and legionaminic acid derivative are indicated by symbols (♦) , (⬢) and (♦), respectively.

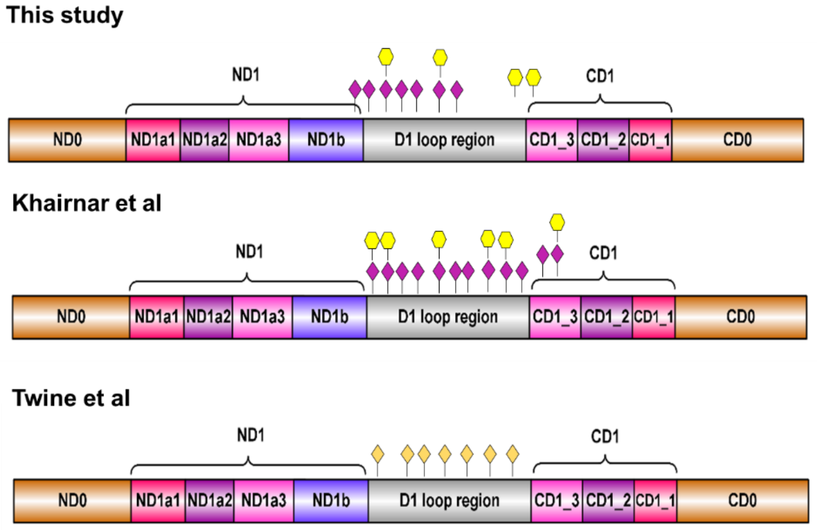

v. pLogo analysis of the glycosylation motif in *Cb*Fla. The foreground used was the set of 11-amino acid peptides containing the glycosite in the middle (sixth position), and the background used was all possible 11-amino acid sequences of CbFla (excluding those used as foreground).

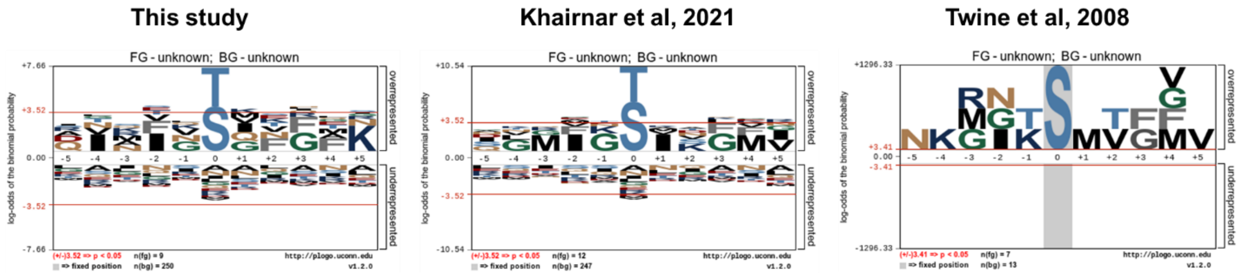

w. pLogo analysis of the amino acid sequence neighborhood in glycosites and non-glycosylated Ser/Thr with all reported glycosites of *Cb*Fla (Twine et al, Khairnar et al, and this study). The foreground used was the set of 11-amino acid peptides containing the glycosite in the middle (sixth position), or the set of 11-amino acid peptides with non-glycosylated Ser/Thr in the middle (sixth position), and the background used was all possible 11-amino acid sequences of *Cb*Fla (excluding those used as foreground).

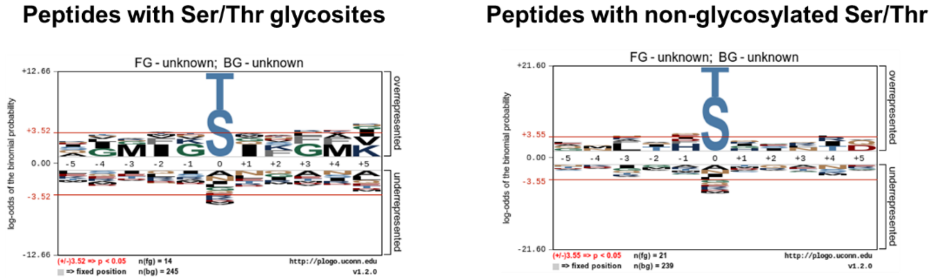

x. pLogo analysis of the amino acid sequence neighborhood in glycosites compared to non-glycosylated Ser/Thr. The foreground used was the set of 11-amino acid peptides containing the glycosite in the middle (sixth position), and the background used was all possible 11-amino acid peptides with non-glycosylated Ser/Thr in the middle (sixth position).

This study

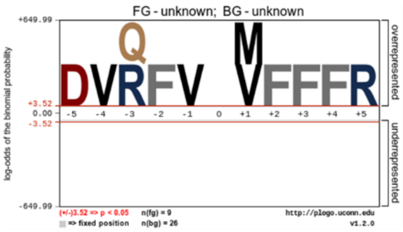

Khairnar et al, 2021

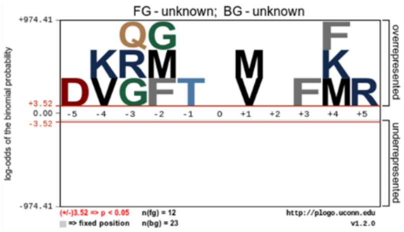

Twine et al, 2008

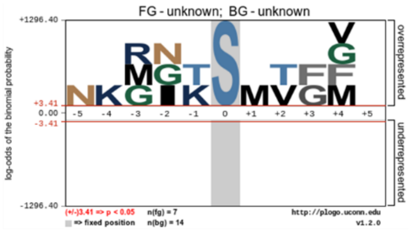

All studies together

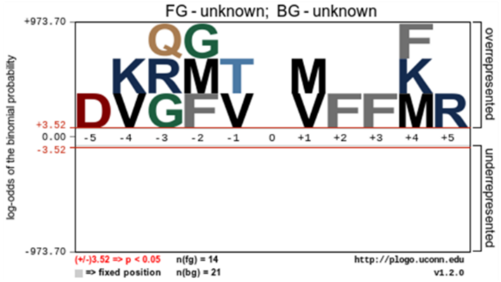

Supplementary figure 3: Tandem mass spectrometry obtained sequence coverage (and MS/MS spectra and domain architecture images indicating glycosites, where relevant) of *Cb*Fla (**a1-a13**), *Cb*FlaA1 (**b1,b2**), *Cb*FlaA3 (**c1-c5**), *Cb*FlaA4 (**d1-d9**), *Cb*FlaA5 (**e1-e14**), *Cb*FlaA7 (**f1-f15**), *Cb*FlaA4_A5 (**g1,g2**), *Cb*FlaD0A7loop (**h1,h2**), *Sr*NaFLD (**i1,i2**), *Sr*Na*Cb*FlaA1*Sr*FLD (**j1-j7**), GKGAKS_*Sr*NaFLD (**k1,k2**), and *Cb*Fla_A1helix_GKGAKS_*Sr*NaFLD (**l1,l2**), each co-expressed with *Cb*Maf in EV136 cells, and *Cb*Fla co-expressed with *Gk*Maf in EV136 cells (**m1-m6**). (**n,o,p,q,r,s**) Comparison of precursor ion intensities of modified peptides obtained from the tandem mass spectrometry data of *Cb*FlaA1 (n), *Cb*FlaA3 (o), *Cb*FlaA4 (p), *Cb*FlaA5 (q), *Cb*FlaA5, *Cb*FlaA7 (r), *Sr*Na_*Cb*FlaA1_*Sr*FLD (s) (all shown in black) and *Cb*Fla (shown in green). (**t**) Comparison of precursor ion intensities of modified peptides obtained from the tandem mass spectrometry data of *Cb*Fla co-expressed with *Gk*Maf (shown in black) and *Cb*Fla co-expressed with *Cb*Maf (shown in green). Tandem mass spectrometry data is a result of a single run. Modscore algorithm was used to identify peptides modified with 220 Da (KDO, represented by # symbol) or 291 Da (Neu5Ac, represented by @ symbol) on Ser/Thr sites of *Cb*Fla co-expressed with CbMaf. Glycosites validated by at least one MS/MS spectrum containing at least one glycan-modified fragment ion are considered confidently assigned glycosites.(**u**) Glycosites identified in CbFla. Domain architecture of full length CbFla. ND1a and

CD1 have been partitioned into ND1a1, ND1a2, and ND1a3, and CD1_1, CD1_2 and CD1_3, respectively and colored differently. Glycosites with Neu5Ac and KDO are indicated by their respective symbols. (**v-x**) pLogo analysis of the glycosylation motif in CbFla, with the parameters mentioned in the figure headings.

Supplementary Figure 4

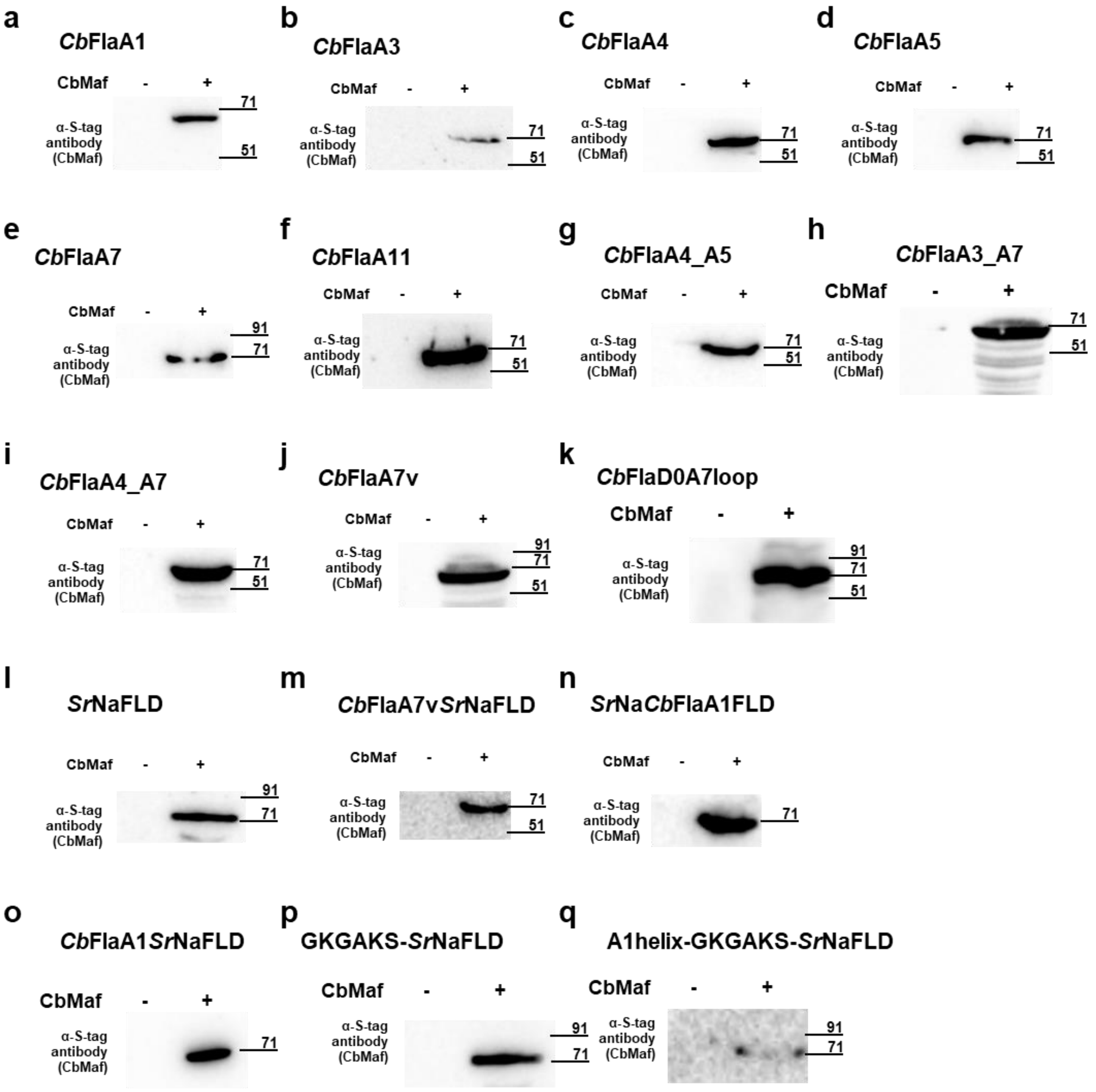

Supplementary figure 4: Western blot analysis of recombinant *Cb*Maf co-expressed with *Cb*Fla constructs in EV136 cells: *Cb*FlaA1 (**a**), *Cb*FlaA3 (**b**), *Cb*FlaA4 (**c**), *Cb*FlaA5 (**d**), *Cb*FlaA7 (**e**), *Cb*FlaA11 (**f**), *Cb*FlaA4_A5 (**g**), *Cb*FlaA3_A7 (**h**), *Cb*FlaA4_A7 (**i**), *Cb*FlaA7v (**j**), *Cb*FlaD0A7loop (**k**), *Sr*NaFLD (**l**), *Cb*FlaA7v*Sr*NaFLD (**m**), *Sr*Na*Cb*FlaA1*Sr*FLD (**n**), *Cb*FlaA1*Sr*NaFLD (**o**), GKGAKS_*Sr*NaFLD (**p**), *Cb*Fla_A1helix_GKGAKS_*Sr*NaFLD (**q**). *Cb*Maf was expressed in each case from pCDF-Duet1 vector and was detected with anti-S-tag antibody.

Supplementary Figure 5

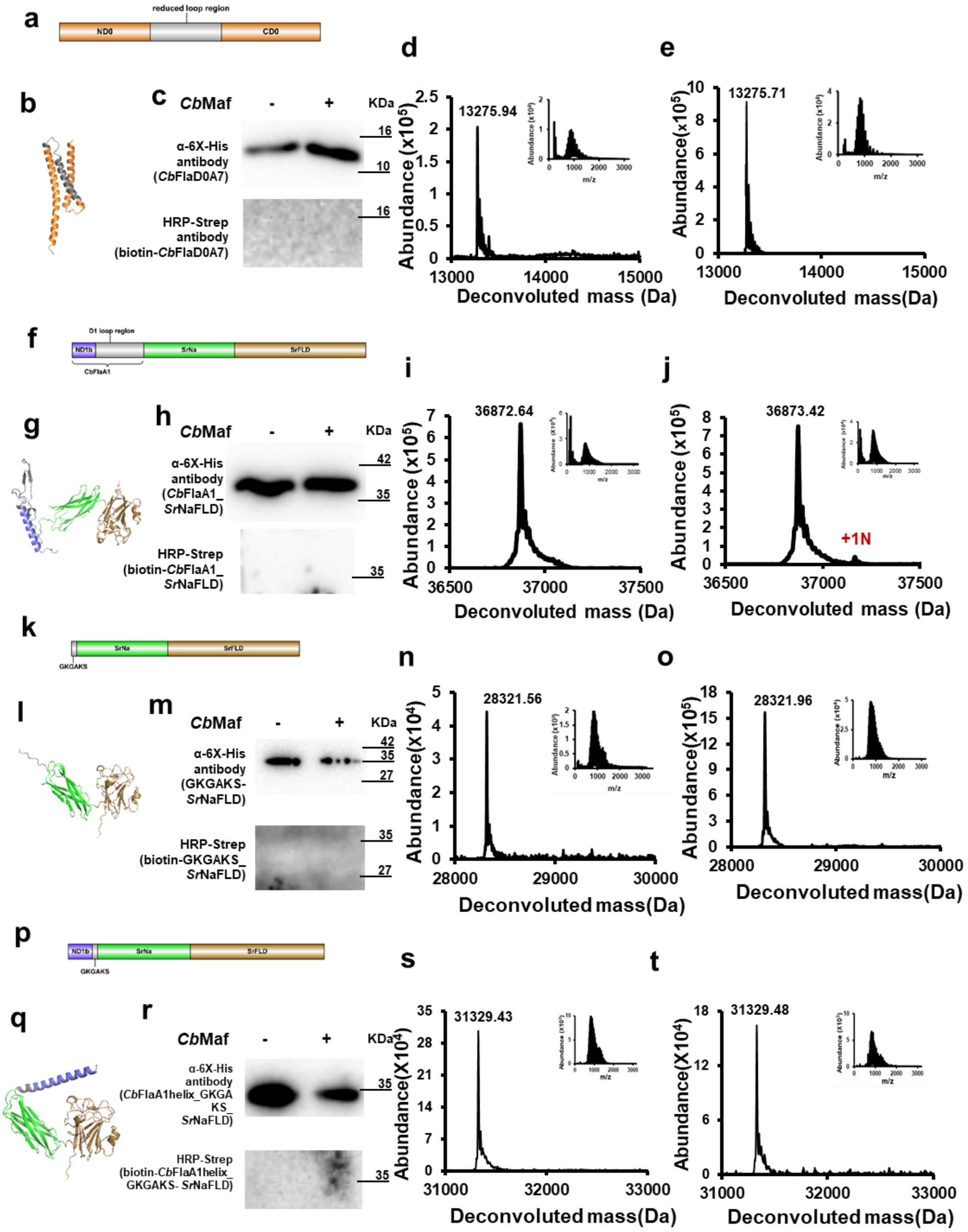

Supplementary figure 5: Scarce or no sialylation of recombinant *Cb*Fla deletion construct and recombinant chimeras of *Cb*Fla deletions with *Sr*NaFLD by co-expressed

***Cb*Maf in *E. coli* EV136 cells cultured in minimal medium.** (**a,f,k,p**) Domain architectures of *Cb*FlaD0A7loop (a), *Cb*FlaA1_*Sr*NaFLD (f), GKGAKS_*Sr*NaFLD (k) and *Cb*Fla_A1helix_GKGAKS_*Sr*NaFLD (p). (**b,g,l,q**) AlphaFold2-predicted structural models of *Cb*FlaD0A7loop (b), *Cb*FlaA1_*Sr*NaFLD (g), GKGAKS_*Sr*NaFLD (l) and *Cb*Fla_A1helix_GKGAKS_*Sr*NaFLD (q). (**c,h,m,r**) Western blot analysis of recombinant *Cb*FlaD0A7loop (c), *Cb*FlaA1_*Sr*NaFLD (h), GKGAKS_*Sr*NaFLD (m) and *Cb*Fla_A1helix_GKGAKS_*Sr*NaFLD (r) expressed from pET-28a(+) vector in EV136 cells detected with mouse anti-6XHis antibody (upper panels in each). On-blot periodate oxidation and aniline-catalyzed ligation with aminooxy biotin of *Cb*FlaD0A7loop (c), *Cb*FlaA1_*Sr*NaFLD (h), GKGAKS_*Sr*NaFLD (m) and *Cb*Fla_A1helix_GKGAKS_*Sr*NaFLD (r) singly expressed or co-expressed with *Cb*Maf in EV136 cells. Biotinylation was detected with HRP–streptavidin (lower panels in each). (**d,e,i,j,n,o,s,t**) Intact mass measurements of *Cb*FlaD0A7loop, *Cb*FlaA1_*Sr*NaFLD, GKGAKS_*Sr*NaFLD and *Cb*Fla_A1helix_GKGAKS_*Sr*NaFLD expressed singly (**d,l,n,s**) or co-expressed with *Cb*Maf (**e,j,o,t**) and purified from EV136 cells. The proteins were acetone precipitated and subjected to LC–MS in positive mode ionization. Insets show the ionization spectra of *Cb*FlaD0A7loop, *Cb*FlaA1_*Sr*NaFLD, GKGAKS_*Sr*NaFLD and *Cb*Fla_A1helix_GKGAKS_*Sr*NaFLD.

Supplementary Figure 6

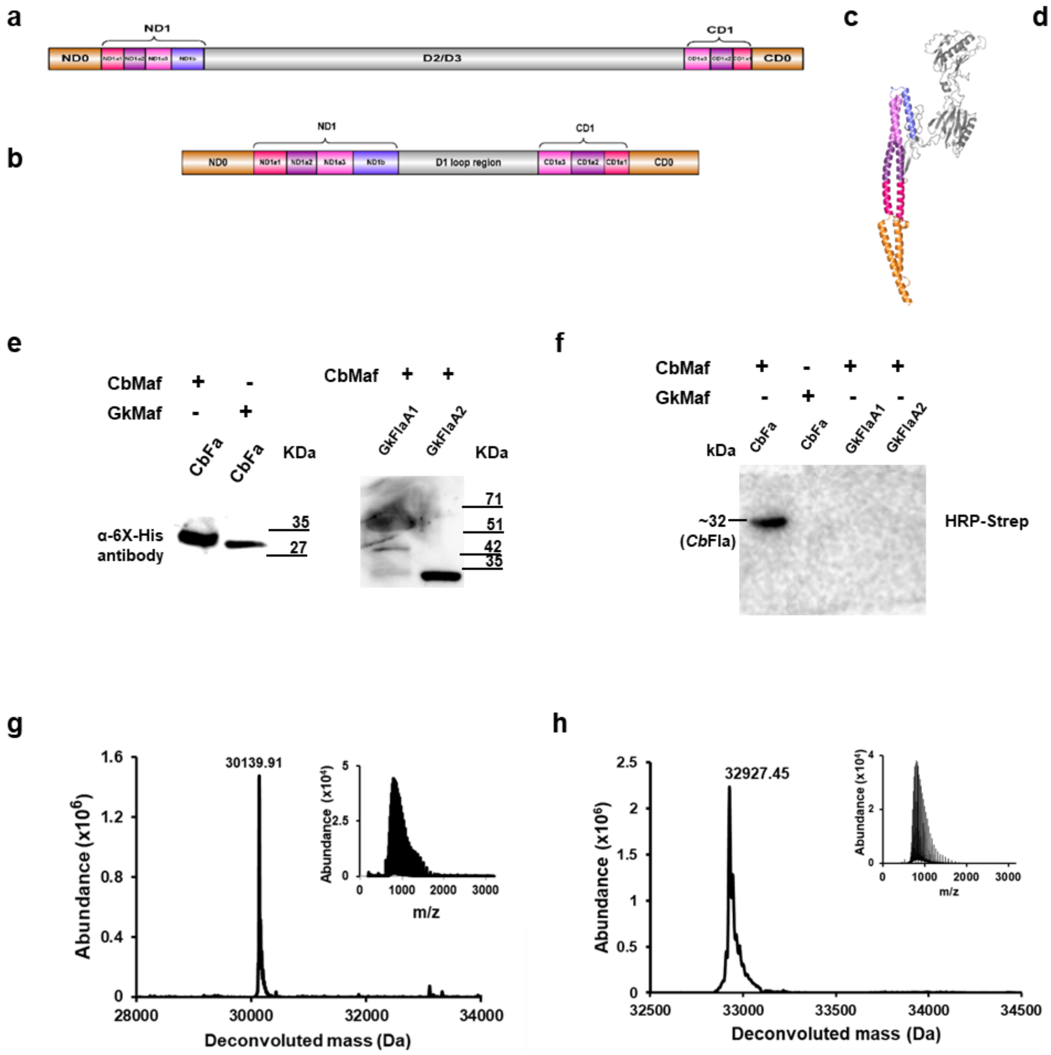

Supplementary figure 6: Acceptor substrate promiscuity of *Cb*Maf and *Gk*Maf. (**a,b**) Domain architectures of *Gk*FlaA1 (a) and *Gk*FlaA2 (b). (**c,d**) AlphaFold2-predicted structural models of *Gk*FlaA1 (c) and *Gk*FlaA2 (d). (**e**) Western blot analysis of recombinant *Cb*Fla, *Gk*FlaA1 and *Gk*FlaA2 expressed from pET-28a(+) vector in EV136 cells detected with mouse anti-6XHis antibody. *Cb*Fla co-expressed with *Cb*Maf was used as a positive control. (**f**) On-blot periodate oxidation and aniline-catalyzed ligation with aminooxy biotin of *CbFla* and GkFlaA2 co-expressed with *Gk*Maf and *Cb*Maf in EV136 cells. Biotinylation was detected with HRP–streptavidin. *Cb*Fla co-expressed with *Cb*Maf was used as a positive control. For this blot, only one size marker (*Cb*Fla in lane 1) is available as the visible light image with the pre-stained molecular marker was inadvertently not saved; the molecular mass range of the proteins on the blot may be approximated from the anti-6XHis gels run in parallel (**e**) (**g,h**)

Intact mass measurements of *Cb*Fla co-expressed with *Gk*Maf (g) and *Gk*FlaA2 co-expressed with *Cb*Maf (h) purified from *E. coli* EV136 cells. The insets show the ionization spectra of *Cb*Fla and *Gk*FlaA2.

Supplementary Figure 7

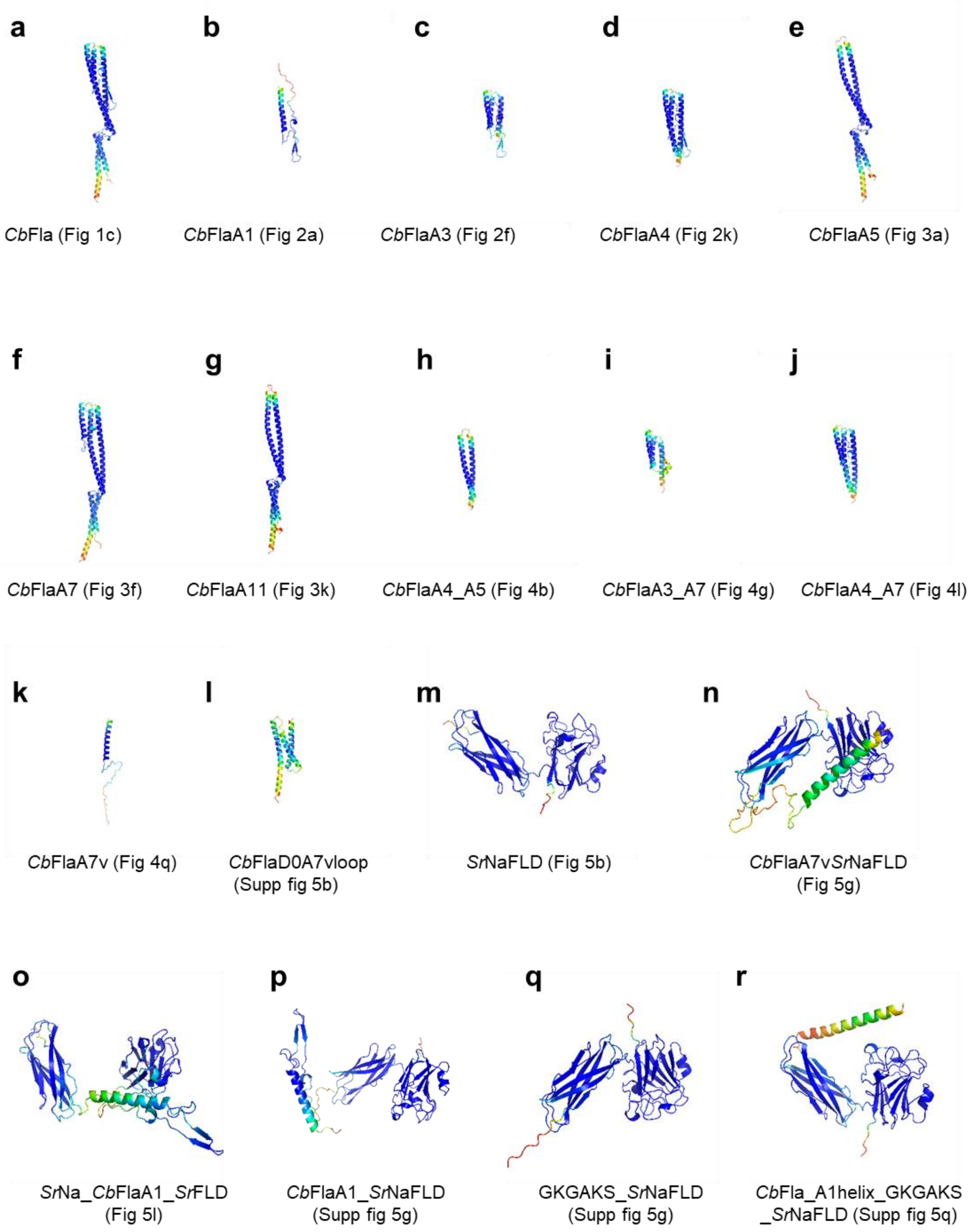

Supplementary Figure 7: AlphaFold2-predicted structural models of constructs used in this study visualized with reverse rainbow coloring as per pLDDT scores in PyMOL (Schrodinger).

Supplementary Figure 8

Blots from Figure 1

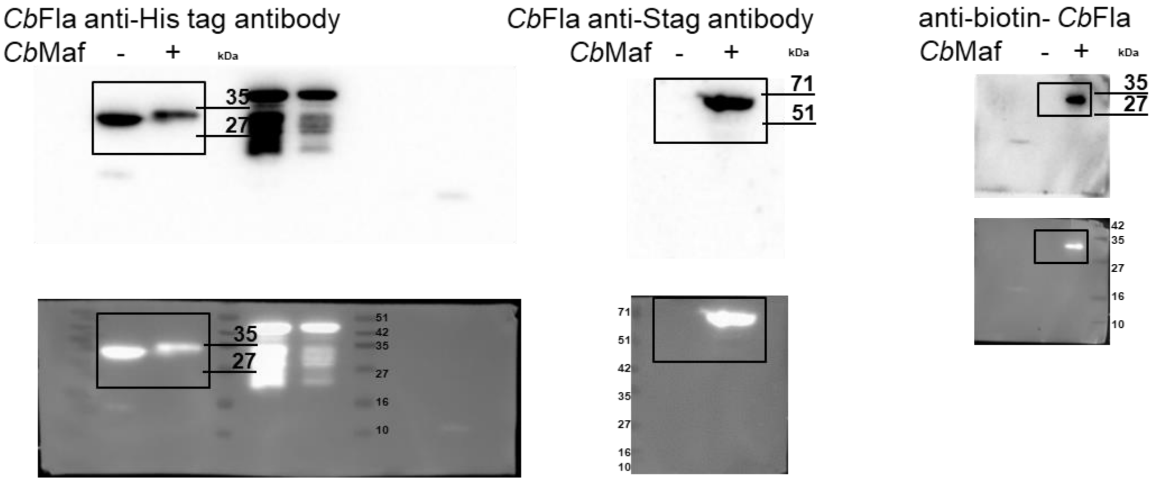

The image in the outlined boxes from each blot in the upper panel was used in Figure 1d. The lower panels are the composite images (visible light plus chemiluminescence) showing the pre-stained molecular marker along with the inverted images of the upper panels.

Blots from Figure 2c

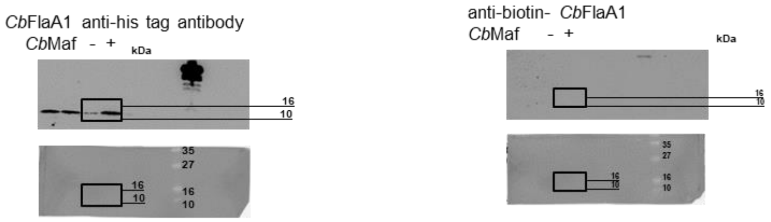

The image in the outlined boxes from each blot in the upper panel was used in Figure 2c. The lower panels are the composite images (visible light plus chemiluminescence) showing the pre-stained molecular marker along with the inverted images of the upper panels.

Blots from Figure 2h

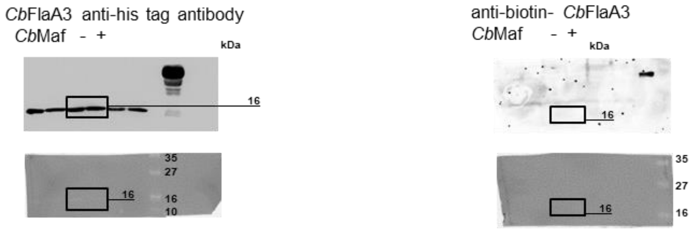

The image in the outlined boxes from each blot in the upper panel was used in Figure 2h. The lower panels are the composite images (visible light plus chemiluminescence) showing the pre-stained molecular marker along with the inverted images of the upper panels.

Blots from Figure 2m

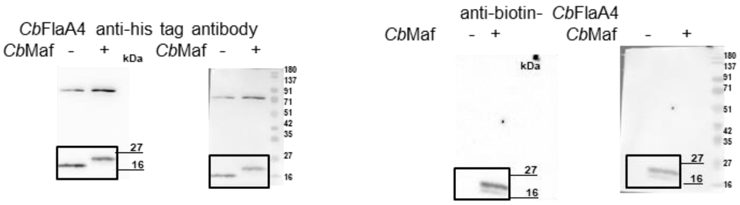

The image in the outlined boxes from each blot in the left panel was used in Figure 2m. The right panels are the composite images (visible light plus chemiluminescence) showing the pre-stained molecular marker along with the inverted images of the left panels.

Blots from Figure 4c

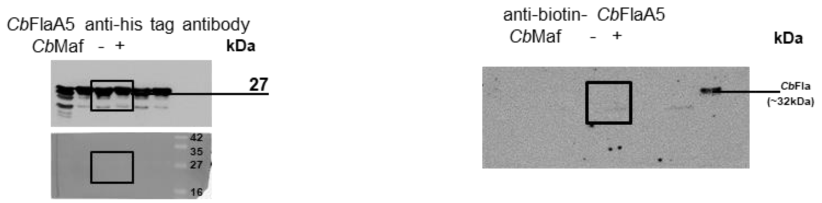

The image in the outlined boxes from each blot in the upper/only panel was used in Figure 4c. The left lower panel, is the composite image (visible light plus chemiluminescence) showing the pre-stained molecular marker along with the inverted image of the upper panel.

Blots from Figure 4h

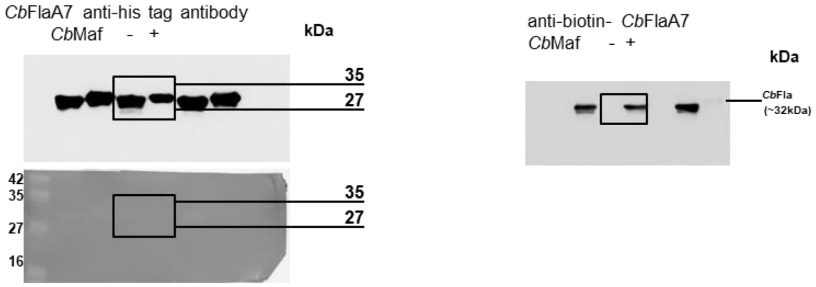

The image in the outlined boxes from each blot in the upper/only panel was used in Figure 4h. The left lower panel is the composite image (visible light plus chemiluminescence) showing the pre-stained molecular marker along with the inverted image of the upper panel.

Blots from Figure 4m

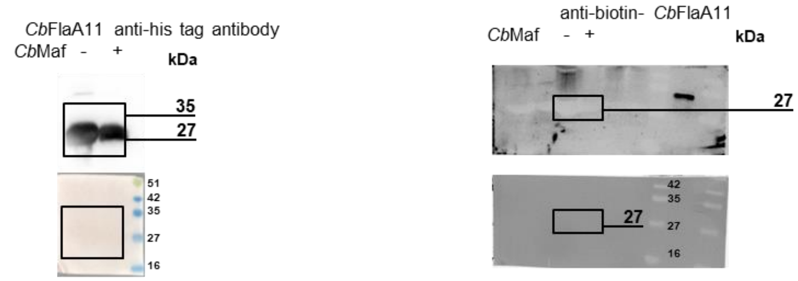

The image in the outlined boxes from each blot in the upper panel was used in Figure 4m. The left lower panel is the visible light image showing the pre-stained molecular marker, and the right lower panel is the composite image (visible light plus chemiluminescence) showing the pre-stained molecular marker along with the inverted image of the upper panel.

Blots from Figure 5c

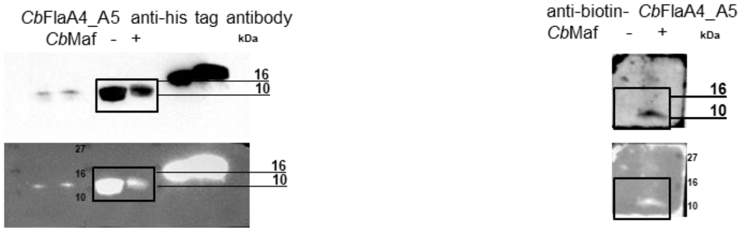

The image in the outlined boxes from each blot in the upper panel was used in Figure 5c. The lower panels are the composite images (visible light plus chemiluminescence) showing the pre-stained molecular marker along with the inverted images of the upper panels.

Blots from Figure 5h

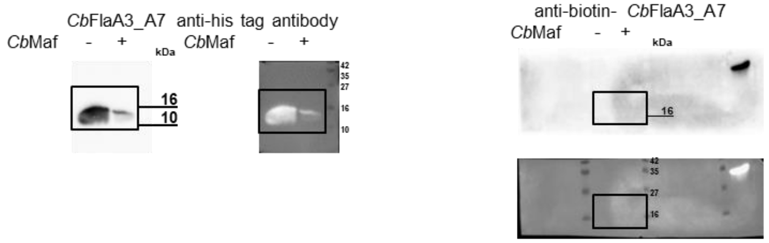

The image in the outlined boxes from each blot in the upper panel was used in Figure 5h. The lower panels are the composite images (visible light plus chemiluminescence) showing the pre-stained molecular marker along with the inverted images of the upper panels.

Blots from Figure 5m

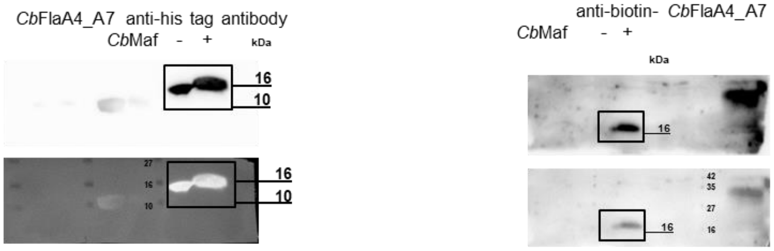

The image in the outlined boxes from each blot in the upper panel was used in Figure 5m. The lower panels are the composite images (visible light plus chemiluminescence) showing the pre-stained molecular marker along with the inverted images of the upper panels.

Blots from Figure 5r

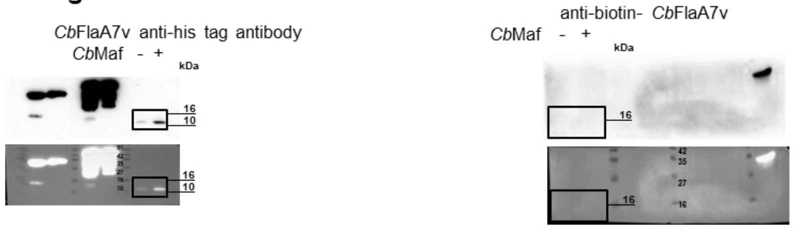

The image in the outlined boxes from each blot in the upper panel was used in Figure 5r. The lower panels are the composite images (visible light plus chemiluminescence) showing the pre-stained molecular marker along with the inverted images of the upper panels.

Blots from Figure 6c

*Sr*NaFLD anti-his tag antibody
*Cb*Maf - + kDa

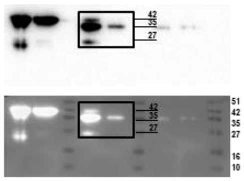

anti-biotin- *Sr*NaFLD
*Cb*Maf - + kDa

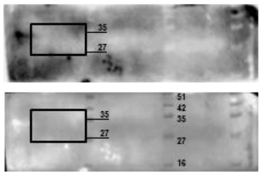

The image in the outlined boxes from each blot in the upper panel was used in Figure 6c. The lower panels are the composite images (visible light plus chemiluminescence) showing the pre-stained molecular marker along with the inverted images of the upper panels.

Blots from Figure 6h

*Cb*FlaA7v*Sr*NaFLD anti-his tag antibody
*Cb*Maf - + kDa

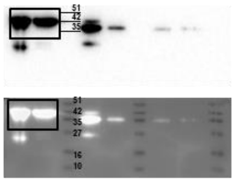

anti-biotin- *Cb*FlaA7v*Sr*NaFLD
*Cb*Maf - + kDa

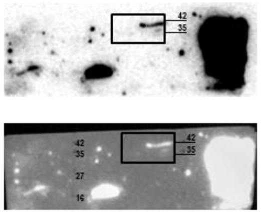

The image in the outlined boxes from each blot in the upper panel was used in Figure 6h. The lower panels are the composite images (visible light plus chemiluminescence) showing the pre-stained molecular marker along with the inverted images of the upper panels.

Blots from Figure 6m

*Sr*Na*Cb*FlaA1*Sr*FLD anti-his tag antibody
*Cb*Maf - + kDa

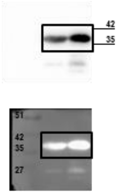

anti-biotin- *Sr*Na*Cb*FlaA1*Sr*FLD
*Cb*Maf - + kDa

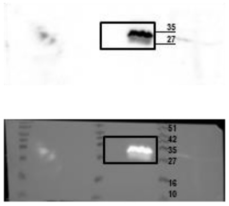

The image in the outlined boxes from each blot in the upper panel was used in Figure 6m. The lower panels are the composite images (visible light plus chemiluminescence) showing the pre-stained molecular marker along with the inverted images of the upper panels.

Blot from Supplementary figure 4a

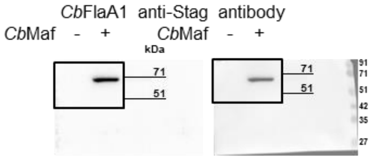

The image in the outlined box from the blot in the left panel was used in Supplementary figure 4a. The right panel is the composite image (visible light plus chemiluminescence) showing the pre-stained molecular marker along with the inverted image of the left panel.

Blot from Supplementary figure 4b

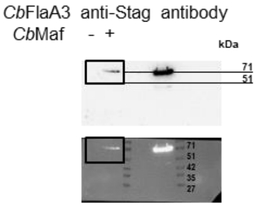

The image in the outlined box from the blot in the upper panel was used in Supplementary figure 4b. The lower panel is the composite image (visible light plus chemiluminescence) showing the pre-stained molecular marker along with the inverted image of the upper panel.

Blot from Supplementary figure 4c

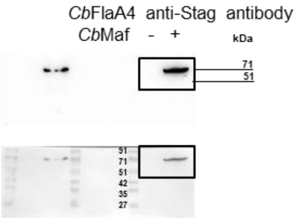

The image in the outlined box from the blot in the upper panel was used in Supplementary figure 4c. The lower panel is the composite image (visible light plus chemiluminescence) showing the pre-stained molecular marker along with the inverted image of the upper panel.

Blot from Supplementary figure 4d

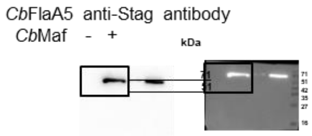

The image in the outlined box from the blot in the left panel was used in Supplementary figure 4d. The right panel is the composite image (visible light plus chemiluminescence) showing the pre-stained molecular marker along with the inverted image of the left panel.

Blot from Supplementary figure 4e

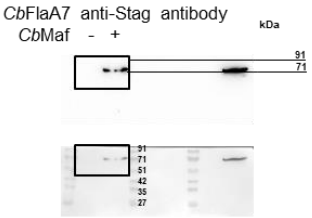

The image in the outlined box from the blot in the upper panel was used in Supplementary figure 4e. The lower panel is the composite image (visible light plus chemiluminescence) showing the pre-stained molecular marker along with the inverted image of the upper panel.

Blot from Supplementary figure 4f

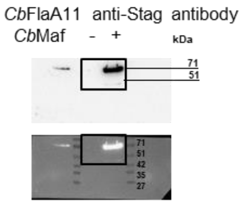

The image in the outlined box from the blot in the upper panel was used in Supplementary figure 4f. The lower panel is the composite image (visible light plus chemiluminescence) showing the pre-stained molecular marker along with the inverted image of the upper panel.

Blot from Supplementary figure 4g

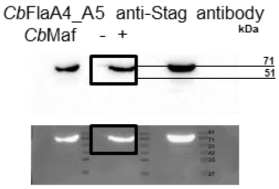

The image in the outlined box from the blot in the upper panel was used in Supplementary figure 4g. The lower panel is the composite image (visible light plus chemiluminescence) showing the pre-stained molecular marker along with the inverted image of the upper panel.

Blot from Supplementary figure 4h

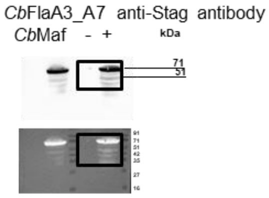

The image in the outlined box from the blot in the upper panel was used in Supplementary figure 4h. The lower panel is the composite image (visible light plus chemiluminescence) showing the pre-stained molecular marker along with the inverted image of the upper panel.

Blot from Supplementary figure 4i

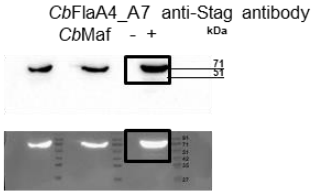

The image in the outlined box from the blot in the upper panel was used in Supplementary figure 4i. The lower panel is the composite image (visible light plus chemiluminescence) showing the pre-stained molecular marker along with the inverted image of the upper panel.

Blot from Supplementary figure 4j

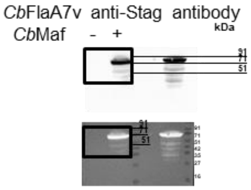

The image in the outlined box from the blot in the upper panel was used in Supplementary figure 4j. The lower panel is the composite image (visible light plus chemiluminescence) showing the pre-stained molecular marker along with the inverted image of the upper panel.

Blot from Supplementary figure 4k

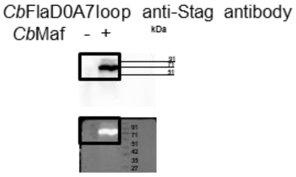

The image in the outlined box from the blot in the upper panel was used in Supplementary figure 4k. The lower panel is the composite image (visible light plus chemiluminescence) showing the pre-stained molecular marker along with the inverted image of the upper panel.

Blot from Supplementary figure 4l

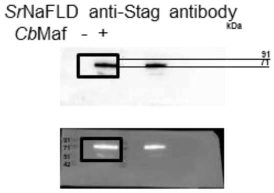

The image in the outlined box from the blot in the upper panel was used in Supplementary figure 4l. The lower panel is the composite image (visible light plus chemiluminescence) showing the pre-stained molecular marker along with the inverted image of the upper panel.

Blot from Supplementary figure 4m

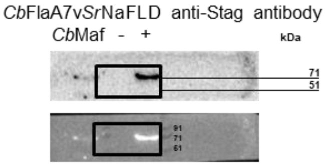

The image in the outlined box from the blot in the upper panel was used in Supplementary figure 4m. The lower panel is the composite image (visible light plus chemiluminescence) showing the pre-stained molecular marker along with the inverted image of the upper panel.

Blot from Supplementary figure 4n

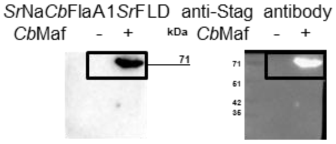

The image in the outlined box from the blot in the left panel was used in Supplementary figure 4n. The right panel is the composite image (visible light plus chemiluminescence) showing the pre-stained molecular marker along with the inverted image of the left panel.

Blot from Supplementary figure 4o

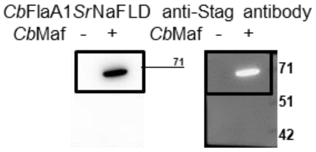

The image in the outlined box from the blot in the left panel was used in Supplementary figure 4o. The right panel is the composite image (visible light plus chemiluminescence) showing the pre-stained molecular marker along with the inverted image of the left panel.

Blot from Supplementary figure 4p

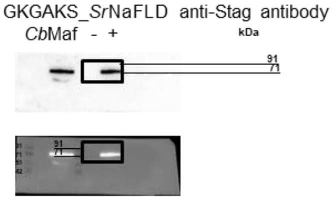

The image in the outlined box from the blot in the upper panel was used in Supplementary figure 4p. The lower panel is the composite image (visible light plus chemiluminescence) showing the pre-stained molecular marker along with the inverted image of the upper panel.

Blot from Supplementary figure 4q

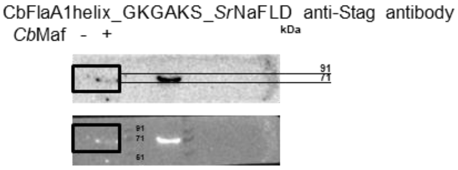

The image in the outlined box from the blot in the upper panel was used in Supplementary figure 4q. The lower panel is the composite image (visible light plus chemiluminescence) showing the pre-stained molecular marker along with the inverted image of the upper panel.

Blots from Supplementary figure 5c

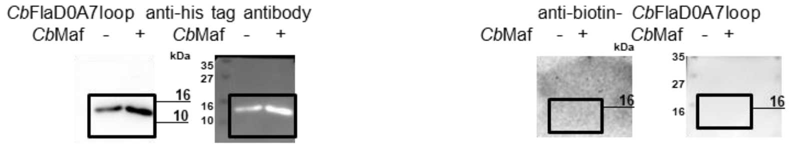

The image in the outlined boxes from each blot in the left panel was used in Supplementary figure 5c. The right panels are the composite images (visible light plus chemiluminescence) showing the pre-stained molecular marker along with the inverted images of the left panels.

Blots from Supplementary figure 5h

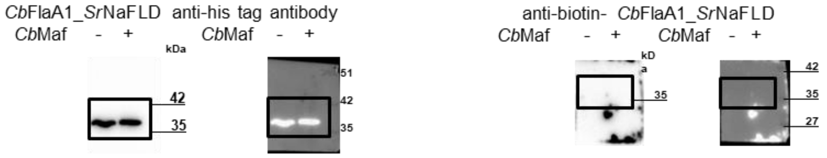

The image in the outlined boxes from each blot in the left panel was used in Supplementary figure 5h. The right panels are the composite images (visible light plus chemiluminescence) showing the pre-stained molecular marker along with the inverted images of the left panels.

Blots from Supplementary figure 5m

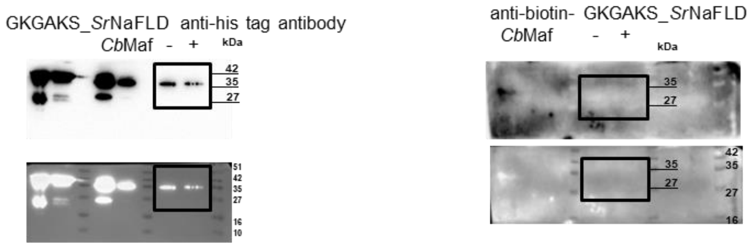

The image in the outlined boxes from each blot in the upper panel was used in Supplementary figure 5m. The lower panels are the composite images (visible light plus chemiluminescence) showing the pre-stained molecular marker along with the inverted images of the upper panels.

Blots from Supplementary figure 5r

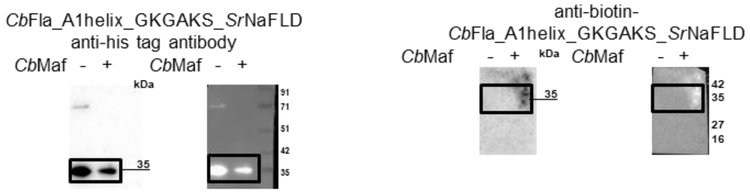

The image in the outlined boxes from each blot in the left panel was used in Supplementary figure 5r. The right panels are the composite images (visible light plus chemiluminescence) showing the pre-stained molecular marker along with the inverted images of the left panels.

Blots from Supplementary figure 6e

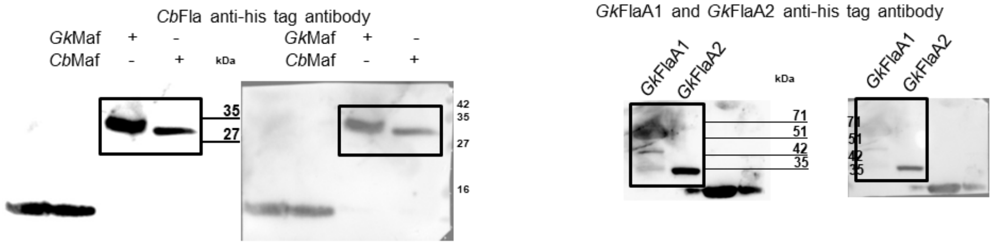

The images in the outlined boxes from each blot in the left panel was used in Supplementary figure 6e. The right panels are the composite images (visible light plus chemiluminescence) showing the pre-stained molecular marker along with the inverted images of the left panels.

Blot from Supplementary figure 6f

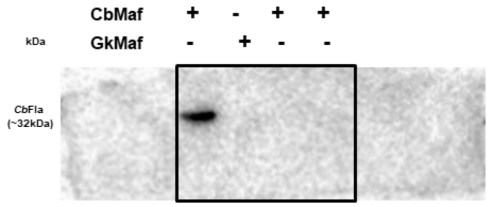

The image in the outlined box from this blot was used in Supplementary figure 6f.

Supplementary Figure 8: Images of blots indicating the digitally cropped regions of blots shown in the figures and supplementary figures in this paper.
