# Supplementary material for: Harnessing the acceptor substrate promiscuity of Clostridium botulinum Maf glycosyltransferase to glyco-engineer mini-flagellin protein chimeras
Source: Commun Biol. 2024 Aug 21;7:1029. doi: 10.1038/s42003-024-06736-y (PMC11339370; doi:10.1038/s42003-024-06736-y)
Supplement: Supplementary file 3 — Description of Additional Supplementary File [file 42003_2024_6736_MOESM3_ESM.pdf]

Description Of Additional Supplementary File

File name: Supplementary Data 1
Description: Contains Tables S1 to S47

File name: Supplementary Data 2
Description: Zipped folder of Excel files containing the source data behind the graphs in the paper

File name: Supplementary Data 3
Description: Zipped folder of .pdb files with the AlphaFold2-predicted structural models reported in the paper
